# Clinical proof of concept for small molecule mediated inhibition of IL-17 in psoriasis

**DOI:** 10.1371/journal.pone.0341049

**Published:** 2026-01-23

**Authors:** Richard B. Warren, Hamish J. A. Hunter, Kim A. Papp, Kenneth B. Gordon, Meina T. Tang, Jeffrey V. Enejosa, Kuan-Chieh Huang, Debasish Raha, Hashini M. Batugedara, Craig Skinner, Philip A. Kobel, Peter M. Rademacher, Nico Ghilardi, Paul Fatheree, John R. Jacobsen, Timothy T. Lu

**Affiliations:** 1 Dermatology Centre, Northern Care Alliance NHS Foundation Trust, Manchester, United Kingdom; 2 NIHR Manchester Biomedical Research Centre, Manchester University NHS Foundation Trust, Manchester Academic Health Science Centre, Manchester, United Kingdom; 3 Medicines Evaluation Unit, Manchester, United Kingdom; 4 Alliance Clinical Research and Probity Medical Research, Waterloo, Ontario, Canada; 5 Division of Dermatology, Department of Medicine, University of Toronto, Toronto, Ontario, Canada; 6 Dermatology, Medical College of Wisconsin, Wauwatosa, Wisconsin, United States of America; 7 DICE Therapeutics, a wholly owned subsidiary of Eli Lilly and Company, South San Francisco, California, United States of America; Istituto Dermopatico dell'Immacolata (IDI)-IRCCS, ITALY

## Abstract

Efficacious and well-tolerated systemic, oral treatments for psoriasis are needed. We report preclinical and phase 1c (NCT06808815) results for DC-806, a small molecule interleukin (IL)-17 inhibitor, for the treatment of mild-to-moderate psoriasis. Preclinical results demonstrated DC-806 targets IL-17AA and IL-17AF with secukinumab-like therapeutic efficacy. In the phase 1c trial, 32 patients consented to receive twice daily (BID) doses of placebo or DC-806 (200 mg or 800 mg) for 28 days. No serious adverse events (SAEs) or discontinuations due to treatment-related adverse events (TRAEs) occurred. In an exploratory analysis, adjusted mean percentage reductions from baseline in psoriasis area and severity indices (PASI) at Day 29 were 43.7%, 15.1%, and 13.3% for 800 mg BID, 200 mg BID, and placebo arms, respectively (800 mg BID vs placebo, *P* value = 0.0008). DC-806 was found to be well tolerated with an acceptable safety profile and preliminary signals of clinical efficacy in mild-to-moderate psoriasis. EudraCT Identifier: 2021-002888-21.

## Introduction

Psoriasis, a chronic, relapsing, inflammatory disease of the skin, affects an estimated 60 million people worldwide and is associated with substantial physical, psychological, social, and economic burden [1–3]. The most common form of psoriasis, plaque psoriasis, is defined by red, scaly plaques that commonly appear on extensor surfaces such as the elbows and knees, gluteal folds, scalp, and trunk. Immunological and genetic studies have identified the cytokine interleukin (IL)-17A as a key driver of psoriasis pathogenesis [3–6]. IL-17A is a member of the IL-17 cytokine family and is secreted by various lymphocytes, including T-helper 17 (TH17) cells, either as an IL-17A homodimer (IL-17AA) or as a heterodimer with IL-17F (IL-17AF) [7]. An IL-17FF homodimer with lower signaling potency compared to IL-17AA is also produced [7,8]. In psoriasis, abnormal activation of IL-17 signaling in skin keratinocytes promotes the secretion of inflammatory cytokines and antimicrobial peptides, resulting in neutrophil recruitment and aberrant proliferation and differentiation of skin keratinocytes [4].

Biologic therapies targeting IL-17, including the antibodies secukinumab (IL-17AA and IL-17AF), ixekizumab (IL-17AA and IL-17AF), bimekizumab (IL-17AA, IL-17AF, and IL-17FF), and brodalumab (IL-17 receptor A), are available for the treatment of psoriasis [9–12]. Nevertheless, patient preference, treatment cost, and access may impede biologic treatment adherence and thus limit benefit [13]. Orally bioavailable treatment options targeting a variety of mechanisms of action are also approved for use in psoriasis and include deucravacitinib, methotrexate, cyclosporine, and apremilast [14–17]. However, these oral treatment options are less efficacious than antibodies that target the IL-17 pathway [18,19] and have safety and tolerability concerns [14–17]. Accordingly, there is a large unmet need for efficacious and well-tolerated oral therapies for patients with psoriasis who require systemic therapy.

DC-806 (1-ethyl-N-((S)-2-((2-fluoro-4-((2S,3R)-4-(4-methylpiperazin-1-yl)-4-oxo-3-propionamidobutan-2-yl)phenyl)amino)-1-(trans-4-methylcyclohexyl)-2-oxoethyl)-1H-pyrazole-5-carboxamide) is an orally bioavailable small molecule targeting IL-17AA and IL-17AF, preventing their binding to the IL-17 receptor through an allosteric mode of action. Because this intervention is mechanistically highly similar to that of secukinumab and ixekizumab, we reasoned that treatment of psoriasis with DC-806 would be safe and effective, barring unexpected off-target toxicity not discovered in pre-clinical toxicity studies. Efficacy was evaluated first in multiple *in vitro* and preclinical *in vivo* models to help define a potentially efficacious dose range in patients. We then designed a first-in-human, 3-part, randomized, double-blind, placebo-controlled, phase 1 clinical trial (NCT06808815) to evaluate the safety and tolerability, pharmacokinetics (PK), and clinical activity of DC-806 [20]. Here we report the preclinical profile of DC-806, as well as the final phase 1c results, in which patients with psoriasis received twice daily (BID) doses of DC-806 for a total of 4 weeks. The phase 1a and phase 1b trial safety and PK characterization results will be presented elsewhere.

## Methods

For greater detail on the methodology and statistical analysis, the phase 1c study protocol and statistical analysis plan are available on request from the corresponding author.

### Consent for publication

All the results presented in this article are in aggregate form and no personally identifiable information was used for this study.

### Preclinical data

#### Determination of DC-806 cellular potency.

To determine cellular potency, recombinant human (h)IL-17AA (Genscript cat# Z03228), hIL-17AF (R&D cat# 5837-IL-010), hIL-17FF (R&D Systems 1335-IL), rat IL-17AA (R&D systems 8410-IL-025), and rat IL-17AF (R&D systems 9340-IL-025) were used for *in vitro* stimulation at the concentrations listed in **S1 Table**. Additionally, we prepared conditioned media from human TH17 cells as described previously [21], and used this supernatant at a dilution where the total IL-17 bioactivity was roughly equivalent to 1 ng/ml recombinant hIL-17AA. To control for IL-17FF bioactivity, an anti-IL-17F antibody that was empirically determined to only inhibit hIL-17FF, but not hIL-17AF, was used at 10 μg/ml (R&D MAB13352).

Human embryonic kidney (HEK)-Blue cells (InvivoGen hkb-il17) were cultured and stimulated as per manufacturer’s instructions. Human oral keratinocytes (ScienCell #2610), normal human epithelial keratinocytes (ATCC −200–011), immortalized human keratinocytes (HaCaT, Elabscience EP-CL-0090), human dermal fibroblasts (Sigma 106-05A), and murine NIH-3T3 fibroblasts (ATCC CRL-1658) were cultured as per the instructions of the manufacturer.

For potency determination, serial dilutions of the compound were prepared in dimethylsulfoxide (DMSO) such that final DMSO concentration in the assay was 0.1%. Cells and stimulant were added simultaneously and incubated for 18–22 hours. Secretory alkaline phosphatase (SEAP) signal was determined using QuantiBlue^TM^ reagent (InvivoGen REPQBS3), mouse IL-6 by HTRF (Cisbio 62MIL06PEG), hIL-6 by TR-FRET (PerkinElmer TRF1223C), and hCXCL-1 by TR-FRET (PerkinElmer TRF1279C). Curve fits and calculation of IC_50_ were performed either using GraphPad Prism (version 9.5.1) or Dotmatics software version 2020.1-108191-s.

#### Determination of DC-806 binding constant.

Binding to biotinylated hIL-17AA (Acro #ILA-H82Q1) or hIL-17AF (R&D Systems cat #5194-IL-025/CF) was determined by SPR, using a GE Biacore S200 instrument with a streptavidin capture chip.

#### Rat collagen-induced arthritis (CIA).

Rat collagen-induced arthritis (CIA) was performed by Inotiv (Boulder, CO) as described previously [22] and was approved and overseen by the Institutional Animal Care and Use Committee of Inotiv. Briefly, groups of 8 female Lewis rats were injected intradermally with porcine type II collagen on Days 0 and 7 to induce arthritis. The rats were dosed orally (PO), BID on Days 11–16 with vehicle (80% propylene glycol [PG]/20% D-α-tocopheryl polyethylene glycol succinate [vitamin E TPGS] in 50 mM sodium citrate buffer, pH 4.5) or DC-806 (50 or 100 mg/kg). Positive control rats were dosed PO, once daily (QD) with dexamethasone (0.075 mg/kg) or with a single subcutaneous (SC) injection of anti-IL-17A (20 mg/kg, XAB4, Creative Biolabs) administered on Day 11. On Day 17, all animals were euthanized for necropsy. Efficacy evaluation was based on animal body weights, daily ankle caliper measurements, ankle diameter expressed as area under the curve, terminal hind paw weights, and histopathologic evaluation of hind paws/ankles and knees. Plasma concentrations of DC-806 were determined 1 and 12 hours post dose on Days 11 and 16. Mean values for DC-806 dose groups were compared with the control group (no treatment) and animals treated with dexamethasone using analysis of variance (ANOVA; GraphPad Prism, version 9.5.1).

### Clinical data

#### Phase 1c study design and patients.

This phase 1c, randomized, double-blind, placebo-controlled clinical trial was designed to evaluate the safety, tolerability, PK, and clinical activity of DC-806 in adults with plaque psoriasis.

The clinical trial was conducted at the Medicines Evaluation Unit in Manchester, United Kingdom. The study protocol, amendments, and informed consent form were reviewed and approved by the North West – Greater Manchester Central Research Ethics Committee in accordance with the International Council for Harmonisation Guideline for Good Clinical Practice (ICH-GCP). Informed consent was obtained in writing from each patient before enrollment and signatures were witnessed by study site personnel. The study was conducted in accordance with ICH-GCP, the Declaration of Helsinki, and all relevant national laws. The study was registered with ClinicalTrials.gov (NCT06808815) and the EudraCT database (2021-002888-21).

The first patient consent was obtained on 20 January 2022, and the first patient was randomized 10 February 2022. The last patient was consented on 8 July 2022, and the last patient was randomized 14 July 2022. The last patient visit was on 23 August 2022.

**Fig 1:**
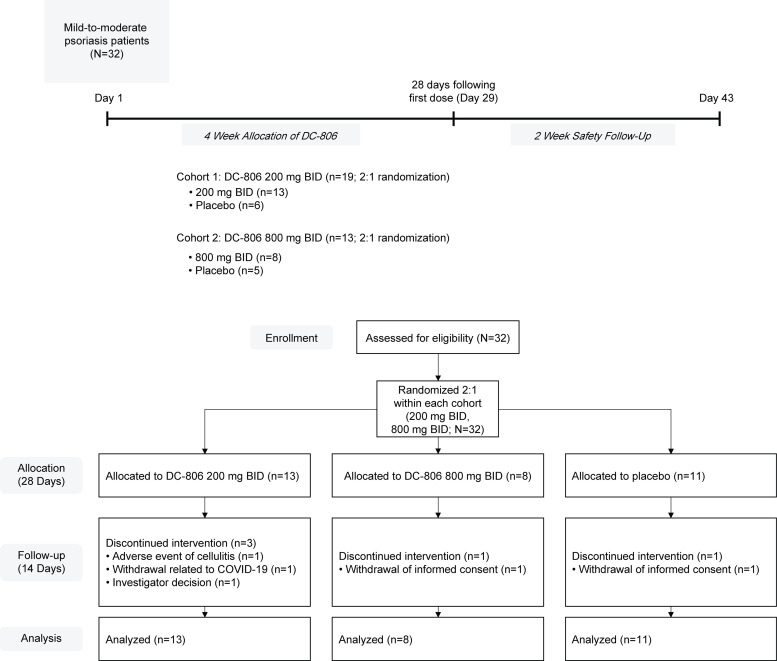
Phase 1c study design and CONSORT diagram. Study design timeline and CONSORT diagram. Patients enrolled at each dose level (200 mg BID or 800 mg BID) of the clinical trial and potential reasons for discontinuation. A total of 32 patients were treated, 27 of whom completed the 4-week trial period and 2-week safety follow-up. **Abbreviations:** BID, twice daily.

In the phase 1c trial, enrolled patients with psoriasis were randomized in a 2:1 ratio to receive DC-806 (800 mg BID or 200 mg BID) or placebo for 28 days (**Fig 1**). Eligible patients were male and female individuals aged 18–65 years (inclusive) with a documented diagnosis of plaque psoriasis for ≥6 months, physician global assessment (PGA) score of 2 or 3 (i.e., mild or moderate plaque psoriasis), body surface area (BSA) involvement ≥3%, and a minimum of 2 psoriatic lesions of at least 2 cm × 2 cm with at least 1 plaque in a site suitable for biopsy. Eligible patients also had a BMI ≥ 18 and ≤36 kg/m^2^ and were in good health as determined by medical history, physical examination, vital signs, 12-lead electrocardiogram (ECG), and clinical laboratory assessments at the time of screening, as judged by the Investigator or designee.

Patients were excluded if they had other skin conditions that would interfere with psoriasis evaluation as judged by the Investigator: a diagnosis of non-plaque psoriasis, plaque psoriasis restricted to the scalp, palms, soles, and face; or pustular, erythrodermic, inverse, or guttate psoriasis. Additionally, patients were ineligible if they had a diagnosis of psoriatic arthritis, uveitis, inflammatory bowel disease (IBD), or other immune-mediated conditions for which the patient required a current systemic immunosuppressant medical treatment. Finally, patients were not enrolled if there was the presence of active suicidal ideation using Columbia–Suicide Severity Rating Scale (C-SSRS) criteria, or if there was a history or presence of any clinically relevant acute or chronic medical or psychiatric condition that would interfere with the patient’s safety, as judged by the Investigator.

#### Randomization and masking.

Patients who completed the study screening assessments and met all the eligibility criteria were assigned a unique randomization number, allocated sequentially by a central site, before the first dose and received the corresponding investigational medicinal product (IMP) according to a randomization scheme generated by Veristat (https://www.veristat.com/). Specifically, 32 patients with psoriasis were randomized within each of two cohorts (200 mg BID x 28 Days, 800 mg BID x 28 Days) in a 2:1 ratio to receive DC-806 or placebo for 28 days.

Investigators, patients, and all study staff with direct patient contact were blinded to assignment to DC-806 or placebo. A designated unblinded pharmacist at the investigational site prepared the IMP bottles for dispensing to the site staff and patients. A patient’s treatment assignment was to be unblinded only when knowledge of the assignment was essential for the further clinical management of the patient on this study or might impact the safety of patients currently enrolled or patients in subsequent enrollment. Corresponding placebo tablets consisting of the same excipients and identical in appearance to the active tablets were provided. No changes to analyses were made after study unblinding.

### Study procedures

In total, 32 patients with psoriasis were randomized as described above. Patients fasted 2 hours before and 1 hour after each dose, took doses at home when not attending the site, and completed a daily dosing diary during periods of home dosing. Site staff completed study drug accountability and assessed compliance at each weekly visit. Medical history was summarized by the Medical Dictionary for Regulatory Activities, version 24.1 (MedDRA; https://www.meddra.org/) system organ class and preferred term and prior and concomitant medication by the WHODrug Dictionary, version September 2021 (https://who-umc.org/whodrug/whodrug-global/).

Patients underwent safety and tolerability assessments (adverse event [AE] monitoring, clinical laboratory tests, vital signs, 12-lead ECG, cardiac Holter monitoring, physical examination) and PK sampling at specified time points until discharge on Day 2.

### Changes in planned analyses and protocol deviations

Information on photography of lesion sites was reported but not analyzed as an endpoint. Analysis of a 50% reduction in the psoriasis area and severity index (PASI), a 75% reduction in PASI (PASI75), a 90% reduction in PASI (PASI90), and a 100% reduction in PASI (PASI100) was added in the statistical analysis plan.

A total of 18 protocol deviations in 7 (22%) patients were classified as important (**S2 Table**). For deviations categorized as assessment/procedure performed outside the permitted time window, 2 in the active treatment group were related to COVID-19 and 2 in the placebo group were related to patient work commitments.

### Outcomes

The primary objective of this phase 1c trial was to assess the safety and tolerability of multiple oral doses of DC-806 in patients with psoriasis after 4 weeks of treatment. Safety data were analyzed in the Safety Analysis Set (all patients who received at least 1 dose of IMP, analyzed according to the initial treatment and dose actually taken). AE verbatim terms were coded and categorized according to the MedDRA, version 24.1.

A treatment-emergent AE (TEAE) was defined as an AE that started on or after the start of the administration of IMP but not 7 or more days after the last administration of IMP. A mild AE was defined as an event that was easily tolerated by the patient, causing minimal discomfort, and not interfering with everyday activities. A moderate AE was an event that caused sufficient discomfort and interfered with normal everyday activities, and a severe AE was an event that prevented normal everyday activities. Serious AEs (SAEs) were defined as an AE that, at any dose, resulted in death, was life-threatening, required inpatient hospitalization or prolongation of existing hospitalization, resulted in persistent incapacity, or was a congenital anomaly or birth defect. AEs of special interest were those that have been reported with antibody inhibition of IL-17 signaling and included new onset of IBD (ulcerative colitis or Crohn’s disease) and suicidal ideation [23].

The incidence proportion of TEAEs, serious TEAEs and TEAEs leading to withdrawal were summarized by system organ class and preferred term. TEAEs were also summarized by severity and relationship to IMP. Causality of each AE to the IMP was assessed as unlikely related, possibly related, or probably related. If a causal relationship between the study IMP and the AE was not a reasonable possibility, they were unlikely related. For an event that had a suggestive temporal relationship to the study IMP, and an alternative etiology was equally or less likely, the causality was possibly related. Finally, if the event had a strong temporal relationship to the study IMP or recurred on re-challenge, and another etiology was unlikely or significantly unlikely, the causality was deemed probably related.

Clinical laboratory, vital signs, cardiac Holter monitoring, and ECG parameters were described using summary statistics. Physical examination timings and C-SSRS responses were listed.

The secondary objective of this phase 1c trial was to evaluate systemic PK of DC-806 after multiple oral doses in patients with psoriasis. The PK Concentration Set included all patients who received at least 1 dose of active drug, and who had at least 1 post-dose plasma PK concentration recorded and no significant deviation or AE that may affect PK concentrations. The PK Parameter Analysis Set included all patients who were randomized and received at least 1 dose of active drug, with no significant deviation or AE that may affect PK parameters and had sufficient data for at least 1 PK parameter to be determined. Concentrations and PK parameters were summarized in the PK Concentration Set and PK Parameter Analysis Set, respectively. The lower limit of quantification (LLQ) for DC-806 quantification was 1 ng/mL, and values <LLQ were set to 0.5 × LLQ for calculation of summary plasma concentration statistics. Dose proportionality was assessed for PK parameters.

As an exploratory objective, the clinical activity of DC-806 in patients with psoriasis was analyzed. PASI was analyzed in all but 5 patients who discontinued dosing before Day 29 and thus had an early termination visit rather than a Day 29 visit. Dermatology assessments were conducted at screening, baseline, Days 8, 15, 22, 29 (end of treatment), and follow-up or early termination visit. PASI was summarized as the number (and percentage) of those achieving response at Day 29, together with the percentage change from baseline to Day 29.

### Measurement of pharmacodynamic (PD) biomarker level

The pharmacodynamic (PD) Analysis Set included patients who received at least 1 dose of DC-806 or placebo and had at least 1 pre-dose and one post-dose evaluable measurement of any of the evaluable PD biomarker levels. PD biomarker levels were measured following manufacturer’s protocol of commercially available enzyme-linked immunosorbent assay (ELISA) kits. Serum IL-17A level was measured using Quantikine^®^ High Sensitivity (HS) Human IL-17 Immunoassay kit #HS170 (R&D Systems), which mostly detects IL-17AA with approximately 3% cross-reactivity to IL-17AF [24]. As such, we assumed that serum IL-17AA constituted most, if not all, of the serum IL-17A levels measured. Serum beta defensin-2 (BD-2) level was measured using Human beta-defensin 2 ELISA kit #100–250-BD2 (Alpha Diagnostic International) and IL-19 level in plasma was measured using Human IL-19 ELISA kit #ab231922 (Abcam). Serum biomarker assessments of IL-17A and BD-2 were conducted at screening, baseline, and Days 1, 2, 8, 15, 22, 29, and 43. Plasma biomarker assessments of IL-19 were conducted at Days 1, 2, 8, 15, 22, 29, and 43. On any of the dosing days, PD samples were collected before the morning dose was administered. On study Days 1 and 22, PD samples were also collected 2 hours and 6 hours post-morning dose. IL-19 was detectable in all subjects at baseline. For those post-treatment samples that went below the LLQ, a value of 3.9 pg/mL, half of the LLQ value, was assigned for calculation of percentage change from baseline. Biomarker measurements were summarized as the change in mean (standard error of the mean [SEM]) value from baseline through Study Day 29.

### Measurement of plasma DC-806 concentrations

Plasma samples were analyzed using a validated high-performance liquid chromatography with tandem mass spectrometry (LC-MS/MS) method. The LLQ of the assay is 1 ng/mL DC-806.

### Statistical analysis

The sample size for this study was selected based on clinical considerations of phase 1 studies. Due to the exploratory nature of this study, no formal power or hypothesis testing was considered to determine sample size. A sample size of up to 21 patients (up to 14 active and up to 7 placebo) per cohort was determined to be adequate to meet the study objectives.

Baseline was defined as the last non-missing measurement before the administration of the first dose of IMP and baseline dermatological assessments were taken 1–5 days prior to first dose of IMP. Missing data were not imputed. For patients who discontinued the study early, all available data were included in analyses and outputs. Statistical analysis was descriptive and exploratory. Continuous data were summarized using descriptive statistics (n, mean, standard deviation, median, minimum, and maximum) and categorical data were summarized using the number and percentage of patients in each category.

For outcomes assessing clinical activity (PASI), results and change from baseline were summarized by treatment and time point. An analysis of covariance (ANCOVA) model was fitted for each endpoint with the endpoint value at Day 29 as the dependent variable, treatment group as a fixed effect, and baseline value as a covariate. Adjusted least square (LS) means for the treatments estimated by the model were calculated with the associated standard errors (SE) and 95% confidence intervals (CI). The adjusted LS mean, SE, 95% CI, and 2-sided *P* value of the treatment difference were also presented. The adjusted percentage change from baseline for each treatment group was derived from the adjusted LS Mean, along with the 95% CI as 100 × (adjusted treatment mean at Day 29 – global mean at baseline)/global mean at baseline. The global mean was the mean at baseline of all patients included in the analysis in the 3 treatment groups. To understand how the baseline disease severity may have affected the treatment benefit, *post hoc* subgroup analyses of patients with baseline PASI <6 and patients ≥6 were performed in patients who had PASI available at baseline and the timepoint of interest.

All planned statistical analyses were performed and output prepared using SAS Version 9.4.

## Results

### Preclinical testing and pharmacological effect validation of DC-806

**Fig 2:**
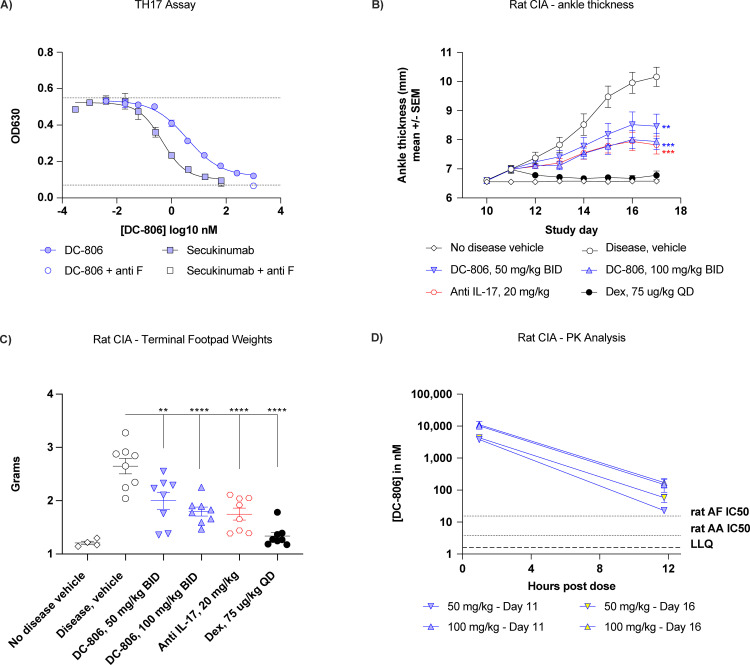
Preclinical evaluation of IL-17 inhibition by small molecule DC-806. **(A)** Inhibition of IL-17 bioactivity as secreted by human TH17 cells and assayed by a reporter cell assay. Dotted lines represent positive and negative assay controls. 1 representative of 7 experiments with different donors is shown. The error bars represent the SEM. **(B, C)** Evaluation of DC-806 in rat CIA. Rats were randomized on Day 11 and dosed as indicated. Daily measurements of ankle thickness (B) and terminal measurement of footpad weights (C) were used as efficacy readouts. **(D)** Preclinical evaluation of DC-806 serum levels. PK sampling for exposure determination was performed on Days 11, 16, at 1 and 12 hours post dose. The dotted lines represent the uncorrected IC_50_ for rat IL-17AA and rat IL-17AF as listed in S1 Table. The dashed line represents the LLQ of the quantification assay. All error bars represent the SEM. *P < 0.05; **P < 0.01; ***P < 0.001; ****P < 0.0001. **Abbreviations**: anti-F, anti-IL-17F; BID, twice daily; CIA, collagen-induced arthritis; Dex, dexamethasone; IC_50_, half-maximal inhibitory concentration; IL-17, interleukin-17; LLQ, lower limit of quantification; PK, pharmacokinetic; QD, once daily; SEM, standard error of mean; TH17, T-helper 17.

Initial chemical lead molecules were identified from targeted DELSCAPE DNA-encoded libraries, and an innovative medicinal chemistry optimization campaign led to the identification of DC-806. DC-806 has a molecular weight of 625.8 g/mol and a chemical structure shown in **S1 Fig**. Surface plasmon resonance (SPR) results demonstrated that DC-806 binds with high affinity to IL-17AA, and with somewhat reduced affinity to IL-17AF (**S1 Table**). Similarly, DC-806 neutralized the bioactivity of recombinant IL-17AA and IL-17AF, but not IL-17FF, in several cell-based assays (**S1 Table**). To determine the capacity of DC-806 to neutralize primary human cell-derived IL-17 bioactivity, human TH17 cell-conditioned media was incubated with an IL-17 reporter cell line in the presence of increasing concentrations of DC-806. As in the recombinant cell lines, DC-806 neutralized IL-17 bioactivity from human TH17 cells with an average potency (measured via half-maximal inhibitory concentration [IC_50_]) of 5.7 nM across 7 donors. In this model, DC-806 demonstrated the same maximal inhibition as secukinumab (**Fig 2A**). A small amount of bioactivity stems from IL-17FF, which is not neutralized by either secukinumab or DC-806, but can be revealed through the addition of a neutralizing antibody against IL-17FF.

The potency of DC-806 in cellular assays was proportional to the amount of IL-17 used for stimulation; higher amounts of IL-17 used for stimulation resulted in a higher IC_50_ for DC-806 (**S1 Table**). As *in vivo* concentrations of IL-17 reported in specific human disease settings are reported to be more than 50 fold lower than the concentrations used in reporter assays [25–28], we hypothesized that full inhibition of IL-17 bioactivity *in vivo* would be achieved at lower exposure levels than estimated based on the potency of DC-806 in *in vitro* cell-based assays.

To determine what exposure of DC-806 would provide a therapeutic effect comparable to antibody-mediated IL-17A neutralization, we first used a rat model of collagen-induced arthritis (CIA; **Fig 2B-2D**). Here, DC-806 dosed at 100 mg/kg BID reduced ankle thickness and terminal footpad weight with similar efficacy to that of an anti-rat IL-17A antibody (XAB4, dosed once at 20 mg/kg). Under the conservative assumption that target coverage at pre-dose trough plasma levels drives efficacy, we reasoned that a human dose covering approximately 27-fold IC_50_ at steady state trough could provide maximal efficacy, and that a lower dose, covering 3-fold the IC_50_ would produce a significant, but submaximal effect.

### Phase 1c study patient disposition and baseline characteristics

In a single ascending dose and multiple ascending dose portion of the trial (phase 1a and 1b), DC-806 demonstrated favorable PK and safety and tolerability profiles in healthy volunteers at all doses tested up to 800 mg BID for 7 days. Subsequently, we proceeded with a phase 1c (NCT06808815) randomized, placebo-controlled trial of DC-806 dosed at 200 mg BID and 800 mg BID in patients with mild-to-moderate psoriasis for 28 days (**Fig 1**). The primary and secondary endpoints assessed the safety and tolerability and PK profile of DC-806, respectively. Additional exploratory outcomes included the PD and clinical activity of DC-806. Inclusion and exclusion criteria are detailed in the **Methods** section of this manuscript.

From January 2022 to July 2022, a total of 32 patients consented and were randomized within each cohort (200 mg BID, 800 mg BID) in a 2:1 ratio to receive DC-806 or placebo for 28 days (**Fig 1**). In total, 13 patients received DC-806 200 mg BID, 8 patients received DC-806 800 mg BID, and 11 patients received placebo. Five (16%) patients discontinued from the study for the following reasons: withdrew consent due to worsening psoriasis (n = 1); withdrew consent (n = 1); experienced an AE of cellulitis (n = 1); experienced asymptomatic COVID-19 infection (n = 1); or withdrew due to Investigator decision (n = 1). Despite the early discontinuations, all patients (N = 32) were included in the Full Analysis Set and Safety Analysis Set. The PK Concentration Set included all patients who received at least 1 dose of DC-806 and who had at least 1 post-dose plasma PK concentration recorded (n = 21). The PASI Analysis Set included all but the 5 patients who discontinued dosing before Day 29 and thus had an early termination visit rather than a Day 29 visit (n = 27). For the PD biomarker analysis, patients who received at least 1 dose of DC-806 or placebo and had at least 1 pre-dose and 1 post-dose evaluable measurement of the biomarker of interest were included (analysis set varied by day and biomarker). In a *post hoc* subgroup analysis conducted to understand how baseline disease severity may have affected the treatment benefit, patients who had PASI measurements available at both baseline and at time points of interest were included (n = 27). Protocol deviations classified as important are reported in **S2 Table**.

Baseline demographic characteristics were evenly distributed between the 200 mg BID, 800 mg BID, and placebo groups (**Table 1**). For the phase 1c population overall, the median (range) age was 43 (19, 61) years and most (81.3%) patients were male. Medical and surgical history, including prior and concomitant medication use, were similar among the groups and reflected the eligibility criteria.

**Table 1:** Phase 1c cohort patients’ baseline characteristics.

Patients, n (%)	DC-806			Placebo n = 11	Total N = 32
	200 mg BID n = 13	800 mg BID n = 8	Total n = 21		
Male sex, n (%)*	9 (69)	8 (100)	17 (81)	9 (82)	26 (81)
Age, years, median (range)*	44 (19, 60)	52 (19, 61)	45 (19, 61)	39 (19, 54)	43 (19, 61)
Race, n (%)*					
Asian	3 (23)	0	3 (14)	0	3 (9)
White	10 (77)	8 (100)	18 (86)	11 (100)	29 (91)
Ethnicity, n (%)*					
Not Hispanic/Latino	13 (100)	8 (100)	21 (100)	11 (100)	32 (100)
Body mass index, kg/m^2^, median (range)*	30.0 (23.1, 33.9)	27.2 (19.1, 35.9)	29.1 (19.1, 35.9)	28.4 (19.5, 35.9)	29.0 (19.1, 35.9)
PASI, median (range)**	5.2 (2.9, 13.9)	6.5 (4.1, 11.2)	5.6 (2.9, 13.9)	6.8 (4.3, 10.2)	6.2 (2.9, 13.9)
Percent BSA involvement, median (range)**	5.5 (3.1, 19.0)	6.7 (3.6, 25.4)	6.1 (3.1, 25.4)	7.9 (3.6, 16.6)	6.1(3.1, 25.4)
PGA score, median (range)**	3.0 (2.0, 3.0)	3.0 (2.0, 3.0)	3.0 (2.0, 3.0)	3.0 (2.0, 3.0)	3.0 (2.0, 3.0)

Summary of baseline characteristics and baseline values of clinical activity in patients with psoriasis by dose group. *Safety Analysis Set; **Full Analysis Set. **Abbreviations:** BID, twice daily; BSA, body surface area; PASI, psoriasis area and severity index; PGA, physician global assessment.

Baseline disease characteristics are summarized in **Table 1** and were consistent with the inclusion criterion that all patients have mild or moderate plaque psoriasis at baseline. However, imbalances between groups were seen in total PASI and total percent BSA involvement. Median (range) PASI was lower in the 200 mg BID group at 5.2 (2.9, 13.9) compared to both the 800 mg BID group (6.5 [4.1, 11.2]) and placebo group (6.8 [4.3, 10.2]). Likewise, the median (range) percent BSA involvement was lower in the 200 mg BID group at 5.5 (3.1, 19.0) than in the 800 mg BID group (6.7 [3.6, 25.4]) and placebo group (7.9 [3.6, 16.6]). These median values therefore suggest that patients in the 200 mg BID group had lower disease burden compared with the placebo group and 800 mg BID group.

### DC-806 treatment appeared safe and well tolerated

The primary objective of the phase 1c trial was to assess the safety and tolerability of multiple oral doses of DC-806 in patients with psoriasis after 4 weeks of treatment. As was observed in the phase 1a/b trial of healthy volunteers, in the phase 1c trial of patients with psoriasis, DC-806 appeared safe and well tolerated with BID dosing of 200 mg or 800 mg for 28 days. No consistent, dose-dependent effect or clinically meaningful differences in safety or tolerability were observed between the DC-806 treatment groups and the placebo group.

No severe AEs, SAEs, AEs leading to death, AEs of special interest, or treatment-related AEs (TRAEs) that led to discontinuation were reported (**Table 2**). In both the active treatment and placebo groups, all AEs were mild or moderate in severity. Among patients who received DC-806, the only TEAEs reported by more than 1 patient were headache in 6 (29%) patients, abdominal discomfort in 2 (10%) patients, COVID-19 or asymptomatic COVID-19 in 3 (14%) patients, and skin abrasion in 2 (10%) of patients. These TEAEs were considered unlikely to be related to DC-806, except for 1 event of mild abdominal discomfort and nausea with onset on Day 1 in a patient in the 800 mg BID group. No safety signals were seen in clinical laboratory results, vital signs, physical examination findings, ECG parameters or interpretations, or cardiac Holter monitoring data.

**Table 2:** Safety and tolerability profile in the phase 1c cohort.

Patients, n (%)	DC-806			Placebo n = 11
	200 mg BID n = 13	800 mg BID n = 8	Total n = 21	
AEs	10 (76.9)	6 (75.0)	16 (76.2)	4 (36.4)
Mild	7 (53.8)	6 (75.0)	13 (61.9)	1 (9.1)
Moderate	3 (23.1)	0	3 (14.3)	3 (27.3)
Severe	0	0	0	0
Related AEs	0	1 (12.5)	1 (4.8)	0
SAEs	0	0	0	0
Serious TRAEs	0	0	0	0
AEs leading to discontinuation of study treatment	3 (23.1)	0	3 (14.3)	1 (9.1)
TRAEs leading to discontinuation of study treatment	0	0	0	0
Types of TEAEs occurring in ≥2 patients				
Headache	4 (30.8)	2 (25.0)	6 (28.6)	1 (9.1)
Abdominal discomfort	1 (7.7)	1 (12.5)	2 (9.5)	0
COVID-19	2 (15.4)	1 (12.5)	3 (14.3)	0
Tonsilitis	0	0	0	2 (18.2)
Skin abrasion	2 (15.4)	0	2 (9.5)	0

AEs possibly related to DC-806 200 mg BID or 800 mg BID. Safety Analysis Set. AEs are defined in the methodology. **Abbreviations:** AE, adverse event; BID, twice daily; SAE, serious adverse event; TEAE, treatment-emergent adverse event; TRAE, treatment-related adverse event.

### DC-806 achieved pharmacologically active steady state concentrations in psoriasis patients

**Fig 3:**
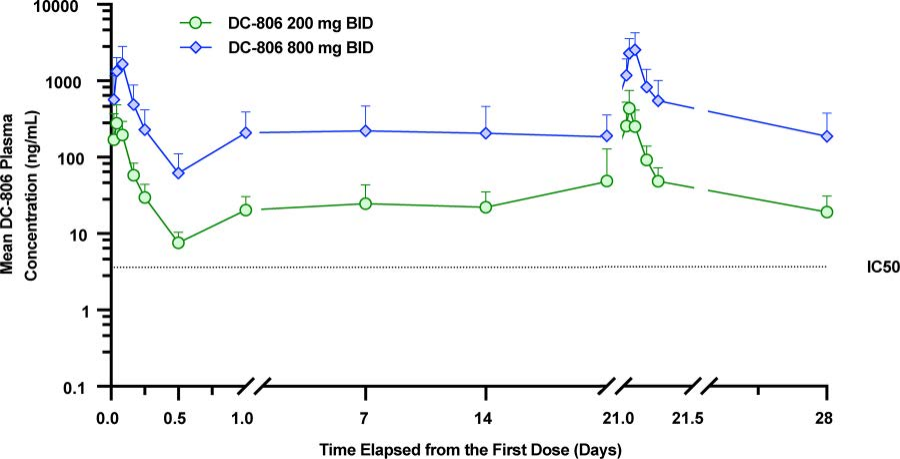
Phase 1c PK profile of DC-806 through 28 days after the first dose. Graph detailing the group mean ± SD DC-806 plasma concentration by 200 mg BID (green circle) and 800 mg BID (blue diamond) groups on a semilogarithmic scale through Study Day 29. PK in psoriasis patients was comparable to healthy volunteer PK, with consistent trough levels over 28 days after achieving steady state around Day 3. The dotted line represents IC_50_ for recombinant human IL-17AA, as obtained using HEK-blue IL-17 cell-based assay (S1 Table). PK Concentration Analysis Set. **Abbreviations**: BID, twice daily; HEK: human embryonic kidney; IC_50_, half-maximal inhibitory concentration; IL-17, interleukin-17; PK, pharmacokinetic; SD, standard deviation.

The secondary objective of the phase 1c trial was to evaluate the systemic PK of DC-806 after BID oral doses in psoriasis patients. The DC-806 PK profile was characterized in the phase 1a/b trial in healthy volunteers. In particular, the phase 1a/b preliminary PK model predicted that 200 mg and 800 mg BID would achieve steady state trough concentrations (C_trough,ss_) at levels>IC_50_ and>10x IC_50_, respectively. As predicted, in the phase 1c trial of psoriatic patients, steady state exposure was achieved within the first week of dosing where the mean C_trough,ss_ values were generally maintained at levels of approximately 5- to 6-fold IC_50_ in the 200 mg BID group and approximately ~51- to 61-fold IC_50_ in the 800 mg BID group (**Fig 3**).

### DC-806 achieved clinical improvements in psoriasis after treatment for 28 days

**Fig 4:**
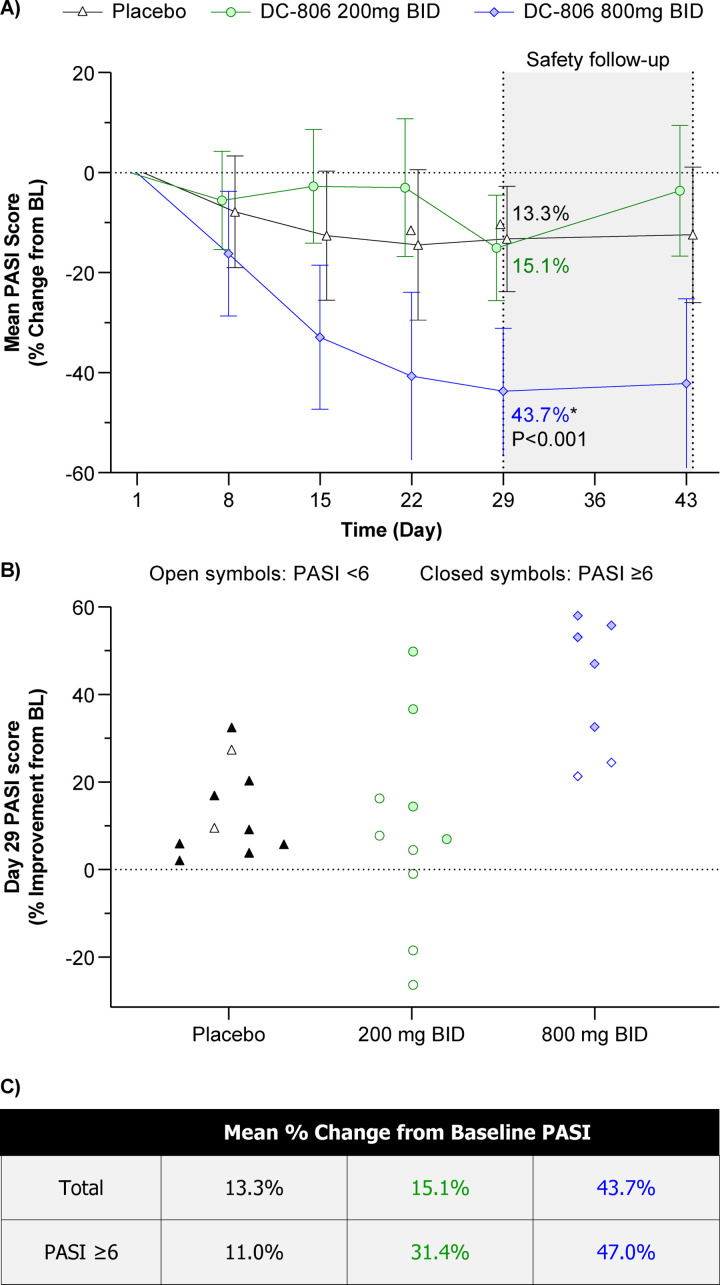
Reductions in PASI during and after 28 days of DC-806 treatment in the phase 1c cohort. **(A–C)** DC-806 demonstrated clear benefits with reductions in PASI compared to placebo. Blue diamonds and text represent the 800 mg BID group; Green circles and text represent the 200 mg BID group; Black triangles and text represent the placebo group. Dotted line represents baseline PASI evaluation. **(A)** Adjusted mean ± 95% CI change from baseline in total PASI through Day 43. *DC-806 800 mg BID vs placebo *P* = 0.0008. *P* value was calculated by ANCOVA. *P* values are two-sided. **(B)** Results from the *post hoc* subgroup analysis demonstrating percentage improvement in PASI from baseline to Day 29 in patients with known PASI ≥6 (solid symbols; n = 17) and PASI <6 (open symbols; n = 10) at baseline. **(C)** Mean percentage change in PASI from baseline to Day 29 in both the primary analysis (Total) and in those with PASI ≥6. PASI Analysis Set (**A**,**B**) and Post Hoc Analysis Set **(C)**. **Abbreviations:** ANCOVA, analysis of covariance; BID, twice daily; BL, baseline; CI, confidence interval; PASI, psoriasis area and severity index.

In an exploratory analysis of clinical activity, PASI showed a significantly greater reduction from baseline to Day 29 in the 800 mg BID group than in the placebo group (adjusted mean reduction of 43.7% vs 13.3%; *P* = 0.0008; **Fig 4A**; **S3 Table**). The reduction in PASI from baseline was less pronounced in the 200 mg BID group (15.1%) than in the 800 mg BID group. No patients in the placebo group, 1 (10%) patient in the 200 mg BID group, and 3 (43%) patients in the 800 mg BID group had ≥ 50% reduction from baseline to Day 29.

**Fig 5:**
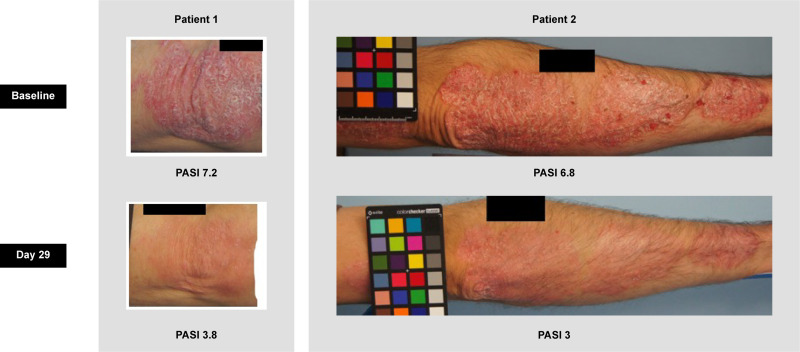
Visual improvements in psoriasis after 28 days in patients receiving DC-806 800 mg BID. Close-up photographs of the severe lesions of two patients who received DC-806 800 mg BID taken at baseline and Day 29 and the corresponding PASI evaluation. **Abbreviations**: BID, twice daily; PASI, psoriasis area and severity index.

Of those with PASI measurements at both baseline and Day 29, more patients in the 200 mg BID group (6/10; 60%) had PASI <6 than those in the 800 mg BID group (2/7; 29%) at baseline. To understand how baseline severity affected treatment benefit, a *post hoc* subgroup analysis of patients with PASI ≥6 (n = 17) and PASI <6 (n = 10) at baseline was performed (**Fig 4B**). Across both DC-806 treatment groups, numerically larger reductions in PASI from baseline to Day 29 were observed in patients with baseline PASI ≥6 compared with patients with baseline PASI <6. Among patients with baseline PASI ≥6, the adjusted mean percentage reduction in PASI was numerically higher in both the 200 mg BID group (31.4%) and the 800 mg BID group (47.0%) than in the placebo group (11.0%; **Fig 4C**). In 2 patients who received 800 mg BID, visual lesion severity scores also indicated significant improvement in lesions at Day 29 (**Fig 5**).

### DC-806 treatment affected established PD biomarkers of psoriasis

**Fig 6:**
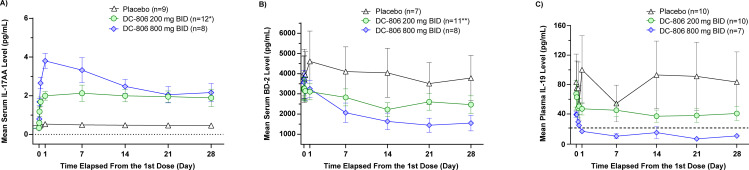
Biomarker responses during 28 days of DC-806 treatment in the phase 1c cohort. **(A–C)** DC-806 demonstrated biological effects as evidenced by dose-dependent responses of biomarkers of interest compared to placebo. Blue diamonds represent the 800 mg BID group; Green circles represent the 200 mg BID group; Black triangles represent the placebo group. **(A)** Change in mean ± SEM serum IL-17AA levels from time of first dose through 28 days post first dose. The dotted line represents low limit of quantification of the assay. **(B)** Change in mean ± SEM serum BD-2 levels from time of first dose through 28 days thereafter. *Except on Day 28 when n = 10; **Except on Day 28 when n = 9. **(C)** Change in mean ± SEM plasma IL-19 levels from time of first dose through 28 days post first dose. The dotted line represents normalization value of 21 pg/mL. PD Biomarker Analysis Set. **Abbreviations:** BD-2, beta defensin-2; BID, twice daily; BL, baseline; IL-17, interleukin-17; PASI, psoriasis area and severity index; PD, pharmacodynamic; SEM, standard error of mean.

The PASI outcomes were reflected in dose-dependent responses of biomarkers of interest, including serum IL-17AA and BD-2, and plasma IL-19. Across all three biomarker analyses, DC-806 demonstrated greater inhibition of IL-17 bioactivity in the 800 mg BID group than in the 200 mg BID group (**Fig 6**). No consistent changes in any of the three biomarkers were observed in patients treated with placebo.

Serum IL-17AA levels increased rapidly within 6 hours of the first DC-806 dose in both the 200 mg BID and 800 mg BID groups in a dose-dependent manner (**Fig 6A**). Following DC-806 treatment, mean (SEM) serum IL-17AA levels peaked 7 days after the first dose to 2.2 pg/mL (0.25–5.7) in the 200 mg BID group and 24 hours after first dose to 3.8 pg/mL (2.0–5.4) in the 800 mg BID dose group from corresponding baseline levels of 0.3 pg/mL (0.2–0.8) and 0.3 pg/mL (0.2–0.5), respectively. In the 200 mg BID dose group, the peak IL-17AA level was maintained throughout the 28-day treatment period. In the 800 mg BID dose group, IL-17AA levels reduced gradually from the peak level (observed on Day 2) to a mean level of approximately 2.0 pg/mL by Day 22, similar to the steady-state level observed in the 200 mg BID group.

DC-806 treatment resulted in a decrease in mean (SEM) serum BD-2 levels in both dosage groups in a dose-dependent manner (**Fig 6B**). A maximum reduction of approximately 18% (1257.5–5130.8 pg/mL) in the 200 mg BID dose group and 59% (293.9–3302.3 pg/mL) in the 800 mg BID dose group from corresponding baseline level (200 mg BID: 1666.4–6381.2 pg/mL; 800 mg BID: 2407.8–6144.2 pg/mL) was observed on Day 22. No further change in BD-2 levels was observed at the end of the 28-day treatment period.

DC-806 treatment resulted in a decrease in mean (SEM) plasma IL-19 levels during the 28 days of treatment (**Fig 6C**). A mean decrease of 28.7% (11.2–129.0 pg/ml) and 35.3% (20.2–46.7 pg/ml) in plasma IL-19 from respective baseline level (14.5–171.0 pg/ml for 200 mg BID and 23.4–55.8 pg/ml for 800 mg BID) was observed within 6 hours after the first dose in the 200 mg and 800 mg BID DC-806 groups, respectively. A maximum mean decrease in IL-19 level of 43.8% (10.4–86.8 pg/ml) in the 200 mg BID group and 86.6% (3.9–17.8 pg/ml) in the 800 mg BID group was observed.

## Discussion

IL-17 is a well-established target in psoriasis, with multiple biologic therapies providing impressive therapeutic benefit. While the importance ascribed to the route of treatment administration varies across psoriasis severity [29], studies of psoriasis treatment patterns, biologic and oral treatment switch rates, and healthcare resource utilization suggest a patient preference for oral treatment, particularly with mild or moderate disease [13,30]. As current oral psoriasis treatments are limited by their efficacy and tolerability when compared with biologic therapy [18,19], there remains a substantial unmet need for efficacious and well-tolerated oral therapies.

Meaningful disruption of cytokine/receptor interactions by orally bioavailable small molecule drugs has historically been difficult to achieve given the generally flat, featureless, and difficult-to-target protein-protein interfaces [31]. IL-17 dimers feature a central pocket that can accommodate small molecule inhibitors [32], and several attempts to develop such drugs have been made. However, the clinical success of such therapies is yet to be fully realized [33]. The phase 1 trial of LY3509754 in patients with psoriasis (NCT04152382) was discontinued due to safety concerns that are not related to IL-17 biology [34]. The phase 1 trial of the IL-17 inhibitor LEO 153339 in healthy adults has not yet read out (NCT04883333) [35]. Therefore, DC-806 is the first small molecule targeting the protein-protein interaction of IL-17 and its receptor that has demonstrated effective blockade of IL-17A activity with a favorable safety profile and dose-dependent pharmacological activities in a clinical study. By effectively disrupting the interaction of IL-17 with its receptor, DC-806 has the theoretical potential to provide antibody-like efficacy and safety in human patients.

*In vitro* and *in vivo* preclinical models consistently demonstrated that DC-806 has the potential to match the biological effect of anti-IL17 antibody therapeutics such as secukinumab, thereby supporting the progression of DC-806 into clinical development. The rat CIA model, a robust *in vivo* model of inflammation with a significant IL-17-driven component [36], was used to project human DC-806 target serum levels that were expected to result in substantial efficacy in patients. Consistent with our intention, DC-806 dosed at 800 mg BID in this trial, or at 100 mg/kg BID in rats, achieved similar C_trough,ss_, IL-17A target coverages of ~51–61-fold IC_50_, and ~20–44-fold IC_50_, respectively.

In the phase 1c clinical trial, DC-806 at 200 mg BID and 800 mg BID doses met the primary and secondary endpoints, reaching target trough plasma concentrations identified in the preclinical *in vivo* studies with a favorable safety and tolerability profile. In the active treatment arms, all TEAEs were classified as mild (>80%) or moderate, and no severe AEs or TRAEs leading to trial discontinuation were reported. Additionally, no SAEs or AEs of special interest were observed, including new onset of IBD (ulcerative colitis or Crohn’s disease) previously reported with antibodies that target IL-17 signaling [37], or suicidal ideation, previously reported with brodalumab [23]. Overall, these results to date suggest that DC-806 appears to be a well-tolerated oral drug.

Our preliminary phase 1c results demonstrated clinical efficacy at both 200 mg BID and 800 mg BID as evidenced by improvement in PASI. In the overall population, DC-806 demonstrated clear benefit in the 800 mg BID group with a significant reduction in PASI compared to placebo. While the 200 mg BID group did not separate from placebo, a *post hoc* subgroup analysis suggested that DC-806 had better PASI improvement in patients with higher baseline PASI (PASI ≥6) compared to placebo at this dosage (albeit this *post hoc* analysis is limited by small sample size).

DC-806 at both doses demonstrated promising IL-17 target engagement, affecting established PD biomarkers of IL-17 signaling such as IL-17A, BD-2, and IL-19 [26,38–41]. Since IL-17 is hypothesized to be cleared partially by receptor-mediated endocytosis upon receptor engagement and initiation of signaling [42], the use of any pharmacological agent that prevents binding of IL-17 to its receptor would also lead to an increase in IL-17 serum levels. However, this extra IL-17 would not be bioactive, as it is prevented from accessing the receptor by said pharmacological agent. To provide a relevant example, treatment of patients with brodalumab, an anti-IL-17 receptor A antibody that prevents binding of IL-17, led to an increase in IL-17AA serum levels while providing therapeutic efficacy [43]. DC-806 treatment resulted in a similar increase in serum IL-17AA levels as brodalumab, while decreasing PASI scores and serum levels of proximal downstream biomarkers IL-19 and BD-2 [26]. Together, these observations suggest that DC-806 prevents IL-17AA from binding to its receptor, resulting simultaneously in pathway inhibition and in increased serum levels of biologically inactive, DC-806-bound IL-17AA. Importantly, we did not observe disease rebound upon drug withdrawal in spite of the elevated IL-17AA serum levels in DC-806 treated patients. We postulate that the half-life of free IL-17 is much shorter than the half-life of DC-806, resulting in gradual disappearance of the excess IL-17AA as DC-806 exposure levels decrease.

The biomarker IL-19 was monitored because of its known correlation with disease severity and potential to predict long term clinical benefit [40,44]. In a phase 2 study of ixekizumab [40], an anti-IL-17A antibody, IL-19 reductions below 21 pg/mL (benchmarked as upper limits of normal for healthy volunteers) after 2 weeks of ixekizumab treatment were predictive of PASI90 or PASI100 responses at Week 16. In our study, DC-806 800 mg BID dose-dependently reduced IL-19 serum concentration to less than 21 pg/mL within 2 weeks and thus mimicked the effects of ixekizumab [40]. Our results therefore suggest potential efficacy of longer-term DC-806 treatment in psoriasis.

We acknowledge that definitive conclusions about efficacy and safety are not possible from a phase 1c study. This study is limited by small sample sizes and a short period of intervention, which could be improved upon in larger, more rigorous studies of DC-806. Although PASI scores were collected longitudinally, the prespecified exploratory efficacy endpoint was the PASI-75 response at Day 29, which supported the use of ANCOVA for the primary analysis. While mixed models for repeated measures (MMRM) can offer greater efficiency for modeling trajectories over time, its application in small early-phase studies is often limited by convergence and stability issues. Given the study’s exploratory design and single primary time point, ANCOVA provided a practical and appropriate approach. Future larger studies may incorporate MMRM to better characterize response patterns [45].

In addition, the study was conducted in a mild-to-moderate psoriasis population, because it would have been unethical to enroll patients with more severe disease considering the short treatment duration and availability of many other approved treatment options. Given the preliminary clinical data, combined with a favorable safety and tolerability profile, results support further development of DC-806 in psoriasis and other IL-17-mediated diseases, such as psoriatic arthritis, ankylosing spondylitis, and hidradenitis suppurativa. Furthermore, our results suggest that targeting other inflammatory cytokines via oral small molecules may also be feasible, opening up new therapeutic options for immunological diseases.

## Supporting information

S1 Table Cell-based and biophysical assay parameters of DC-806. *The supernatant from each donor was used at a dilution that corresponded to the bioactivity of 1 ng/mL recombinant human IL-17A. **Abbreviations**: CXCL-1, chemokine (CXC motif) ligand 1; IL, interleukin; HEK: human embryonic kidney; K_D_, dissociation constant; mIL, murine interleukin; nM, nanomolar; SEAP, secretory alkaline phosphatase; SPR, surface plasmon resonance; TH17, T-helper 17.
(DOCX)

S2 Table Important protocol deviations by dose group. A total of 18 protocol deviations in 7 (22%) were classified as important. In the deviations categorized as assessment/procedure performed outside of permitted time window, 2 in the DC-806 group were related to COVID-19 and 2 in the placebo group were related to patient work commitments. **Abbreviations:** BID, twice daily.
(DOCX)

S3 Table Analysis of percentage change from baseline for the psoriasis area and severity index (PASI) score for total body at Day 29. N = the number of subjects in the analysis set. n is the number of subjects who have results at both baseline and Day 29. (%) = n/N*100. Baseline is defined as the last assessment prior to the administration of study treatment. An ANCOVA model was fitted to total body PASI score at Day 29. The ANCOVA model included treatment as a fixed effect and baseline total body PASI score as a covariate. ^*^The adjusted treatment means are estimated using adjusted Least Square Means from the fitted model; ^**^The adjusted percentage change from baseline is derived as ((Adjusted treatment mean at Day 29 – global treatment mean at baseline)/global treatment mean at baseline)*100. Global treatment mean is the mean at baseline of all subjects included in the analysis, across all three treatment groups. **Abbreviations:** ANCOVA, analysis of covariance; BID, twice daily; CI, confidence interval; LS, least squares; PASI, psoriasis area and severity index.
(DOCX)

S1 Fig Chemical structure of DC-806. (TIF)

S1 Appendix DC-806 Phase 1c clinical study protocol. (PDF)

S2 Appendix CONSORT checklist. (DOCX)

## References

[1] Raharja A, Mahil SK, Barker JN. Psoriasis: a brief overview. Clin Med (Lond). 2021;21(3):170–3. doi: 10.7861/clinmed.2021-0257 34001566 PMC8140694

[2] Greb JE, Goldminz AM, Elder JT, Lebwohl MG, Gladman DD, Wu JJ, et al. Psoriasis. Nat Rev Dis Primers. 2016;2:16082. doi: 10.1038/nrdp.2016.82 27883001

[3] Nestle FO, Kaplan DH, Barker J. Psoriasis. N Engl J Med. 2009;361(5):496–509. doi: 10.1056/NEJMra0804595 19641206

[4] Hawkes JE, Chan TC, Krueger JG. Psoriasis pathogenesis and the development of novel targeted immune therapies. J Allergy Clin Immunol. 2017;140(3):645–53. doi: 10.1016/j.jaci.2017.07.004 28887948 PMC5600287

[5] Papp KA, Reid C, Foley P, Sinclair R, Salinger DH, Williams G, et al. Anti-IL-17 receptor antibody AMG 827 leads to rapid clinical response in subjects with moderate to severe psoriasis: results from a phase I, randomized, placebo-controlled trial. J Invest Dermatol. 2012;132(10):2466–9. doi: 10.1038/jid.2012.163 22622425

[6] Bausch Health Americas, Inc. Single-dose study to evaluate the safety, tolerability, pharmacokinetics and pharmacodynamics of AMG 827 (NCT00867100). ClinicalTrials.gov. 2023. https://clinicaltrials.gov/study/NCT00867100

[7] Wright JF, Guo Y, Quazi A, Luxenberg DP, Bennett F, Ross JF, et al. Identification of an interleukin 17F/17A heterodimer in activated human CD4+ T cells. J Biol Chem. 2007;282(18):13447–55. doi: 10.1074/jbc.M700499200 17355969

[8] McGeachy MJ, Cua DJ, Gaffen SL. The IL-17 family of cytokines in health and disease. Immunity. 2019;50(4):892–906. doi: 10.1016/j.immuni.2019.03.021 30995505 PMC6474359

[9] Novartis Pharma Corp. COSENTYX® (secukinumab) prescribing information. 2016. https://www.accessdata.fda.gov/drugsatfda_docs/label/2009/050715s027,050716s028lbl.pdf

[10] Eli Lilly & Co. TALTZ® (ixekizumab) prescribing information. 2021. https://www.accessdata.fda.gov/drugsatfda_docs/label/2021/125521s014lbl.pdf

[11] UCB Pharma. BIMZELX® (bimekizumab) prescribing information. 2023. https://www.accessdata.fda.gov/drugsatfda_docs/label/2023/761151s000lbl.pdf

[12] Amgen Inc. SILIQ™ (brodalumab) prescribing information. 2017. https://www.accessdata.fda.gov/drugsatfda_docs/label/2017/761032lbl.pdf

[13] Wu JJ, Wang CA, Jobson G, Davidson D, Kalirai S, Zhu J, et al. Treatment patterns and healthcare costs among patients with psoriasis initiating apremilast or biologics: a retrospective claims database cohort analysis. J Dermatolog Treat. 2023;34(1):2177095. doi: 10.1080/09546634.2023.2177095 36736349

[14] Bristol-Myers S. SOTYKTU® (deucravacitinib) prescribing information. 2022. https://www.accessdata.fda.gov/drugsatfda_docs/label/2022/214958s000lbl.pdf

[15] West-Ward Pharmaceuticals Corp. Methotrexate prescribing information. 2020. https://www.accessdata.fda.gov/drugsatfda_docs/label/2020/040054s015,s016,s017.pdf

[16] Amgen Inc. Otezla® (apremilast) prescribing information. 2021. https://www.accessdata.fda.gov/drugsatfda_docs/label/2021/205437s011lbl.pdf

[17] Novartis. Neoral® (cyclosporine) prescribing information. 2009. https://www.accessdata.fda.gov/drugsatfda_docs/label/2009/050715s027,050716s028lbl.pdf

[18] He H, Wu W, Zhang Y, Zhang M, Sun N, Zhao L, et al. Model-based meta-analysis in psoriasis: a quantitative comparison of biologics and small targeted molecules. Front Pharmacol. 2021;12:586827. doi: 10.3389/fphar.2021.586827 34276352 PMC8281289

[19] Sbidian E, Chaimani A, Garcia-Doval I, Doney L, Dressler C, Hua C, et al. Systemic pharmacological treatments for chronic plaque psoriasis: a network meta-analysis. Cochrane Database Syst Rev. 2022;5(5):CD011535. doi: 10.1002/14651858.CD011535.pub5 35603936 PMC9125768

[20] Eli Lilly and Company. A study to assess S011806 (DC-806 or LY4100504) in healthy adult participants and participants with chronic plaque psoriasis (NCT06808815). ClinicalTrials.gov. 2025. https://clinicaltrials.gov/study/NCT06808815

[21] Glatt S, Baeten D, Baker T, Griffiths M, Ionescu L, Lawson ADG, et al. Dual IL-17A and IL-17F neutralisation by bimekizumab in psoriatic arthritis: evidence from preclinical experiments and a randomised placebo-controlled clinical trial that IL-17F contributes to human chronic tissue inflammation. Ann Rheum Dis. 2018;77(4):523–32. doi: 10.1136/annrheumdis-2017-212127 29275332 PMC5890624

[22] Trentham DE, Townes AS, Kang AH. Autoimmunity to type II collagen an experimental model of arthritis. J Exp Med. 1977;146(3):857–68. doi: 10.1084/jem.146.3.857 894190 PMC2180804

[23] Mullard A. New plaque psoriasis approval carries suicide warning. Nat Rev Drug Discov. 2017;16(3):155. doi: 10.1038/nrd.2017.44 28248935

[24] R&D Systems, Inc. Quantikine® HS ELISA human IL-17 immunoassay HS170 product datasheet. 2017. https://resources.rndsystems.com/pdfs/datasheets/hs170.pdf?v=20230825&_ga=2.153649272.1763395342.1692973146-1396296758.1692973143

[25] Schofield C, Fischer SK, Townsend MJ, Mosesova S, Peng K, Setiadi AF, et al. Characterization of IL-17AA and IL-17FF in rheumatoid arthritis and multiple sclerosis. Bioanalysis. 2016;8(22):2317–27. doi: 10.4155/bio-2016-0207 27620302

[26] Kolbinger F, Loesche C, Valentin M-A, Jiang X, Cheng Y, Jarvis P, et al. β-Defensin 2 is a responsive biomarker of IL-17A-driven skin pathology in patients with psoriasis. J Allergy Clin Immunol. 2017;139(3):923-932.e8. doi: 10.1016/j.jaci.2016.06.038 27502297

[27] Soderstrom C, Berstein G, Zhang W, Valdez H, Fitz L, Kuhn M, et al. Ultra-sensitive measurement of IL-17A and IL-17F in psoriasis patient serum and skin. AAPS J. 2017;19(4):1218–22. doi: 10.1208/s12248-017-0094-4 28534291

[28] Fitz L, Zhang W, Soderstrom C, Fraser S, Lee J, Quazi A, et al. Association between serum interleukin-17A and clinical response to tofacitinib and etanercept in moderate to severe psoriasis. Clin Exp Dermatol. 2018;43(7):790–7. doi: 10.1111/ced.13561 29748971

[29] Alcusky M, Lee S, Lau G, Chiu GR, Hadker N, Deshpande A, et al. Dermatologist and patient preferences in choosing treatments for moderate to severe psoriasis. Dermatol Ther (Heidelb). 2017;7(4):463–83. doi: 10.1007/s13555-017-0205-2 29052800 PMC5698204

[30] Feldman SR, Holmen Moeller A, Erntoft Idemyr ST, González JM. Relative importance of mode of administration in treatment preferences among plaque psoriasis patients in the United States. J Health Econ Outcomes Res. 2016;4(2):141–57. doi: 10.36469/9817 37661952 PMC10471409

[31] Zheng J, Chen D, Xu J, Ding X, Wu Y, Shen HC, et al. Small molecule approaches to treat autoimmune and inflammatory diseases (Part III): Targeting cytokines and cytokine receptor complexes. Bioorg Med Chem Lett. 2021;48:128229. doi: 10.1016/j.bmcl.2021.128229 34214508

[32] Liu S, Dakin LA, Xing L, Withka JM, Sahasrabudhe PV, Li W, et al. Binding site elucidation and structure guided design of macrocyclic IL-17A antagonists. Sci Rep. 2016;6:30859. doi: 10.1038/srep30859 27527709 PMC4985813

[33] Zhang B, Dömling A. Small molecule modulators of IL-17A/IL-17RA: a patent review (2013-2021). Expert Opin Ther Pat. 2022;32(11):1161–73. doi: 10.1080/13543776.2022.2143264 36350977

[34] Eli Lilly and Company. A Safety Study of LY3462817 and LY3509754 in Participants with Psoriasis (NCT04152382). ClinicalTrials.gov. https://clinicaltrials.gov/study/NCT04152382

[35] Leo P. A single and multiple ascending-dose trial of LEO 153339 in healthy adults (NCT04883333). ClinicalTrials.gov. 2023. https://clinicaltrials.gov/study/NCT04883333

[36] Wong H, Liu L, Ouyang W, Deng Y, Wright MR, Hop CECA. Exposure-effect relationships in established rat adjuvant-induced and collagen-induced arthritis: a translational pharmacokinetic-pharmacodynamic analysis. J Pharmacol Exp Ther. 2019;369(3):406–18. doi: 10.1124/jpet.118.255562 30940693

[37] Schreiber S, Colombel J-F, Feagan BG, Reich K, Deodhar AA, McInnes IB, et al. Incidence rates of inflammatory bowel disease in patients with psoriasis, psoriatic arthritis and ankylosing spondylitis treated with secukinumab: a retrospective analysis of pooled data from 21 clinical trials. Ann Rheum Dis. 2019;78(4):473–9. doi: 10.1136/annrheumdis-2018-214273 30674475 PMC6530077

[38] Krueger JG, Wharton KA Jr, Schlitt T, Suprun M, Torene RI, Jiang X, et al. IL-17A inhibition by secukinumab induces early clinical, histopathologic, and molecular resolution of psoriasis. J Allergy Clin Immunol. 2019;144(3):750–63. doi: 10.1016/j.jaci.2019.04.029 31129129

[39] Patel DD, Lee DM, Kolbinger F, Antoni C. Effect of IL-17A blockade with secukinumab in autoimmune diseases. Ann Rheum Dis. 2013;72 Suppl 2:ii116-23. doi: 10.1136/annrheumdis-2012-202371 23253932

[40] Konrad RJ, Higgs RE, Rodgers GH, Ming W, Qian Y-W, Bivi N, et al. Assessment and clinical relevance of serum IL-19 levels in psoriasis and atopic dermatitis using a sensitive and specific novel immunoassay. Sci Rep. 2019;9(1):5211. doi: 10.1038/s41598-019-41609-z 30914699 PMC6435799

[41] Wolk K, Wilsmann-Theis D, Witte K, Brembach T-C, Kromer C, Gerdes S, et al. Interleukin-19 levels are increased in palmoplantar pustulosis and reduced following apremilast treatment. Int J Mol Sci. 2023;24(2):1276. doi: 10.3390/ijms24021276 36674793 PMC9862858

[42] Noordenbos T, Blijdorp I, Chen S, Stap J, Mul E, Cañete JD, et al. Human mast cells capture, store, and release bioactive, exogenous IL-17A. J Leukoc Biol. 2016;100(3):453–62. doi: 10.1189/jlb.3HI1215-542R 27034403

[43] US Center for Drug Evaluation and Research. SILIQ (brodalumab) clinical pharmacology and biopharmaceutics review(s). 2015. https://www.accessdata.fda.gov/drugsatfda_docs/nda/2017/761032Orig1s000ClinPharmR.pdf

[44] Witte E, Kokolakis G, Witte K, Philipp S, Doecke W-D, Babel N, et al. IL-19 is a component of the pathogenetic IL-23/IL-17 cascade in psoriasis. J Invest Dermatol. 2014;134(11):2757–67. doi: 10.1038/jid.2014.308 25046339

[45] Mallinckrodt CH, Lane PW, Schnell D, Peng Y, Mancuso JP. Recommendations for the primary analysis of continuous endpoints in longitudinal clinical trials. Drug Information J. 2008;42(4):303–19. doi: 10.1177/009286150804200402

