# Supplementary material for: Clinical proof of concept for small molecule mediated inhibition of IL-17 in psoriasis
Source: PLoS One. 2026 Jan 23;21(1):e0341049. doi: 10.1371/journal.pone.0341049 (PMC12829784; doi:10.1371/journal.pone.0341049)
Supplement: S1 Table — *The supernatant from each donor was used at a dilution that corresponded to the bioactivity of 1 ng/mL recombinant human IL-17A. Abbreviations: CXCL-1, chemokine (CXC motif) ligand 1; IL, interleukin; HEK: human embryonic kidney; KD, dissociation constant; mIL, murine interleukin; nM, nanomolar; SEAP, secretory alkaline phosphatase; SPR, surface plasmon resonance; TH17, T-helper 17. (DOCX) [file pone.0341049.s001.docx]

| **Assay** | **Readout** | **Stimulant** | **IL-17 Concentration (ng/ml)** | **Average Potency (nM)** | **N** |
| --- | --- | --- | --- | --- | --- |
| HEK-Blue IL-17 reporter cell line | SEAP | Recombinant human IL-17AA | 5.0 | 5.7 | 10 |
| HEK-Blue IL-17 reporter cell line | SEAP | Recombinant human IL-17AF | 5.0 | 130 | 12 |
| HEK-Blue IL-17 reporter cell line | SEAP | Recombinant human IL-17FF | 5.0 | >10,000 | 4 |
| HEK-Blue IL-17 reporter cell line | SEAP | Human TH17 supernatant | See methods* | 5.5 | 7 |
| Human oral keratinocytes | CXCL-1 | Recombinant human IL-17AA | 100.0 | 68 | 1 |
| Normal human epithelial keratinocytes | CXCL-1 | Recombinant human IL-17AA | 100.0 | 33 | 1 |
| HaCat immortalized human keratinocytes | CXCL-1 | Recombinant human IL-17AA | 5.0 | 4.6 | 1 |
| Human dermal fibroblasts | IL-6 | Recombinant human IL-17AA | 5.0 | 20 | 10 |
| NIH-3T3 | mIL-6 | Recombinant rat IL-17AA | 1.0 | 3.8 | 7 |
| NIH-3T3 | mIL-6 | Recombinant rat IL-17AF | 5.0 | 15 | 1 |
| **SPR** |  |  |  |  |  |
|  | **K_D_ (nM)** | **k_on_ (M^-1^s^-1^)** | **k_off_ (s^-1^)** | **t^1/2^** | **N** |
| IL-17AA | 1.6 | 3.1E+04 | 4.7E-05 | 4.2 hours | 4 |
| IL-17AF | 61 | 1.1E+4 | 6.9E-4 | 16.7 min | 2 |
