# Supplementary material for: Clinical proof of concept for small molecule mediated inhibition of IL-17 in psoriasis
Source: PLoS One. 2026 Jan 23;21(1):e0341049. doi: 10.1371/journal.pone.0341049 (PMC12829784; doi:10.1371/journal.pone.0341049)
Supplement: S2 Table — A total of 18 protocol deviations in 7 (22%) were classified as important. In the deviations categorized as assessment/procedure performed outside of permitted time window, 2 in the DC-806 group were related to COVID-19 and 2 in the placebo group were related to patient work commitments. Abbreviations: BID, twice daily. (DOCX) [file pone.0341049.s002.docx]

| **Category, n (%)** | **DC-806** | | | **Placebo**  **n=11** | **Total**  **N=32** |
| --- | --- | --- | --- | --- | --- |
|  | **200 mg BID**  **n=13** | **800 mg BID**  **n=8** | **Total**  **n=21** |  |  |
| Assessment/procedures not performed as per protocol | 2 (15) | 2 (25) | 4 (19) | 0 | 4 (13) |
| Assessment/procedure performed outside the permitted time window | 1 (8) | 1 (13) | 2 (10) | 2 (18) | 4 (13) |
| Incorrect dosage/administration of study medication | 5 (39) | 1 (13) | 6 (29) | 1 (9) | 7 (22) |
| Visit not performed/completed | 0 | 0 | 0 | 1 (9) | 1 (3) |
| Other (lost, incorrect study procedure worksheets) | 1 (8) | 1 (13) | 2 (10) | 0 | 2 (6) |
