# Supplementary material for: Clinical proof of concept for small molecule mediated inhibition of IL-17 in psoriasis
Source: PLoS One. 2026 Jan 23;21(1):e0341049. doi: 10.1371/journal.pone.0341049 (PMC12829784; doi:10.1371/journal.pone.0341049)
Supplement: S3 Table — N = the number of subjects in the analysis set. n is the number of subjects who have results at both baseline and Day 29. (%) = n/N*100. Baseline is defined as the last assessment prior to the administration of study treatment. An ANCOVA model was fitted to total body PASI score at Day 29. The ANCOVA model included treatment as a fixed effect and baseline total body PASI score as a covariate. *The adjusted treatment means are estimated using adjusted Least Square Means from the fitted model; **The adjusted percentage change from baseline is derived as ((Adjusted treatment mean at Day 29 – global treatment mean at baseline)/global treatment mean at baseline)*100. Global treatment mean is the mean at baseline of all subjects included in the analysis, across all three treatment groups. Abbreviations: ANCOVA, analysis of covariance; BID, twice daily; CI, confidence interval; LS, least squares; PASI, psoriasis area and severity index. (DOCX) [file pone.0341049.s003.docx]

|  | **DC-806** | | **Placebo**  **N=11** |
| --- | --- | --- | --- |
|  | **200 mg BID**  **N=13** | **800 mg BID**  **N=8** |  |
| Number of subjects included in the analysis n (%) | 10 (76.9) | 7 (87.5) | 10 (90.9) |
| Adjusted treatment mean (LS Mean) at Day 29^*^ | 5.74 | 3.80 | 5.86 |
| Standard error | 0.344 | 0.411 | 0.344 |
| 95% CI | 5.026, 6.451 | 2.951, 4.654 | 5.148, 6.571 |
| Adjusted percentage change from baseline^**^ | −15.05 | −43.71 | −13.26 |
| 95% CI | −25.602, −4.504 | −56.310, −31.113 | −23.795, −2.731 |
