# Supplementary figures and images for: Clinical proof of concept for small molecule mediated inhibition of IL-17 in psoriasis
Source: PLoS One. 2026 Jan 23;21(1):e0341049. doi: 10.1371/journal.pone.0341049 (PMC12829784; doi:10.1371/journal.pone.0341049)

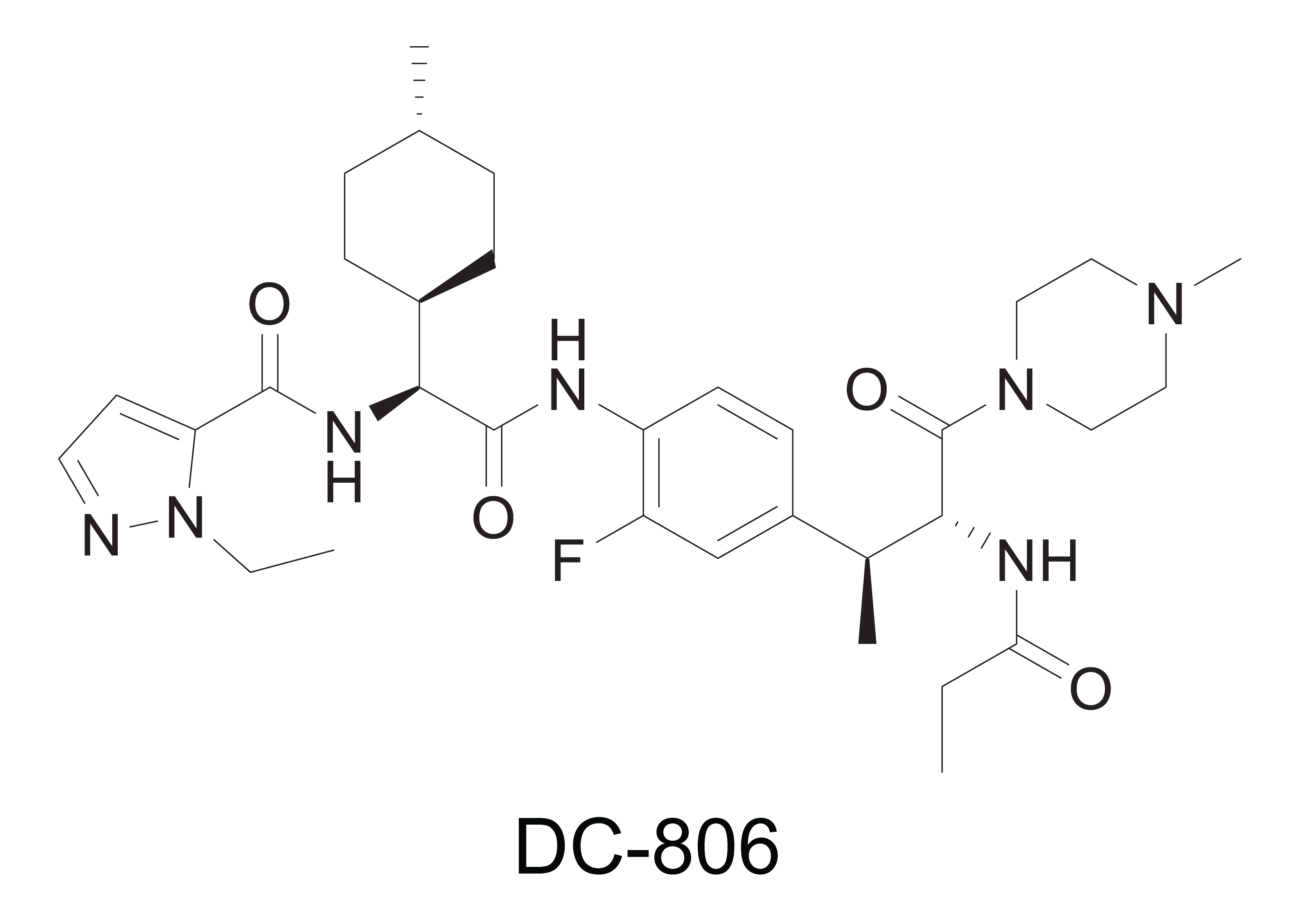

Supplement: S1 Fig — (TIF) [file pone.0341049.s004.tif]
