# Supplementary material for: Clinical proof of concept for small molecule mediated inhibition of IL-17 in psoriasis
Source: PLoS One. 2026 Jan 23;21(1):e0341049. doi: 10.1371/journal.pone.0341049 (PMC12829784; doi:10.1371/journal.pone.0341049)
Supplement: S1 Appendix — (PDF) [file pone.0341049.s005.pdf]

Clinical Study Protocol

Protocol Title: A Phase I, Randomized, Double-Blinded, Placebo-Controlled, 3-Part Study to Evaluate the Safety, Tolerability, Pharmacokinetics, and Pharmacodynamics of Single Ascending and Multiple Ascending Doses of S011806 in Healthy Participants and Multiple Doses of S011806 in Patients with Chronic Plaque Psoriasis

Protocol Number: DCE806101

Version: 5.0 11 February 2022

Compound: S011806

Study Phase: 1

Short Title: A Single and Multiple Ascending Dose Study of S011806

Sponsor Name: DiCE Alpha, Inc.

Legal Registered Address: 279 E. Grand Avenue, Suite 300, Lobby B, South San Francisco, CA 94080

Regulatory Agency Identifier Number:
EudraCT: 2021-002888-21

Approval Date: 11 February 2022

Confidentiality Statement

This document is confidential. The information contained in this document is the property of DiCE Alpha, Inc. and may not be reproduced, published or disclosed to others without written authorisation. Such information may be solely used for the purpose of reviewing or performing this study.

Sponsor Approval

Sponsor Signatory:

PPD

11-Feb-2022 | 06:19 PST

PPD

Date

Chief Medical Officer

DiCE Alpha, Inc.

Medical Monitor's Name and Contact Information

PPD

PPD (Serious Adverse Event [SAE] and pregnancy reporting)

PPD

<u>Sponsor Representative</u>

PPD

Chief Medical Officer

PPD

List of CRO and other Vendors

CRO and Monitoring:

PPD PPD

Safety Laboratory testing:

PPD PPD

Pharmacokinetic testing:

PPD PPD

Statistics and Data Management:

PPD PPD

Pharmacovigilance:

PPD PPD

Medical Monitoring:

PPD PPD

Qualified Person

PPD PPD

Investigator Signature Page

I have read this protocol.

I agree to comply with the current International Council for Harmonisation Guidelines for Good Clinical Practice and the laws, rules, regulations, and guidelines of the community, country, state, or locality relating to the conduct of the clinical study.

I also agree that persons debarred from conducting or working on clinical studies by any court or regulatory agency will not be allowed to conduct or work on studies for the Sponsor or a partnership in which the Sponsor is involved.

I will immediately disclose it in writing to the Sponsor if any person who is involved in the study is debarred or if any proceeding for debarment is pending or, to the best of my knowledge, threatened.

This document contains confidential information of the Sponsor, which must not be disclosed to anyone other than the recipient study staff and members of the Health Authority/Ethics Committee/Institutional Review Board. I agree to ensure that this information will not be used for any purpose other than the evaluation or conduct of the clinical study without the prior written consent of the Sponsor.

Investigator Signatory:

PPD

11-Feb-2022 | 12:48 GMT

Date

Name: PPD

Title: PPD

Affiliation:

PPD

Table of Contents

Sponsor Approval 3
Medical Monitor's Name and Contact Information 4
List of CRO and other Vendors 5
Investigator Signature Page 6
Table of Contents 7
List of Figures 14
List of Tables 14
1. Protocol Summary 15
1.1 Synopsis 15
1.2 Schedule of Activities 21
1.2.1 Part 1 Daily Schedule 21
1.2.2 Part 1 Daily Schedule – Food Effect Cohort 24
1.2.3 Part 1 Hourly Schedule 28
1.2.4 Part 2 Daily Schedule - 7-day dosing 30
1.2.5 Part 2 Daily Schedule – 10-day dosing 34
1.2.6 Part 2 Hourly Schedule Days 1 – 2 38
1.2.7 Part 2 Hourly Schedule Days 7 – 9 or 10 – 12 41
1.2.8 Part 3 Daily Schedule 43
1.2.9 Part 3 Hourly Schedule Days 1 and 22 47
2. Introduction 49
2.1 Background 49
2.2 Rationale 50
2.2.1 Rationale for Dose Selection 51

2.3 Benefit-Risk Assessment 55

3. Objectives and Endpoints 59

3.1 Primary Objectives (Safety) 59

3.1.1 Part 1 59

3.1.2 Part 2 59

3.1.3 Part 3 59

3.2 Secondary objectives (Plasma PK of S011806) 59

3.2.1 Part 1 59

3.2.2 Part 2 59

3.2.3 Part 3 59

3.3 Exploratory Objectives 60

3.3.1 Part 1 60

3.3.2 Part 2 60

3.3.3 Part 3 60

3.3.4 Estimands 60

3.4 Endpoints 60

3.4.1 Primary Endpoints (Safety) 60

3.4.2 Secondary Endpoints (Plasma PK of S011806) 61

3.4.3 Exploratory Endpoints 61

4. Study Design 63

4.1 Overall Study Design 63

4.2 Part 1 (Healthy Subject SAD) 64

4.2.1 Part 1 (Healthy Subject SAD): Food-Effect Cohort 65

4.3 Part 2 (Healthy Subject MAD) 66

4.4 Part 3 (Psoriasis Patients) 67

4.5 End of Study 69
5. Study Population 70
5.1 Inclusion Criteria 70
5.1.1 Inclusion Criteria for Healthy Subjects (Parts 1 and 2) 70
5.1.2 Inclusion Criteria for Patients with Psoriasis (Part 3) 71
5.2 Exclusion Criteria 72
5.2.1 Exclusion Criteria for Healthy Subjects (Parts 1 and 2) 72
5.2.2 Exclusion Criteria for Patients with Psoriasis (Part 3) 75
5.3 Screen Failures 78
5.4 Rescreening 78
5.5 Study Restrictions 79
5.5.1 Prior and Concomitant Medication 79
5.5.2 Permitted Concomitant Medication for Healthy Subjects (Parts 1 and 2) .. 79
5.5.3 Permitted Concomitant Medication for Patients with Psoriasis (Part 3) 79
5.5.4 Prohibited Concomitant Medication for Healthy Subjects (Parts 1 and 2) . 80
5.5.5 Prohibited Concomitant Medication for Patients with Psoriasis (Part 3) 81
5.5.6 Lifestyle Considerations 82
5.5.7 Meals and Dietary Restrictions 82
5.5.7.1 Meals and Dietary Restrictions Parts 1 and 2 82
5.5.7.2 Meals and Dietary Restrictions Parts 3 83
5.5.8 Alcohol Restrictions 83
5.5.9 Activity Restrictions 84
5.5.10 Additional Restrictions 84
6. Investigational Medicinal Products 85
6.1 S011806 and Placebo 85

6.2 Administration of Investigational Medicinal Product 85
6.2.1 In-Clinic Administration of IMP 87
6.2.2 At-home Administration of IMP (Part 3 only) 87
6.3 Preparation, Handling, Storage and Accountability 88
6.4 Minimisation of Bias 89
6.4.1 Randomisation and Subject/Patient Numbering 89
6.4.2 Blinding 89
6.4.3 Procedures for Breaking the Blind Prior to Study Completion 90
6.5 Investigational Product Compliance 91
6.6 Criteria for Dose Escalation (Parts 1 and 2) 91
6.7 Stopping Criteria 93
7. Discontinuation of Investigational Medicinal Product and Subject/Patient Discontinuation or Withdrawal 96
7.1 Discontinuation of Investigational Medicinal Product 96
7.2 Subject/Patient Replacement 97
7.3 Subject/Patient Discontinuation or Withdrawal from the Study 97
7.4 Lost to Follow Up 98
8. Study Assessments and Procedures 99
8.1 COVID-19 screening 99
8.2 Screening 99
8.3 Admission and Pre-dose Procedures 99
8.4 Timing of Procedures 100
8.5 Discharge from the Clinical Research Unit 100
8.6 Return Visits 101
8.7 Early Discontinuation Procedures and Visits 101

8.8 Blood Volume 101
8.9 Medical Supervision 102
8.10 Safety Assessments 102
8.10.1 Physical Examinations 102
8.10.2 Vital Signs 102
8.10.3 Body Weight and Height 103
8.10.4 Electrocardiograms 103
8.10.5 Cardiac Holter Monitoring (Parts 1 and 2 only) 103
8.10.6 Additional Safety Procedures 104
8.10.7 Clinical Safety Laboratory Assessments 104
8.10.7.1 Haematology, Clinical Chemistry, Coagulation and Virology 104
8.10.7.2 SARS-CoV-2 (Nasal/Throat swab) 104
8.10.7.3 Urinalysis 105
8.10.7.4 Pregnancy Test 105
8.10.7.5 Follicle-Stimulating Hormone (FSH) Test 105
8.10.7.6 Drug Screen and Urine Cotinine 105
8.10.7.7 Alcohol Breath Test 106
8.10.7.8 Abnormal Laboratory Findings 106
8.10.7.9 Columbia Suicide Severity Rating Scale (C-SSRS) 107
8.11 Adverse Events and Serious Adverse Events 107
8.11.1 Time Period and Frequency for Collecting AE and SAE Information 107
8.11.2 Method of Detecting AEs and SAEs 108
8.11.3 Follow-up of AEs and SAEs 108
8.11.4 Regulatory Reporting Requirements for SAEs 108
8.11.5 Pregnancy 109

8.11.6 Adverse Events of Special Interest ... 109
8.12 Treatment of Overdose ... 110
8.13 Pharmacokinetic Assessments ... 110
8.13.1 Pharmacokinetic Blood Sampling ... 110
8.13.2 Pharmacokinetic Urine Sampling (Selected cohorts in Part 2 only) ... 111
8.14 Pharmacodynamic Assessments -Part 3 only ... 111
8.14.1 Pharmacodynamic Blood Sampling ... 111
8.14.2 Skin Plaque Biopsy ... 111
8.15 Clinical Activity - Part 3 only ... 112
8.15.1 Percent of Body Surface Area Involvement (BSA) ... 112
8.15.2 Psoriasis Area and Severity Index Score (PASI) ... 112
8.15.3 Physician Global Assessment (PGA) ... 112
8.15.4 Lesion Severity Score (LSS) ... 112
8.15.5 Photography of Lesion Site ... 112
9. Statistical Considerations ... 114
9.1 Sample Size Determination ... 114
9.2 Population for Analyses ... 114
9.3 General Analyses ... 115
9.3.1 Demographic Data and Baseline Characteristics ... 115
9.3.2 Safety Analysis ... 116
9.3.3 PK Analysis ... 116
9.3.4 PD Analysis (Part 3 Only) ... 118
9.3.5 Clinical Activity Analysis (Part 3 only) ... 118
9.4 Interim Analysis ... 119
9.5 Handling of Missing Data Points ... 119

9.6 Data Monitoring Committee (DMC) ... 119

10. Supporting Documentation and Operational Considerations ... 120

10.1 Appendix 1: Regulatory, Ethical, and Study Oversight Considerations ... 120

10.1.1 Regulatory and Ethical Considerations ... 120

10.1.2 Financial Disclosure ... 121

10.1.3 Informed Consent Process ... 121

10.1.4 Data Protection ... 122

10.1.5 Committees Structure ... 122

10.1.6 Dissemination of Clinical Study Data ... 122

10.1.7 Data Quality Assurance ... 122

10.1.8 Source Documents ... 123

10.1.9 Study and Site Closure ... 124

10.2 Appendix 2: Clinical Laboratory Tests ... 125

10.3 Appendix 3: Adverse Events and Serious Adverse Events: Definitions and Procedures for Recording, Evaluating, Follow-up, and Reporting ... 126

10.3.1 Definition of AE ... 126

10.3.2 Definition of SAE ... 127

10.3.3 Recording and Follow-Up of AE and/or SAE ... 128

10.3.4 Reporting of SAEs/Pregnancies ... 130

10.4 Appendix 4: Contraceptive Guidance, Exposure, Sperm Donation and Collection of Pregnancy Information ... 131

10.4.1 Contraception ... 131

10.4.2 Exposure to Partners during the Study ... 133

10.4.3 Sperm and Ova/oocytes Donation ... 133

10.4.4 Collection of Pregnancy Information ... 133

10.5 Appendix 5: Summary of Protocol Changes ... 135

References..154

Abbreviations 156

List of Figures

Figure 1. Overall Study Design and Dose-escalation Scheme 64

List of Tables

Table 1. Planned S011806 Doses in Part 1 (SAD) Cohorts 53
Table 2. Food Effect Cohort High Fat Meal Guidance [16] 86
Table 3. Study and Cohort Stopping Rules 94

1. Protocol Summary

1.1 Synopsis

Study number	DCE806101
Study title	A Phase I, Randomized, Double-Blinded, Placebo-Controlled, 3-Part Study to Evaluate the Safety, Tolerability, Pharmacokinetics, and Pharmacodynamics of Single Ascending and Multiple Ascending Doses of S011806 in Healthy Participants and Multiple Doses of S011806 in Patients with Chronic Plaque Psoriasis
Number of centres	The study is planned to be conducted entirely by a single site, Medicines Evaluation Unit (MEU), Manchester, UK, however additional sites may be added if required.
Investigational medicinal product details	Active: S011806 oral tablets and matching placebo
Objectives	**Primary Objectives (Safety):**<br>**Part 1**<br>To assess the safety and tolerability of a single ascending oral dose of S011806 in healthy subjects.<br>**Part 2**<br>To assess the safety and tolerability of multiple ascending oral doses of S011806 in healthy subjects.<br>**Part 3**<br>To assess the safety and tolerability of multiple oral doses of S011806 in patients with psoriasis.<br><br>**Secondary Objectives (Plasma PK of S011806):**<br>**Part 1**<br>To characterise systemic PK of S011806 after a single ascending oral dose in healthy subjects.<br>To determine the effect of food on a single oral dose of S011806 in healthy subjects.<br>**Part 2**<br>To characterise systemic PK of S011806 after multiple ascending oral doses in healthy subjects.<br>**Part 3**<br>To evaluate systemic PK of S011806 after multiple oral doses in patients with psoriasis.<br><br>**Exploratory Objectives:**<br>**Part 1**<br>To potentially conduct preliminary metabolite profiling and characterisation of S011806 in healthy subjects with human plasma in selected cohorts.<br><br>**Part 2**<br>To assess the renal elimination of S011806 in healthy subjects following oral administration of multiple ascending doses of S011806 in selected cohorts<br>To conduct preliminary metabolite profiling and characterisation of S011806

	in healthy subjects with human plasma and/or urine in selected cohorts.<br><br>**Part 3**<br>To assess exploratory PD endpoints following multiple oral doses of S011806 in patients with psoriasis.<br>To explore preliminary clinical activity of S011806 in psoriasis patients after 4- week treatment.<br>To explore potential drug exposure-response relationship based on, but not limited to, the PASI score.
Endpoints	**Primary Endpoints (Safety):**<br>Safety and tolerability will be assessed by monitoring of AEs, physical examinations, C-SSRS, changes in vital signs, clinical laboratory parameters, cardiac Holter monitoring and ECGs.<br><br>**Secondary Endpoints (Plasma PK of S011806):**<br>Quantification of S011806 in plasma will be performed using High-performance Liquid Chromatography with tandem mass spectrometry (LC-MS/MS) method followed by calculation of pharmacokinetic parameters, including but not limited to:<br>In Parts 1 and 2, calculation of pharmacokinetic parameters, including but not limited to; C_{max}, T_{max}, AUC and $t_{1/2.}$<br>In addition, in the Part 1 food effect cohort, relative bioavailability of S011806 following a high-fat breakfast versus fasted state based on AUC_{inf} and C_{max} will be assessed.<br>In Part 3, observed C_{max} on Days 1 and 22, trough concentrations on Days 2, 8, 15, 22 and 29 will be reported.<br>Dose and time dependency will be assessed for the pharmacokinetic parameters.<br><br>**Exploratory Endpoints:**<br>Plasma and urine PK samples collected during Part 1 and Part 2 of the study may also be used for exploratory research, including but not limited to, metabolite profiling and identification.<br><br>In Part 2, calculation of urine PK parameters including but not limited to Ae, Ae_{t1-t2}, Clr and Fe and metabolite analysis (identification) will be assessed as data permit.<br>In Part 3, Exploratory PD biomarkers will include, but not be limited to, levels of inflammatory cytokines (e.g. IL-17A) and beta defensin-2 (BD-2) in serum. Exploratory PD biomarkers will also include but not be limited to changes in histology by hematoxylin and eosin staining and RNA analysis including but not limited to gene expression including IL-17A, F and beta-defensin 2 in skin biopsies. Exploratory clinical activity will include but not be limited to change in PASI, PGA, BSA and LSS assessment. Clinical activity will also include evaluation of data from photography of lesion sites as data permit. Potential drug exposure-response relationship based on PASI score will also be assessed.

Summary of study design	This is an integrated Phase 1, 3-part, double-blinded, randomized, placebo-controlled SAD/SAD Food-effect (Part 1) and MAD (Part 2) study in healthy subjects and multiple dose study in patients with psoriasis (Part 3).<br><br>Safety, tolerability, and PK will be assessed following either single ascending (SAD, Part 1) or multiple ascending (MAD, Part 2) oral dosing of S011806 in a tablet formulation. In Part 3, safety, tolerability, PK, exploratory PD and clinical activity will be assessed following once, twice or three times daily oral doses of S011806 in tablet formulation.<br><br>**Part 1:**<br>Part 1 will comprise up to 5 cohorts, each containing approximately 8 subjects planned to receive a single dose of S011806 or placebo (6:2 active: placebo) for a planned total of up to 40 subjects. The starting dose for cohort 1 will be 25 mg.<br><br>In each cohort, subjects will receive a single oral dose of S011806 or placebo on the morning of Day 1 with approximately 240ml water. Subjects will be required to fast (water permitted) for at least 10 hours prior to and 4 hours post dose. Water is not permitted in the Food Effect cohort (both treatment periods) for 1 hour prior to - and 1 hour post-dose with the exception of the water required to swallow the IMP. One cohort, the food-effect cohort, will return for a second treatment period as detailed in Section 4.2.1. Each cohort, including both treatment periods in the food-effect cohort, will include a sentinel group of 2 subjects (1:1, active:placebo) who will be dosed at least 24 hours prior to dosing the remaining 6 subjects (5:1, active:placebo). The remaining subjects will only be dosed if the sentinel group shows no clinically significant safety or tolerability concerns at the discretion of the Investigator (e.g. review of AE's, vital signs and ECGs).<br><br>Subjects will undergo safety and tolerability assessments (e.g. physical examinations, vital signs, 12-lead ECG, cardiac Holter monitoring, clinical laboratory tests, and AE monitoring), and PK evaluations at specified time points during the study (see Section 1.2.1 and 1.2.3). Subjects will remain resident at the CRU until Day 3 after completion of safety assessments at 48 hours post-dose (and longer if required by emerging data) and providing there are no safety concerns, they will be discharged from the unit. They will return to the CRU on Day 8 (± 2 days) for safety evaluations to be performed as detailed in Section 1.2.1.<br><br>Subjects will be screened for eligibility to participate in the study within 28 days before dosing (Day 1) and will be admitted to the CRU on the evening of Day -1.<br><br>**Part 2:**<br>Part 2 will comprise 4 separate cohorts each containing 8 subjects (6 active: 2 placebo) planned to receive once, twice or three times daily doses of S011806 or placebo between Day 1 and 7 or Day 1 and 10.<br><br>The planned level, frequency, and duration of S011806 doses to be

	administered in Part 2 will be confirmed during the interim review of safety and available PK data from Part 1 cohorts.<br><br>Part 2 cohorts may be initiated in parallel with the conduct of the Part 1 cohorts once it has been determined by the SAC that adequate safety and exposure data have been generated after completion of dosing of a Part 1 cohort one dose level higher than the planned first dose in Part 2. No Part 2 dose will exceed the maximum dose studied in Part 1.<br><br>Following the first dose of IMP (Day 1), subjects will remain resident in the CRU until completion of safety assessments at 48 hours post final dose (Day 9 for 7-day dosing or Day 12 for 10-day dosing). In the event of twice daily dosing, evening doses will be administered approximately 12 hours (±1 hour) after the morning dose. In the event of three times daily dosing, subjects will be dosed approximately 8 hours apart from the earlier dose. Subjects will undergo safety and tolerability (i.e., physical examinations, vital signs, 12-lead ECG, cardiac Holter monitoring, clinical laboratory tests, C-SSRS and AE monitoring), and PK evaluations at specified time points during this time (see Section 1.2.4, 1.2.5, 1.2.6 and 1.2.7). Providing there are no safety concerns, they will be discharged from the unit and return to the CRU for safety evaluations to be performed as detailed in Section 1.2.4 and 1.2.5 on Day 14 (± 2 days) or 17 (± 2 days), for 7- or 10-day dosing respectively.<br><br>Subjects will be screened for eligibility to participate in the study within 28 days before dosing (Day 1) and will be admitted to the CRU on the evening of Day -1.<br><br>**Part 3:**<br>Part 3 will comprise 2 separate cohorts each containing up to 21 patients with psoriasis (planned total of up to 42 patients) planned to receive S011806 or placebo (active:placebo ratio of 2:1) once, twice or three times daily for 28 days.<br><br>The planned level and frequency of S011806 doses to be administered in Part 3 will be confirmed during the interim review of safety and available PK data from completed Part 1 and 2 cohorts.<br><br>Part 3 cohorts may commence in parallel with the conduct of Part 2 cohorts once it has been determined by the SAC that adequate safety and exposure data have been generated from the Part 2 cohorts. As a minimum, Part 3 can commence once the same dose level in Part 2 has been completed and reviewed by the SAC. Part 3 cohort 2 may commence prior to the completion of Part 3 cohort 1 providing the same dose level in Part 2 has been completed and reviewed by the SAC. No single dose will exceed the maximum dose studied in Part 1 or 2.<br><br>Following the first dose of IMP (Day 1), patients will remain resident in the CRU overnight and until the completion of Day 2 pre-dose procedures and will receive the Day 2 morning dose prior to discharge. Patients will undergo safety and tolerability (i.e., physical examinations, vital signs, 12-lead ECG,

	clinical laboratory tests, C-SSRS and AE monitoring), PK and PD evaluations at specified time points during this time (see Section 1.2.9). Providing there are no safety concerns, patients will be discharged from the unit and return on Days 8, 15, 22, and 29 for safety, PK, PD and clinical evaluations and IMP administration as detailed in Section 1.2.8 and 1.2.9. On days when patients are not required to attend the CRU, IMP dosing will be completed by the patient at home at the time of the day consistent with the time of the dosing during Day 1. Patients will complete a daily dosing diary during periods of home dosing. Patients will return to the CRU on Day 43 (± 2 days) for safety and PD evaluations to be performed as detailed in Section1.2.8.<br>Subjects will be screened for eligibility to participate in the study within 28 days before dosing (Day 1).
Number of subjects	**Part 1** (SAD): Approximately 40 subjects (up to 5 cohorts of 8 subjects)<br>**Part 2** (MAD): Approximately 32 subjects (up to 4 cohorts of 8 subjects)<br>**Part 3** (Psoriasis): Approximately 42 patients (2 cohorts of up to 21 patients)<br>Subjects who discontinue may be replaced at the discretion of the Sponsor upon discussion with the Investigator.
Duration of the study	**Part 1** (SAD): Subjects will receive a single dose of S011806 or placebo in on one occasion. The estimated time from screening to the end of study is approximately 5 weeks.<br>**Part 1 Food-effect:** Subjects will receive a single dose of S011806 or placebo in on two occasions. The estimated time from screening to the end of study is approximately 7 weeks<br>**Part 2** (MAD): Subjects will receive one, two or three doses of S011806 or placebo daily for 7 or 10 days. The estimated time from screening to the end of study is approximately 6 weeks for 7-day dosing and approximately 7 weeks for 10-day dosing.<br>**Part 3** (Psoriasis): Subjects will receive one, two or three doses of S011806 or placebo daily for 28 days. The estimated time from screening to the end of study is approximately 11 weeks.
Interim data reviews	The data reviews will be conducted by the SAC, which will comprise the Sponsor's physician (or delegate) and the Principal Investigator (or delegate) as a minimum. Additional data available at the time of each scheduled meeting may also be reviewed.<br><br>Dose levels for Part 1, dose duration for Part 2 and dose levels and frequency of dosing for Part 2 and 3 will be confirmed during the SAC review and may be modified when appropriate based on available safety and PK data.<br><br>**Part 1** (SAD):<br>All pertinent safety/tolerability data (e.g., physical examinations, vital signs assessments, safety 12–lead ECGs, clinical laboratory tests, and AEs) and all available PK blood and plasma data at least 24 hours post dose will be reviewed to inform dose escalation decisions. In addition, available PK data from previous cohorts will be included in the review, for context. At least 6 of the planned subjects for each dose level must complete dosing and data review for the next dose level to proceed.

	**Part 2** (MAD)<br>All pertinent safety/tolerability data (e.g., physical examinations, vital signs assessments, safety 12–lead ECGs, clinical laboratory tests, C-SSRS and AEs) at least 48 hours post final dose will be reviewed to inform dose escalation decisions. At least 6 of the planned subjects for each dose level must complete dosing and data review for the next dose level to proceed.<br><br>At the Sponsor's request, unblinded safety and efficacy tables, figures, and data listings may be presented to the Development Leadership Team for the purposes of planning future clinical studies prior to database lock. Unblinding of anyone directly involved in the conduct of the study will not occur for the purpose of this interim analysis.
Safety assessments	Adverse events Columbia-Suicide Severity Rating Scale (C-SSRS) – Part 2 and Part 3<br>Clinical chemistry, haematology, coagulation and urinalysis<br>Vital signs (blood pressure, pulse rate and temperature)<br>Electrocardiogram<br>Cardiac Holter monitoring<br>Physical examination
Statistical methodology	Statistical analysis will be descriptive and exploratory. Continuous data will be summarised using descriptive statistics (n, mean, SD, median, minimum, and maximum) and categorical data will be summarised using the number and percentage of subjects/patients in each category.<br>Data will be presented separately for Parts 1, 2, and 3 and as appropriate by treatment group and dose. Data from placebo treated subjects/patients will be pooled across dosing cohorts within each study part.<br>Full details of the statistical analyses will be provided in a separate statistical analysis plan (SAP).

1.2 Schedule of Activities

1.2.1 Part 1 Daily Schedule

Study Procedures [a]	Phone Screening	Screening [b]	Check-In [c]	Treatment Phase [d]		EOT [e]	Follow Up/ET [f]
Days →			Day -1	Day 1	Day 2	Day 3	Day 8 (± 2)
			Resident in CRU				
**Procedures**							
COVID-19 Screening Questioning	X	X	X				X
Informed Consent		X					
Inclusion/Exclusion Criteria	X	X	X				
Medical and Surgical History	X	X					
**Safety Evaluations**							
Physical Examination [g]		X(F)		X(A)		X(A)	X(A)
Symptom-Driven Physical Examinations [h]					X		
Height		X					
Weight		X		X			X
12-Lead Safety ECG [i]		X		X	X	X	X
Vital Signs (Supine blood pressure and heart rate)		X		X	X	X	X
Vital Signs (Temperature)		X	X	X	X	X	X
Haematology, Serum Chemistry and Coagulation [j, k]		X		X	X	X	X
Urinalysis [k]		X		X	X	X	X
Urine or Serum Pregnancy (WOCBP only) [k, m]		X		X			X
Serum FSH (Post-menopausal females only) [n]		X					
Urine Drug Screen		X	X				

Study Procedures [a]	Phone Screening	Screening [b]	Check-In [c]	Treatment Phase [d]		EOT [e]	Follow Up/ET [f]
Days →			Day -1	Day 1	Day 2	Day 3	Day 8 (± 2)
			Resident in CRU				
Urine Cotinine Screen		X	X				
Breath Alcohol Screen		X	X				
HIV/Hepatitis Screen		X					
SARS-CoV 2 (COVID-19) throat/nasal swab [l]			X [l]				
Tuberculosis Test (QuantiFERON® TB Gold)		X					
Adverse Event Monitoring (from consent onwards)		X					
Concomitant Medication Monitoring	X	X					
Study Drug Administration, Pharmacokinetics, Pharmacodynamics and Cardiodynamics							
IMP Administration				X			
Plasma for S011806 Pharmacokinetics				X	X	X	X
Cardiodynamic Holter Monitoring [o]				X			

a. For details on Procedures, refer to Section 8. Additional evaluations/testing may be conducted if deemed necessary by the Investigator or designee and/or the Sponsor for reasons related to subject safety.
b. Within 28 days prior to study drug administration.
c. Subjects will be admitted to the CRU on Day -1, at the time indicated by the CRU.
d. Hourly schedule for Days 1-3 is detailed in Section 1.2.3.
e. To be performed at EOT prior to discharge from the CRU.
f. All subjects who received the dose of study drug (including subjects who discontinue the study early) will return to the CRU 7 (±2) days after the study drug administration for follow-up procedures, and to determine if any AEs have occurred since the last study visit or prior to early discontinuation from

the study if withdrawn early. Subjects who discontinue the study early will undergo all procedures scheduled for early termination prior to discharge from the CRU. At the discretion of the Investigator, procedures scheduled for early discontinuation may not be repeated if already conducted on the same day.

g. A full PE (F) is required at Screening. An abbreviated PE (A) will be conducted at other scheduled time points.
h. Symptom-driven physical examinations will be performed at the Investigator's or designee's discretion, as needed, and at other times to focus on evaluation of AEs throughout the study.
i. At Screening and baseline (pre-dose Day 1), triplicate 12-lead ECG will be conducted. At all other scheduled time points, 12-lead safety ECGs will be conducted as a single 12- lead ECG using the site's local standard ECG machine.
j. Samples for serum chemistry will be obtained following a fast of at least 8 hours (water permitted).
k. If subjects screening laboratory tests are conducted more than 7 days prior to dosing laboratory tests must be repeated within 7 days of day 1. Therefore, if screening is scheduled within 7 days of day 1, all laboratory tests can be performed at screen only.
l. COVID19 test will be conducted on, and/or prior to Day -1 with results available prior to dose on Day 1
m. Serum pregnancy test will be conducted at Screening, at the follow-up visit and at the Early Termination visit (if applicable). Urine pregnancy test will be conducted pre-dose on Day 1. Pregnancy testing will only be performed on women of childbearing potential (WOCBP).
n. To be conducted for postmenopausal women who did not undergo any sterilization procedures as detailed in Section 10.4 and where postmenopausal state is in question.
o. Holter monitors will be used to collect continuous 12-lead ECG data starting on Day 1 at least 1 hour prior to dosing and until at least 24 hours post-dose (Day 2).

Abbreviations: CRU = Clinical research unit, ECG = Electrocardiogram, EOT = End of Treatment, ET= Early Termination Visit, FSH = Follicle-stimulating hormone, FU = Follow-up, HIV = Human immunodeficiency virus, PCR = Polymerase Chain Reaction, and PE = Physical examination, WOCBP = Women of childbearing potential

1.2.2 Part 1 Daily Schedule – Food Effect Cohort

Study Procedures [a]	Phone Screening	Screening [b]	Check-In [c]	Treatment Phase [d]				Check-In [c]	Treatment Phase [d]		EOT [e]	Follow Up/ET [f]
Days →			Day -1	Day 1	Day 2	Day 3	Day 8 (± 2)	Day -1[r]	Day 1[q]	Day 2	Day 3	Day 8 (± 2)
			Treatment period 1 - Fasted					Treatment period 2 - Fed				
			Resident in CRU					Resident in CRU				
**Procedures**												
COVID-19 Screening Questioning	X	X	X				X	X				X
Informed Consent		X										
Inclusion/Exclusion Criteria	X	X	X									
Medical and Surgical History	X	X										
**Safety Evaluations**												
Physical Examination [g]		X(F)		X(A)		X(A)	X(A)		X(A)		X(A)	X(A)
Symptom-Driven Physical Examinations [h]			X									
Height		X										
Weight		X		X			X		X			X
12-Lead Safety ECG [i]		X		X	X	X	X		X	X	X	X
Vital Signs (Supine blood pressure and heart rate)		X		X	X	X	X		X	X	X	X
Vital Signs (Temperature)		X	X	X	X	X	X	X	X	X	X	X

Study Procedures [a]	Phone Screening	Screening [b]	Check-In [c]	Treatment Phase [d]				Check-In [c]	Treatment Phase [d]		EOT [e]	Follow Up/ET [f]
Days →			Day -1	Day 1	Day 2	Day 3	Day 8 (± 2)	Day -1[r]	Day 1[g]	Day 2	Day 3	Day 8 (± 2)
			Treatment period 1 - Fasted					Treatment period 2 - Fed				
			Resident in CRU					Resident in CRU				
Haematology, Serum Chemistry and Coagulation [i]		X [k]		X	X	X	X		X	X	X	X
Urinalysis		X [k]		X	X	X	X		X	X	X	X
Urine or Serum Pregnancy (WOCBP only) [m]		X [k]		X			X		X			X
Serum FSH (Post-menopausal females only) [n]		X										
Urine Drug Screen		X	X					X				
Urine Cotinine Screen		X	X					X				
Breath Alcohol Screen		X	X					X				
HIV/Hepatitis Screen		X										
SARS-CoV 2 (COVID-19) throat/nasal swab [l]			X [l]					X [l]				
Tuberculosis Test (QuantiFERON® TB Gold)		X										
Adverse Event Monitoring (from consent onwards)		X										

Study Procedures [a]	Phone Screening	Screening [b]	Check-In [c]	Treatment Phase [d]				Check-In [c]	Treatment Phase [d]		EOT [e]	Follow Up/ET [f]
Days →			Day -1	Day 1	Day 2	Day 3	Day 8 (± 2)	Day -1[r]	Day 1[q]	Day 2	Day 3	Day 8 (± 2)
			Treatment period 1 - Fasted					Treatment period 2 - Fed				
			Resident in CRU					Resident in CRU				
Concomitant Medication Monitoring	X						X					
Study Drug Administration, Pharmacokinetics, and Cardiodynamics												
IMP Administration				X					X[p]			
Plasma for S011806 Pharmacokinetics				X	X	X	X		X	X	X	X
Cardiodynamic Holter Monitoring [o]				X					X			

a. For details on Procedures, refer to Section 8. Additional evaluations/testing may be conducted if deemed necessary by the Investigator or designee and/or the Sponsor for reasons related to subject safety.
b. Within 28 days prior to study drug administration.
c. Subjects will be admitted to the CRU on Day -1, at the time indicated by the CRU.
d. Hourly schedule for Days 1-3 is detailed in Section 1.2.3.This applies to treatment period 1 and 2.
e. To be performed at EOT prior to discharge from the CRU.
f. All subjects who received the dose of study drug (including subjects who discontinue the study early) will return to the CRU 7 (±2) days after the study drug administration for follow-up procedures, and to determine if any AEs have occurred since the last study visit, or prior to early discontinuation from the study if withdrawn early. Subjects who discontinue the study early will undergo all procedures scheduled for early termination prior to discharge from the CRU. At the discretion of the Investigator, procedures scheduled for early discontinuation may not be repeated if already conducted on the same day.
g. A full PE (F) is required at Screening. An abbreviated PE (A) will be conducted at other scheduled time points.
h. Symptom-driven physical examinations will be performed at the Investigator's or designee's discretion, as needed, and at other times to focus on evaluation of AEs throughout the study.
i. At Screening and baseline (pre-dose Day 1), triplicate 12-lead ECG will be conducted. At all other scheduled time points, 12-lead safety ECGs will be conducted as a single 12- lead ECG using the site's local standard ECG machine.
j. Samples for serum chemistry will be obtained following a fast of at least 8 hours (water permitted).

k. If subjects screening laboratory tests are conducted more than 7 days prior to dosing laboratory tests must be repeated within 7 days of day 1. Therefore, if screening is scheduled within 7 days of day 1, all laboratory tests can be performed at screen only.
l. COVID19 test will be conducted with results available prior to dose on Day 1
m. Serum pregnancy test will be conducted at Screening, Day 8 (TP1), at the follow-up visit and at the Early Termination visit (if applicable). Urine pregnancy test will be conducted pre-dose on Day 1. Pregnancy testing will only be performed on women of childbearing potential (WOCBP).
n. To be conducted for postmenopausal women who did not undergo any sterilization procedures as detailed in Section 10.4 and where postmenopausal state is in question.
o. Holter monitors will be used to collect continuous 12-lead ECG data starting on Day 1 at least 1 hour prior to dosing and for at least 24 hours post-dose (Day 2).
p. Prior to IMP administration in treatment period 2, subjects will eat a high fat breakfast as detailed in Section 6.2.
q. TP2 Day 1 must be a minimum of 5 half-lives or 7 days from TP1 Day 1, whichever is longer. If subjects TP1 follow up laboratory tests are conducted more than 7 days prior to dosing in TP2, laboratory tests must be repeated within 7 days of TP2 Day 1.
r. TP1 Day 8 may occur on the same day as TP2 Day -1.

Abbreviations: CRU = Clinical research unit, ECG = Electrocardiogram, EOT = End of Treatment, ET = Early Termination Visit, FSH = Follicle-stimulating hormone, FU = Follow-up, HIV = Human immunodeficiency virus, PCR = Polymerase Chain Reaction, PE = Physical examination, TP = Treatment Period and WOCBP = Women of childbearing potential

1.2.3 Part 1 Hourly Schedule

Study Procedures [a] Days →	Day 1												Day 2		Day 3
Hours →	Pre-Dose	0	0.5	1	1.5	2	3	4	6	8	12	16	24	36	48
Procedures [b]															
Abbreviated Physical Examination	X														X
Symptom-Driven Physical Examinations [c]									X						
Weight	X														
12-Lead Safety ECG [d]	X			X		X	X	X		X	X		X		X
Vital Signs (Supine blood pressure and heart rate)	X			X		X	X	X	X	X	X		X		X
Vital Signs (Temperature)	X												X		X
Haematology, Serum Chemistry and Coagulation [e]	X												X		X
Urinalysis	X												X		X
Urine Pregnancy (WOCBP only)	X														
Adverse Event Monitoring (from consent onwards)									X						
Concomitant Medication Monitoring									X						
IMP Administration		X													
Plasma for S011806 Pharmacokinetics	X		X	X	X	X	X	X	X	X	X	X	X	X	X
Cardiodynamic Holter Monitoring [f]							X								

a. For details on Procedures, refer to Section 8.
b. Additional evaluations/testing may be conducted if deemed necessary by the Investigator or designee and/or the Sponsor for reasons related to subject safety.
c. Symptom-driven physical examinations will be performed at the Investigator's or designee's discretion, as needed, and at other times to focus on evaluation of AEs throughout the study.
d. 12-lead safety ECGs will be collected in triplicate at baseline (pre-dose) and as single 12-lead ECG at other scheduled time points using the site's local standard ECG machine.
e. Samples for serum chemistry will be obtained following a fast of at least 8 hours (water permitted).
f. Holter monitors will be used to collect continuous 12-lead ECG data starting on Day 1 at least 1 hour prior to dosing and up to at least 24 hours post-dose (Day 2).

Abbreviations: ECG = Electrocardiogram, IMP = Investigation Medicinal Product and WOCBP = Women of childbearing potential

1.2.4 Part 2 Daily Schedule - 7-day dosing

Study Procedures [a]	Phone Screening	Screening [b]	Check-In [c]	Treatment Phase [d, e]							EOT [e, f]		Follow Up/ET [g]
Days →			Day -1	Day 1	Day 2	Day 3	Day 4	Day 5	Day 6	Day 7	Day 8	Day 9	Day 14 (± 2)
			Resident in CRU										
Procedures													
COVID-19 Screening Questioning	X	X	X										X
Informed Consent		X											
Inclusion/Exclusion Criteria	X	X	X										
Medical and Surgical History	X	X											
C-SSRS		X		X		X [s]			X [s]			X	X
Safety Evaluations													
Physical Examination [h]		X(F)		X(A)						X(A)		X(A)	X(A)
Symptom-Driven Physical Examinations [i]		X											
Height		X											
Weight		X		X								X	X
12-Lead Safety ECG [j]		X		X	X					X	X		X
Vital Signs (Supine blood pressure and heart rate)		X		X	X	X [s]	X [s]	X [s]	X [s]	X	X	X	X
Vital Signs (Temperature)		X	X	X	X	X [s]	X [s]	X [s]	X [s]	X	X	X	X
Haematology, Serum Chemistry and Coagulation [k, l]		X		X	X					X	X	X	X

Study Procedures [a]	Phone Screening	Screening [b]	Check-In [c]	Treatment Phase [d, e]							EOT [e, f]		Follow Up/ET [g]
Days →			Day -1	Day 1	Day 2	Day 3	Day 4	Day 5	Day 6	Day 7	Day 8	Day 9	Day 14 (± 2)
			Resident in CRU										
Urinalysis [l]		X		X	X					X	X	X	X
Urine or Serum Pregnancy (WOCBP only) [l, n]		X		X									X
Serum FSH (Post-menopausal females only) [o]		X											
Urine Drug Screen		X	X										
Urine Cotinine Screen		X	X										
Breath Alcohol Screen		X	X										
HIV/Hepatitis Screen		X											
SARS-CoV 2 (COVID-19) throat/nasal swab [m]			X										
Tuberculosis Test (QuantiFERON® TB Gold)		X											
Adverse Event Monitoring (from consent onwards)		X											
Concomitant Medication Monitoring		X											
Study Drug Administration, Pharmacokinetics, Pharmacodynamics and Cardiodynamics													
IMP Administration [p, t]				X	X	X	X	X	X	X			
Plasma for [S11806] Pharmacokinetics (QD regimens)				X	X [s]		X [s]		X [s]	X	X	X	X

Study Procedures [a]	Phone Screening	Screening [b]	Check-In [c]	Treatment Phase [d, e]							EOT [e, f]		Follow Up/ET [g]
Days →			Day -1	Day 1	Day 2	Day 3	Day 4	Day 5	Day 6	Day 7	Day 8	Day 9	Day 14 (± 2)
			Resident in CRU										
Plasma for [S11806] Pharmacokinetics (BID regimens)				X	X [s]		X [s]		X [s]	X	X	X	X
Plasma for [S11806] Pharmacokinetics (TID regimens)				X	X [s]		X [s]		X [s]	X	X	X	X
Urine for S011806 Pharmacokinetics [q]				X [q]						X [q]			
Cardiodynamic Holter Monitoring [r]				X						X			

a. For details on Procedures, refer to Section 8. Additional evaluations/testing may be conducted if deemed necessary by the Investigator or designee and/or the Sponsor for reasons related to subject safety.
b. Within 28 days prior to study drug administration.
c. Subjects will be admitted to the CRU on Day -1, at the time indicated by the CRU.
d. Hourly schedule for Days 1-2 is indicated in Section 1.2.6
e. Hourly schedule for Days 7 – 9 is detailed in Section 1.2.7
f. To be performed at EOT prior to discharge from the CRU.
g. All subjects who received the dose of study drug (including subjects who discontinue the study early) will return to the CRU 7 (±2) days after the study drug administration for follow-up procedures, and to determine if any AEs have occurred since the last study visit. or prior to early discontinuation from the study if withdrawn early. Subjects who discontinue the study early will undergo all procedures scheduled for early termination prior to discharge from the CRU. At the discretion of the Investigator, procedures scheduled for early discontinuation may not be repeated if already conducted on the same day.
h. A full PE (F) is required at Screening. An abbreviated PE (A) will be conducted at other scheduled time points.
i. Symptom-driven physical examinations will be performed at the Investigator's or designee's discretion, as needed, and at other times to focus on evaluation of AEs throughout the study.
j. At Screening and baseline (pre-dose Day 1), triplicate 12-lead ECG will be conducted. At all other scheduled time points, 12-lead safety ECGs will be conducted as a single 12- lead ECG using the site's local standard ECG machine.
k. Samples for serum chemistry will be obtained following a fast of at least 8 hours (water permitted).

l. If subjects screening laboratory tests are conducted more than 7 days prior to dosing laboratory tests must be repeated within 7 days of day 1. Therefore, if screening is scheduled within 7 days of day 1, all laboratory tests can be performed at screen only.
m. COVID19 test will be conducted on, and/or prior to Day -1 with results available prior to dose on Day 1.
n. Serum pregnancy test will be conducted at Screening, at the follow-up visit and at the Early Termination visit (if applicable). Urine pregnancy test will be conducted pre-dose on Day 1. Pregnancy testing will only be performed on women of childbearing potential (WOCBP).
o. To be conducted for postmenopausal women who did not undergo any sterilization procedures as detailed in Section 10.4 and where postmenopausal state is in question.
p. In the event of twice daily dosing, evening dosing will be approximately 12 (±1) h post morning dose Day 1-6.
q. Urine collection intervals (in selected cohorts) are pre-dose, and 0-6, 6-12, 12-24 hours post-dose on Day 1, pre-dose and 0-6, 6-12 hours post-dose on Day 2 and pre-dose, and 0-6, 6-12, 12-24, 24-36 hours post-dose Day 7 to 8. Urine samples may be used for metabolite analysis as well, at the discretion of the Sponsor.
r. Holter monitors will be used to collect continuous 12-lead ECG data starting on Day 1 at least 1 hour prior to dosing and for at least 24 hours post-dose (Day 2) and Day 7 at least 1 hour prior to dosing for at least 24-hours post-dose (Day 8).
s. To be performed prior to morning dose.
t. In the event of three times daily dosing, doses will be approximately 8 hours apart Day 1 - 6.

Abbreviations: CRU = Clinical research unit, ECG = Electrocardiogram, EOT = End of Treatment, ET = Early Termination Visit, FSH = Follicle-stimulating hormone, FU = Follow-up, HIV = Human immunodeficiency virus, PCR = Polymerase Chain Reaction, PE = Physical examination and WOCBP = Women of childbearing potential

1.2.5 Part 2 Daily Schedule – 10-day dosing

Study Procedures [a]	Phone Screening	Screening [b]	Check-In [c]	Treatment Phase [d, e]										EOT [e, f]		Follow Up/ET [g]
Days →			Day -1	Day 1	Day 2	Day 3	Day 4	Day 5	Day 6	Day 7	Day 8	Day 9	Day 10	Day 11	Day 12	Day 17 (± 2)
			Resident in CRU													
Procedures																
COVID-19 Screening Questioning	X	X	X													X
Informed Consent		X														
Inclusion/Exclusion Criteria	X	X	X													
Medical and Surgical History	X	X														
C-SSRS		X		X		X [s]			X [s]			X [s]			X	X
Safety Evaluations																
Physical Examination [h]		X(F)		X(A)									X(A)		X(A)	X(A)
Symptom-Driven Physical Examinations [i]		X														
Height		X														
Weight		X		X											X	X
12-Lead Safety ECG [j]		X		X	X								X	X		X
Vital Signs (Supine blood pressure and heart rate)		X		X	X	X [s]	X [s]	X [s]	X [s]	X [s]	X [s]	X [s]	X [s]	X	X	X
Vital Signs (Temperature)		X	X	X	X	X [s]	X [s]	X [s]	X [s]	X [s]	X [s]	X [s]	X [s]	X	X	X

Study Procedures [a]	Phone Screening	Screening [b]	Check-In [c]	Treatment Phase [d, e]										EOT [e, f]		Follow Up/ET [g]
Days →			Day -1	Day 1	Day 2	Day 3	Day 4	Day 5	Day 6	Day 7	Day 8	Day 9	Day 10	Day 11	Day 12	Day 17 (± 2)
			Resident in CRU													
Haematology, Serum Chemistry and Coagulation [k, l]		X		X	X								X	X	X	X
Urinalysis [l]		X		X	X								X	X	X	X
Urine or Serum Pregnancy (WOCBP only) [l, n]		X		X												X
Serum FSH (Post-menopausal females only) [o]		X														
Urine Drug Screen		X	X													
Urine Cotinine Screen		X	X													
Breath Alcohol Screen		X	X													
HIV/Hepatitis Screen		X														
SARS-CoV 2 (COVID-19) throat/nasal swab [m]			X													
Tuberculosis Test (QuantiFERON® TB Gold)		X														
Adverse Event Monitoring (from consent onwards)		X														

Study Procedures [a]	Phone Screening	Screening [b]	Check-In [c]	Treatment Phase [d, e]										EOT [e, f]		Follow Up/ET [g]
Days →			Day -1	Day 1	Day 2	Day 3	Day 4	Day 5	Day 6	Day 7	Day 8	Day 9	Day 10	Day 11	Day 12	Day 17 (± 2)
			Resident in CRU													
Concomitant Medication Monitoring		X														
Study Drug Administration, Pharmacokinetics, Pharmacodynamics and Cardiodynamics																
IMP Administration [p, t]				X	X	X	X	X	X	X	X	X	X			
Plasma for [S11806] Pharmacokinetics (QD regimens)				X	X [s]		X [s]			X [s]			X	X	X	X
Plasma for [S11806] Pharmacokinetics (BID regimens)				X	X [s]		X [s]			X [s]			X	X	X	X
Plasma for [S11806] Pharmacokinetics (TID regimens)				X	X [s]		X [s]			X [s]			X	X	X	X
Urine for S011806 Pharmacokinetics [q]				X									X			
Cardiodynamic Holter Monitoring [r]				X									X			

a. For details on Procedures, refer to Section 8. Additional evaluations/testing may be conducted if deemed necessary by the Investigator or designee and/or the Sponsor for reasons related to subject safety.
b. Within 28 days prior to study drug administration.
c. Subjects will be admitted to the CRU on Day -1, at the time indicated by the CRU.
d. Hourly schedule for Days 1-2 is indicated in Section 1.2.6

e. Hourly schedule for Days 10 – 12 is detailed in Section 1.2.7
f. To be performed at EOT prior to discharge from the CRU.
g. All subjects who received the dose of study drug (including subjects who discontinue the study early) will return to the CRU 7 (±2) days after the study drug administration for follow-up procedures, and to determine if any AEs have occurred since the last study visit, or prior to early discontinuation from the study if withdrawn early. Subjects who discontinue the study early will undergo all procedures scheduled for early termination prior to discharge from the CRU. At the discretion of the Investigator, procedures scheduled for early discontinuation may not be repeated if already conducted on the same day.
h. A full PE (F) is required at Screening. An abbreviated PE (A) will be conducted at other scheduled time points.
i. Symptom-driven physical examinations will be performed at the Investigator's or designee's discretion, as needed, and at other times to focus on evaluation of AEs throughout the study.
j. At Screening and baseline (pre-dose Day 1), triplicate 12-lead ECG will be conducted. At all other scheduled time points, 12-lead safety ECGs will be conducted as a single 12- lead ECG using the site's local standard ECG machine.
k. Samples for serum chemistry will be obtained following a fast of at least 8 hours (water permitted).
l. If subjects screening laboratory tests are conducted more than 7 days prior to dosing laboratory tests must be repeated within 7 days of day 1. Therefore, if screening is scheduled within 7 days of day 1, all laboratory tests can be performed at screen only.
m. COVID19 test will be conducted on, and or prior to Day -1 with results available prior to dose on Day 1.
n. Serum pregnancy test will be conducted at Screening, at the follow-up visit and at the Early Termination visit (if applicable). Urine pregnancy test will be conducted pre-dose on Day 1. Pregnancy testing will only be performed on women of child bearing potential (WOCBP).
o. To be conducted for postmenopausal women who did not undergo any sterilization procedures as detailed in Section 10.4 and where postmenopausal state is in question.
p. In the event of twice daily dosing, evening dosing will be approximately 12 (±1) h post morning dose Day 1-9.
q. Urine collection intervals are pre-dose, and 0-6, 6-12, 12-24 hours post-dose on Day 1, pre-dose and 0-6, 6-12 hours post-dose on Day 2 and pre-dose, and 0-6, 6-12, 12-24, 24-36 hours post-dose on Day 10 to 11 in selected cohorts. Urine samples may be used for metabolite analysis as well, at the discretion of the Sponsor.
r. Holter monitors will be used to collect continuous 12-lead ECG data starting on Day 1 at least 1 hour prior to dosing and for at least 24 hours post-dose (Day 2) and Day 10 at least 1 hour prior to dosing for at least 24-hours post-dose (Day 11).
s. To be performed prior to morning dose.
t. In the event of three times daily dosing, doses will be approximately 8 hours apart Day 1 - 9.

Abbreviations: CRU = Clinical research unit, ECG = Electrocardiogram, EOT = End of Treatment, ET = Early Termination Visit, FSH = Follicle-stimulating hormone, FU = Follow-up, HIV = Human immunodeficiency virus, PCR = Polymerase Chain Reaction, PE = Physical examination and WOCBP = Women of childbearing potential

1.2.6 Part 2 Hourly Schedule Days 1 – 2

Study Procedures [a] Days →	Day 1												Day 2		
Hours →	Pre-Dose	0	0.5	1	1.5	2	3	4	6	8	12	16	0 (D1 24)	8	12
Procedures [b]															
Abbreviated Physical Examination	X														
C-SSRS	X														
Symptom-Driven Physical Examinations [c]	X														
Weight	X														
12-Lead Safety ECG [d]	X			X		X		X		X [m]	X [i]		X [h]		
Vital Signs (Supine blood pressure and heart rate)	X			X		X		X	X	X [m]	X [i]		X [h]		
Vital Signs (Temperature)	X												X [h]		
Haematology, Serum Chemistry and Coagulation [e]	X												X [h]		
Urinalysis	X												X [h]		
Urine Pregnancy (WOCBP only)	X														
Adverse Event Monitoring (from consent onwards)	X														
Concomitant Medication Monitoring	X														
IMP Administration		X								X [l]	X [g]	X [l]	X	X [l]	X [g]
Plasma for [S11806] Pharmacokinetics (QD	X		X	X	X	X	X	X	X	X	X		X [h]		X

Study Procedures [a] Days →	Day 1												Day 2		
Hours →	Pre-Dose	0	0.5	1	1.5	2	3	4	6	8	12	16	0 (D1 24)	8	12
Procedures [b]															
regimens) [i]															
Plasma for [S11806] Pharmacokinetics (BID regimens) [j]	X		X	X	X	X	X	X	X	X	X [i]		X [h]		
Plasma for [S11806] Pharmacokinetics (TID regimens) [j]	X		X	X	X	X	X	X	X	X [m]	X		X [h]		
Urine for S011806 Pharmacokinetics [k]	X														
Cardiodynamic Holter Monitoring [f]	X														

a. For details on Procedures, refer to Section 8.
b. Additional evaluations/testing may be conducted if deemed necessary by the Investigator or designee and/or the Sponsor for reasons related to subject safety.
c. Symptom-driven physical examinations will be performed at the Investigator’s or designee’s discretion, as needed, and at other times to focus on evaluation of AEs throughout the study.
d. 12-lead safety ECGs will be collected in triplicate at baseline (pre-dose) and as single 12-lead ECG at other scheduled time points using the site's local standard ECG machine.
e. Samples for serum chemistry will be obtained following a fast of at least 8 hours (water permitted).
f. Holter monitors will be used to collect continuous 12-lead ECG data starting on Day 1 at least 1 hour prior to dosing and up to at least 24 hours post-dose (Day 2).
g. Second dose at 12h and 36h only required in the event of twice daily dosing.
h. To be performed prior to morning dose on Day 2.
i. In the event of twice daily dosing, to be performed prior to evening dose.
j. PK sampling may be modified based on interim review of SAD data. Blood volumes indicated in Section 8.8 will not be exceeded.

k. Urine collection intervals (in selected cohorts) are pre-dose, and 0-6, 6-12, 12-24 hours post-morning dose Day 1 and pre-dose, and 0-6, 6-12 hours post dose on Day 2. Urine samples may be used for metabolite analysis as well, at the discretion of the Sponsor.
l. Second and third dose at 8h and 16h on Day 1 and second dose at 32h on Day 2 only required in the event of TID dosing.
m. In the event of TID dosing, to be performed prior to dosing at 8h

Abbreviations: ECG = Electrocardiogram, IMP = Investigation Medicinal Product and WOCBP = Women of childbearing potential

1.2.7 Part 2 Hourly Schedule Days 7 – 9 or 10 – 12

Study Procedures [a] Days →	Day 7 or 10											Day 8 or 11		Day 9 or 12
Hours →	Pre-Dose	0	0.5	1	1.5	2	3	4	6	8	12	24	36	48
Procedures [b]														
Abbreviated Physical Examination	X													X
C-SSRS														X
Symptom-Driven Physical Examinations [c]								X						
Weight														X
12-Lead Safety ECG [d]	X			X		X		X		X	X	X		
Vital Signs (Supine blood pressure and heart rate)	X			X		X		X	X	X	X	X		X
Vital Signs (Temperature)	X											X		X
Haematology, Serum Chemistry and Coagulation [e]	X											X		X
Urinalysis	X											X		X
Adverse Event Monitoring (from consent onwards)								X						
Concomitant Medication Monitoring								X						
IMP Administration		X												
Plasma for [S11806] Pharmacokinetics (QD regimens) [g]	X		X	X	X	X	X	X	X	X	X	X	X	X

Study Procedures [a] Days →	Day 7 or 10											Day 8 or 11		Day 9 or 12
Hours →	Pre-Dose	0	0.5	1	1.5	2	3	4	6	8	12	24	36	48
Procedures [b]														
Plasma for [S11806] Pharmacokinetics (BID regimens) [g]	X		X	X	X	X	X	X	X	X	X	X	X	X
Plasma for [S11806] Pharmacokinetics (TID regimens) [g]	X		X	X	X	X	X	X	X	X	X	X	X	X
Urine for S011806 Pharmacokinetics [h]							X							
Cardiodynamic Holter Monitoring [f]						X								

a. For details on Procedures, refer to Section 8.
b. Additional evaluations/testing may be conducted if deemed necessary by the Investigator or designee and/or the Sponsor for reasons related to subject safety.
c. Symptom-driven physical examinations will be performed at the Investigator's or designee's discretion, as needed, and at other times to focus on evaluation of AEs throughout the study.
d. Single 12-lead ECG will be collected at all scheduled time points using the site's local standard ECG machine.
e. Samples for serum chemistry will be obtained following a fast of at least 8 hours (water permitted).
f. Holter monitors will be used to collect continuous 12-lead ECG data starting on Day 7 or 10 at least 1 hour prior to dosing and up to at least 24 hours post-dose (Day 8 or 11).
g. PK sampling may be modified based on interim review of SAD data. Blood volumes indicated in Section 8.8 will not be exceeded.
h. Urine collection intervals (in selected cohorts) are pre-dose, and 0-6, 6-12, 12-24, 24-36 hours post-morning dose Day 7 to 8 or 10 to 11 . Urine samples may be used for metabolite analysis as well, at the discretion of the Sponsor.

Abbreviations: ECG = Electrocardiogram, IMP = Investigation Medicinal Product and WOCBP = Women of childbearing potential

1.2.8 Part 3 Daily Schedule

Study Procedures [a]	Phone Screening	Screening [b]	Baseline[c]	Treatment Phase									EOT [e]	Follow Up/ET [f]
Days →			Day -5 to Day -1	Day 1 [d]	Day 2	Day 3 - 7	Day 8	Day 9-14	Day 15	Day 16-21	Day 22 [d]	Day 23-28	Day 29	Day 43
Window (days)	-	-		Overnight residency		-	(± 2 days)	-	(± 2 days)	-	(± 2 days)	-	-	(± 2 days)
Procedures														
COVID-19 Screening Questioning	X	X	X	X			X		X		X		X	X
Informed Consent		X												
Inclusion/Exclusion Criteria	X	X	X	X										
Medical and Surgical History	X	X												
C-SSRS		X		X	X [p]		X [p]		X [p]		X		X	X
Safety Evaluations														
Physical Examination [g]		X(F)		X(A)			X(A) [p]		X(A) [p]		X(A) [p]		X(A)	X(A)
Symptom-Driven Physical Examinations [h]		X												
Height		X												
Weight		X		X[p]									X	X
12-Lead Safety ECG [i]		X		X	X [p]		X [p]		X [p]		X [p]		X	X
Vital Signs (Supine blood pressure and heart rate)		X		X	X [p]		X [p]		X [p]		X [p]		X	X

Study Procedures [a]	Phone Screening	Screening [b]	Baseline[c]	Treatment Phase									EOT [e]	Follow Up/ET [f]
Days →			Day -5 to Day -1	Day 1 [d]	Day 2	Day 3 - 7	Day 8	Day 9-14	Day 15	Day 16-21	Day 22 [d]	Day 23-28	Day 29	Day 43
Window (days)	-	-		Overnight residency		-	(± 2 days)	-	(± 2 days)	-	(± 2 days)	-	-	(± 2 days)
Vital Signs (Temperature)		X	X	X	X [p]		X [p]		X [p]		X [p]		X	X
Haematology, Serum Chemistry and Coagulation [j, k]		X		X	X [p]		X [p]		X [p]		X [p]		X	X
Urinalysis [k]		X		X	X [p]		X [p]		X [p]		X [p]		X	X
Urine or Serum Pregnancy (WOCBP only) [k, m]		X		X			X [p]		X [p]		X [p]		X	X
Serum FSH (Post-menopausal females only) [n]		X												
Urine Drug Screen		X		X										
Breath Alcohol Screen		X		X										
HIV/Hepatitis Screen		X												
SARS-CoV 2 (COVID-19) throat/nasal swab [l]				X										
Tuberculosis Test (QuantiFERON®)		X												

Study Procedures [a]	Phone Screening	Screening [b]	Baseline[c]	Treatment Phase									EOT [e]	Follow Up/ET [f]
Days →			Day -5 to Day -1	Day 1 [d]	Day 2	Day 3 - 7	Day 8	Day 9-14	Day 15	Day 16-21	Day 22 [d]	Day 23-28	Day 29	Day 43
Window (days)	-	-		Overnight residency		-	(± 2 days)	-	(± 2 days)	-	(± 2 days)	-	-	(± 2 days)
Adverse Event Monitoring (from consent onwards)		X												
Concomitant Medication Monitoring		X												
Study Drug Administration, Pharmacokinetics, Pharmacodynamics, and Dermatology														
IMP Dispensation and/or Accounting				X	X		X		X		X		X	
IMP Administration at CRU [o]				X	X		X		X		X			
IMP Administration at Home [o]					X	X	X	X	X	X	X	X		
Daily dosing Diary				X										
Plasma for S011806 Pharmacokinetics				X	X [p]		X [p]		X [p]		X		X	X
Serum for Biomarkers		X	X	X	X [p]		X [p]		X [p]		X		X	X[r]
Dermatology Assessment [q]		X	X				X [p]		X [p]		X [p]		X	X
Skin Biopsy			X										X	X[r]
Photography of lesion sites			X						X				X	X

a. For details on Procedures, refer to Section 8. Additional evaluations/testing may be conducted if deemed necessary by the Investigator or designee and/or the Sponsor for reasons related to subject safety.
b. Within 28 days prior to study drug administration.
c. Baseline dermatology assessments to be performed between Day -5 and -1 prior to Day 1.
d. Hourly Schedule for Day 1 and Day 22 is detailed in Section 1.2.9.
e. To be performed at EOT prior to discharge from the CRU.
f. All subjects who received the dose of study drug (including subjects who discontinue the study early) will return to the CRU 15 (±2) days after the last study drug administration for follow-up procedures, and to determine if any AEs have occurred since the last study visit, or prior to early discontinuation from the study if withdrawn early. Subjects who discontinue the study early will undergo all procedures scheduled for early termination prior to discharge from the CRU. At the discretion of the Investigator, procedures scheduled for early discontinuation may not be repeated if already conducted on the same day.
g. A full PE (F) is required at Screening. An abbreviated PE (A) will be conducted at other scheduled time points.
h. Symptom-driven physical examinations will be performed at the Investigator's or designee's discretion, as needed, and at other times to focus on evaluation of AEs throughout the study.
i. At Screening and baseline (pre-dose Day 1), triplicate 12-lead ECG will be conducted. At all other scheduled time points, 12-lead safety ECGs will be conducted as a single 12- lead ECG using the site's local standard ECG machine.
j. Samples for serum chemistry will be obtained following a fast of at least 8 hours (water permitted).
k. If subjects screening laboratory tests are conducted more than 7 days prior to dosing laboratory tests must be repeated within 7 days of day 1. Therefore, if screening is scheduled within 7 days of day 1, all laboratory tests can be performed at screen only.
l. A COVID-19 test will be conducted on, and/or prior to Day 1 with results available prior to dose on Day 1.
m. Serum pregnancy test will be conducted at Screening, at the follow-up visit and at the Early Termination visit (if applicable). Urine pregnancy test will be conducted pre-dose on Day 1, 8, 15, 22 and on Day 29. Pregnancy testing will only be performed on women of childbearing potential (WOCBP).
n. To be conducted for postmenopausal women who did not undergo any sterilization procedures as detailed in Section10.4 and where postmenopausal state is in question.
o. In the event of twice daily dosing, evening dosing will be approximately 12 hours post morning dose (±1h hour). In the event of three times daily dosing, doses will be approximately 8 hours apart. Morning dose will be administered in the CRU on CRU visit days, additional doses will be administered at home as applicable for twice and three times daily dosing. Last dose of IMP is taken on the evening of Day 28.
p. To be performed prior to morning dose.
q. Dermatology assessment includes PASI, BSA, LSS and PGA (Section 8.15).
r. Serum for biomarkers and skin biopsies are not required at FU. To be collected in the event of ET prior to Day 29 only.

Abbreviations: BSA = Body Surface Area CRU = Clinical research unit, ECG = Electrocardiogram, EOT = End of Treatment, ET = Early Termination Visit, FSH = Follicle-stimulating hormone, FU = Follow-up, HIV = Human immunodeficiency virus, LSS = Lesion Severity Score, PASI = Psoriasis Area and Severity Index, PCR = Polymerase Chain Reaction, PE = Physical examination, PGA = Physician Global Assessment and WOCBP = Women of child bearing potential

1.2.9 Part 3 Hourly Schedule Days 1 and 22

Study Procedures [a]										
Days →	Day 1 and 22							Day 1 Only		
Hours →	Pre-Dose	0	0.5	1	2	4	6	8	12	16
Procedures [b]										
Abbreviated Physical Examination	X									
Symptom-Driven Physical Examinations [c]	X									
C-SSRS	X									
12-Lead Safety ECG [d]	X			X	X	X	X			
Vital Signs (Supine blood pressure and heart rate)	X			X	X	X	X	X[f]	X[g]	
Vital Signs (Temperature)	X							X[f]	X[g]	
Haematology, Serum Chemistry and Coagulation [e]	X									
Urinalysis	X									
Urine Pregnancy (WOCBP only)	X									
Adverse Event Monitoring (from consent onwards)	X									
Concomitant Medication Monitoring	X									
IMP Administration		X						X[h]	X[i]	X[h]
Plasma for S011806 Pharmacokinetics	X		X	X	X	X	X		X[j]	
Serum for Biomarkers	X			X	X		X			

a. For details on Procedures, refer to Section 8.
b. Additional evaluations/testing may be conducted if deemed necessary by the Investigator or designee and/or the Sponsor for reasons related to subject safety.
c. Symptom-driven physical examinations will be performed at the Investigator's or designee's discretion, as needed, and at other times to focus on evaluation of AEs throughout the study.
d. 12-lead safety ECGs will be collected in triplicate at baseline (pre-dose on Day 1) and as single 12-lead ECG at other scheduled time points using the site's local standard ECG machine.
e. Samples for serum chemistry will be obtained following a fast of at least 8 hours (water permitted).
f. In the event of TID dosing, to be performed prior to dosing at 8h
g. In the event of twice daily dosing, to be performed prior to 12h evening dose
h. Second and third dose at 8h and 16h on Day 1 only required in the event of TID dosing.
i. Second dose at 12h on Day 1 only required in the event of BID dosing.
j. Sample to be taken prior to the evening dose in the event of BID dosing.

Abbreviations: ECG = Electrocardiogram, IMP = Investigation Medicinal Product and WOCBP = Women of child bearing potential

2. Introduction

2.1 Background

Psoriasis is a chronic, relapsing inflammatory disease of the skin with a reported prevalence range across countries of 0.09% to 11.4% [1, 2] affecting approximately 1-3% of the world's population. While psoriasis can manifest at any age, a bimodal age distribution during the 2nd to 4th and 6th and 7th decades of life [3]. The most common form is plaque psoriasis representing 80-90% of all cases. Plaque psoriasis is defined by well-defined/demarcated red, scaly plaques or patches. It can occur anywhere on the body but most commonly affects extensor surfaces such as elbows and knees, gluteal folds, scalp, and trunk.

While the etiology of psoriasis is unknown, genetic susceptibility and environmental triggers may play key role in the aberrant immune response resulting in disordered keratinocyte differentiation and proliferation and inflammation. The importance of the cytokine IL-17A in the pathogenesis of psoriasis was suggested by preclinical models of psoriasis and confirmed by the profound efficacy of inhibiting this signaling pathway via anti-IL17A (secukinumab and ixekizumab) and anti-IL17R (brodalumab) antibodies [4-6].

IL-17A is a member of the IL-17 cytokine family comprising 6 related proteins, IL-17A through F. IL-17A forms a homodimer (IL-17A/A) as well as a heterodimer with IL-17F (IL-17A/F) which are secreted by inflammatory cells, primarily TH17 cells, and signal through a heterodimeric receptor complex of IL-17RA and IL-17RC. IL-17AA primarily acts on epithelial cells, endothelial cells, and fibroblasts. IL-17AA signaling at epithelial surfaces plays an important role in mucosal defense and protective role in regulation of commensal colonization. Absence or impaired IL-17 signaling either genetic deficiency of IL-17 or treatment with anti-IL17A or IL-17 receptor antibodies, respectively, predisposes patients to mucocutaneous candidiasis. In psoriasis, aberrant activation of IL-17 signaling on skin keratinocytes results in the secretion of inflammatory cytokines and anti-microbial peptides, such as beta-defensin 2, resulting in neutrophil recruitment and aberrant proliferation and differentiation of skin keratinocytes [7, 8].

While antibodies which target the IL-17 pathway are quite efficacious in psoriasis, there are few oral therapeutic options for patients who require systemic therapy. Oral treatment options include methotrexate, cyclosporine, acitretin, fumaric acid esters, and apremilast. Methotrexate is associated with hepatotoxicity, bone marrow suppression, and rarely pulmonary toxicity. Cyclosporine is associated with renal toxicity. As such, both medications require chronic monitoring limiting long term use. Acitretin is associated with lipid derangements, pancreatitis, hepatotoxicity, and teratogenicity. Fumaric acid esters are associated with flushing and gastrointestinal side effects as well as lower lymphocyte counts and increased risk of PML (progressive multifocal leukoencephalopathy). Apremilast usage is limited due to on target gastrointestinal tolerability issues. These options are less efficacious than antibodies that target the IL-17 pathway and have safety and tolerability concerns.

S011806 is a small molecular inhibitor of IL-17A. S011806 specifically binds to the IL-17AA homodimer and prevents IL-17AA homodimer from binding to the IL-17 receptor complex. In a rat model of rheumatoid arthritis mediated by IL-17, S011806 was able to ameliorate disease to a similar degree as an anti-IL-17 antibody (see Investigator's Brochure for further details). Based on the non-clinical data demonstrating therapeutic improvement in IL-17 mediated disease, S011806 is being developed as a potential oral therapy for the treatment of psoriasis.

2.2 Rationale

The purpose of this first in human study is to evaluate the safety, tolerability, pharmacokinetics (PK), and exploratory pharmacodynamics (PD) of single and repeat oral doses of up to 10 days with S011806 in healthy subjects and repeat oral doses up to 28 days in patients with psoriasis. Exploratory assessments of clinical activity will also be assessed. The intention of this study is to gain an initial assessment of the safety and PK of the molecule as well preliminary activity (PD and clinical activity) in patients with psoriasis.

2.2.1 Rationale for Dose Selection

2.2.1.1 Rationale for starting dose in Part 1 [Single Ascending Dose (SAD)]

The proposed starting single dose in Part 1 healthy subjects is 25mg. This starting dose was selected based on evaluation of all available nonclinical safety and pharmacology data on S011806 (see the S011806 Investigator's Brochure [IB]) as well as clinical data from other IL-17A inhibitors that have been tested in healthy subjects.

Per the Committee for Medicinal Products for Human Use (CHMP) guideline, the starting dose for healthy subjects should be a dose expected to result in an exposure level lower than the pharmacologically active dose (PAD). However, in healthy subjects, the serum level of IL-17A has been reported to be extremely low, ranging from "not detectable" to an average 0.2-2.5 pg/mL level [9, 10]. The baseline serum IL-17A levels are at least one magnitude lower than the concentration of IL-17A necessary to induce inflammatory signalling [11], suggesting that IL-17A signalling pathways are not likely to be active in normal healthy subjects. Consistent with this notion, clinical studies conducted for secukinumab, a fully human monoclonal antibody targeting IL-17A, have evaluated intravenous (IV) dosing of secukinumab at levels up to 10mg/kg and subcutaneous (SC) doses up to 300mg in several clinical studies involving healthy subjects. The maximum concentration (C_{max}) in these studies ranged 237-2638 μg/mL (IV) or 35-40 μg/mL (SC) and were safe and well tolerated. The exposure (C_{max}) levels in these healthy subject studies are several orders of magnitude higher than PAD level of secukinumab defined via in vitro pharmacology experiments where the IC_{50} for blocking IL-17A binding to its receptor was shown at 0.51+/-0.01 nM level [12]. Hence, the rationale of using a PAD for an IL-17A inhibitor is not expected to be relevant to healthy subjects.

Another method to calculate a safe starting dose is the maximum recommended starting dose (MRSD) approach, described in a Food and Drug Administration (FDA) guideline, "Estimating the Maximum Safe Starting Dose in Initial Clinical Trials for Therapeutics in Adult Healthy Volunteers" [13]. The MRSD is based on toxicology study NOAEL (No Observable Adverse Effect Level) exposure levels and is designed to avoid toxicity in the initial clinical dose while simultaneously enabling reasonably rapid attainment of the

Phase I study objectives (e.g., assessment of compound tolerability, PD, or PK profile). Utilizing this guidance, the most sensitive and relevant nonclinical species for S011806 safety assessment is dog (as compared with rat) (see section 4.4 of S011806 IB). Since at the NOAEL dose level of 300mg/kg/day no adverse findings were observed in the 4-week GLP dog toxicity study, exposures observed at this dose level were used as safe/tolerated exposure references. Applying the algorithm defined in FDA guidance, the human equivalent dose (HED) of the dog NOAEL (300mg/kg/d) is approximately 10,000 mg (assuming a 60kg median body weight) [14], and a default safety factor of 10 is applied to derive a MRSD of 1000mg. To account for uncertainty around the translatability of nonclinical studies to humans in addition to the uncertainty of human dose projection, a 40-fold safety margin was applied to the MRSD and 25mg was proposed as the starting dose in healthy subjects. The extra 40x safety factor was applied here because the predicted concentration of a 25mg dose will be transiently (covering a couple of hours) higher than the S011806 trough concentrations showing in vivo pharmacology activity in rat collagen-induced arthritis (CIA) models. Nevertheless, the predicted human exposures at a 25mg starting dose (C_{max} =33 ng/mL and $AUC_{0\text{-}inf}$ =364 ng·hr/mL) are associated with a 608- and 205-fold safety margin based on the dog NOAEL exposure levels (C_{max} and AUC based, respectively) (see section 5.2 of the S011806 IB).

2.2.1.2 Rationale for Dose Escalation and Maximum Dose in Part 1 (SAD)

Dose escalations between cohorts are designed to target increasing exposures (up to 3-fold), with each subsequent dose escalation generally having progressively smaller fold increases in exposure targeted. The highest proposed cohort exposure is anticipated to result in a mean cohort C_{max} and $AUC_{0\text{-}inf}$ exposure that targets EC90 of S011806 for more than 90% of subjects (or higher) but is below the HED of the dog NOAEL (<10000mg).

A wide range of dose levels need to be tested in Part 1. The potential indication for S011806 is psoriasis, a type of chronic and recurrent autoimmune inflammatory skin disease. Based on the current treatment paradigm of secukinumab (an anti-IL-17A monoclonal antibody), a loading dose is a well-established method to rapidly achieve a

steady state therapeutic exposure and this involves an initial dose that is substantially higher than doses administered subsequently to maintain steady state concentrations. In addition, subsequent to the First-in-Human study (e.g., in Phase II and beyond), some patients might be exposed to supratherapeutic exposures for a variety of reasons: PK or PD variability, unintentional or intentional overdose, dosing errors, or impaired clearance that may occur with certain diseases. Thus, a careful exploration of higher exposures in healthy subjects (who generally have greater physiologic reserve than patients) at a closely monitored Phase I unit is important to support continued development and effective safety monitoring in future studies. Furthermore, International Council for Harmonisation (ICH) E14 states that "An adequate drug development programme should ensure that the dose-response and generally the concentration-response relationship for QT/QTc prolongation have been characterized, including exploration of concentrations that are higher than those achieved following the anticipated therapeutic doses." Hence to best evaluate concentration-QTc relationship (planned as part of the safety assessment in this Phase I study), a wide dose range is planned for evaluation. Based on exposure-safety data from 4-week GLP dog toxicology studies and predicted human exposures using projected human PK parameters, all proposed Part 1 doses and exposure levels are expected to be safe and adequately tolerated (Table 1).

Table 1. Planned S011806 Doses in Part 1 (SAD) Cohorts

Planned Doses* (mg)	NOAEL Coverage (Safety Factor)	
	C_{max} based	AUC based
25	608 x	205 x
75	203 x	68 x
225	68 x	23 x
450	34 x	11.5 x
800	19 x	6.4 x

*: Planned dose escalation scheme, actual dose level may change based on available human PK and safety data prior to the escalation but will not exceed 3-fold escalation and a maximum of 800mg.

As an additional safety measure, Part 1 will utilize sentinel dosing to reduce the risk of simultaneously exposing all subjects in a cohort to exposures that have not been previously tested. Two subjects (1 active, 1 placebo) in each cohort will be dosed prior to all other subjects in the cohort and will be observed for a minimum of 24 hours. If there are no safety concerns, the remainder of the cohort will then be dosed.

2.2.1.3 Rationale for Dose Levels and Maximum Dose in Part 2 [Multiple Ascending Dose (MAD)]

Dose levels in Part 2 of the study will be chosen based on emerging PK and safety data from Part 1 and Part 2. Dose levels in Part 2 will explore exposures (up to 3-fold exposure increases between cohorts) in the anticipated therapeutic dose range for future clinical studies, as extrapolated from comparisons of S011806 with an anti-IL17A antibody in the rat CIA model. In this model, treatment with S011806 demonstrated dose/exposure-dependent improvement in disease outcomes that were similar to the effect of an anti-IL-17A antibody. The dose levels and associated drug concentration levels evaluated in this CIA model provided good insights in defining a clinically relevant dose range for potential therapeutic benefit. The starting dose in Part 2 will target a steady state trough concentration (SS C_{trough}) approximately similar to the SS C_{trough} of the lowest dose (100mg/kg QD or 50mg/kg BID) in the rat CIA model that demonstrated improved disease activity vs. the control group (average SS C_{trough} (mean±SD) are 7±8, and 36±21 ng/mL for QD and BID regimens, respectively, see S011806 IB Section 4.4.2). A human dose range of 100 mg QD to 800 mg BID is predicted to achieve SS C_{trough} observed from the lowest to the highest efficacious doses from these in vivo studies. Given the uncertainty and variability of predicted human PK profiles, the unknown translatability of exposure-response relationship from a rat CIA model to psoriasis patients, and emerging PK and safety data, more frequent dosing (e.g. BID or TID) may be evaluated in Part 2 to reach the predicted target efficacious exposure range (SS C_{trough}). The dose tested in Part 2 can only be started after a higher dose in Part 1 has been tested and deemed safe. Subsequent dose levels in Part 2 will target approximately 2-3-fold increases in exposure levels and can only be started after previous Part 2 cohorts have completed and been deemed safe. As an additional safety measure, Part 2 will utilize sentinel dosing to reduce

the risk of simultaneously exposing all subjects in a cohort to exposures that have not been previously tested.

2.2.1.4 Rationale for Dose Levels in the Part 3 Cohorts in Psoriasis Patients

The key objectives of Part 3 of the study are to understand safety, tolerability and PK of repeat dosing of S011806 in psoriasis patients. In addition, Part 3 will also explore the pharmacodynamics and clinical activity of S011806 in psoriasis patients. The selected dose regimens aim to provide steady state trough concentrations at or greater than the levels required to provide therapeutic activity comparable to an anti-IL17A antibody in the rat CIA model. In addition, based on rat CIA model experiments, the BID regimen trended towards better efficacy than the QD regimen at the same total daily dose, indicating that higher C_{trough} concentrations appear to drive better efficacy as compared with higher total AUC. Based on these in vivo pharmacology experiment outcomes, at least one BID regimen is planned in Part 3 of the Phase I study. Actual dose regimens in Part 3 will be determined based on the assessment and evaluation of safety, tolerability, and pharmacokinetics data from Part 1 and Part 2 cohorts but will not exceed the highest tested dose in Parts 1 and 2.

2.3 Benefit-Risk Assessment

The benefit-risk assessment of S011806 is based on the clinical experience of inhibition of IL-17 via secukinumab which has been extensively studied in patients and healthy subjects and data from non-clinical studies of S011806. Per the secukinumab SmPC, upper respiratory infections were very common adverse reactions. Common adverse reactions include tinea pedis, oral herpes, headache, nausea, diarrhea, rhinorrhea, and fatigue. To ensure safety, subjects and patients with active infections, hepatitis B, HIV, hepatitis B and C, and TB will be excluded. While uncommon, worsening of inflammatory bowel disease can occur with treatment with secukinumab, thus, patients with a history of inflammatory bowel disease will be excluded and adverse events of inflammatory bowel disease will be considered an adverse event of special interest (Section 8.11.6).

Results in rat and dog 28-day GLP toxicity studies with S011806 identified potential clinical safety concerns. The first was dose-dependent general tolerability, including emesis (dog only), decreased appetite, and weight loss. Additionally, a reversible dose-dependent increase in liver size and weight with microscopic findings of hepatocellular hypertrophy and minimal increases in serum liver functional measures in individual animals were noted in both rats and dogs. These findings were considered adaptive rather than adverse and are not likely to translate to humans. Finally, reversible moderate renal tubular degeneration/regeneration, possibly an exacerbation of chronic progressive nephropathy, a common incidental finding in rats, was noted in male rats only at the highest dose level of 450 mg/kg/day in the 28-day GLP toxicity study. All of these safety findings were dose-dependent, fully or partially reversible, and all are clinically monitorable.

For Parts 1 and 2, the safety and pharmacokinetics of S011806 will be studied in healthy subjects. As these are healthy subjects, there is no anticipated benefit during Parts 1 and 2. Healthy subjects will be included to minimize potential confounding effects of disease in psoriasis patient population and potential concomitant medications. The potential risks associated with S011806 can be appropriately mitigated by the careful selection of study subjects and the proposed safety monitoring procedures. As such, the risk to potential subjects is considered low.

Routine safety and tolerability will be evaluated from reported adverse events (AEs), physical examinations, vital sign measurements, cardiac Holter monitoring, 12-lead ECGs, C-SSRS (Parts 2 and 3) and clinical laboratory test results as well as continued observation by clinical staff. The study will be conducted in a Phase 1 unit with immediate access to hospital facilities for the treatment of medical emergencies. In-house observation during Parts 1 and 2 as detailed in the SOA will allow for continuous medical monitoring for all subjects following the first dose in each treatment group. Given the projected human elimination half life is 4.5 hours, subjects will only be discharged from the unit 48 hours (minimum of 5 half-lives) after the last dose and the Investigator deems it safe to do so. Residence at the CRU post-dose may be adjusted based on emerging

data (minimum of 5 half-lives post dose). These proposed measures will minimize risk to subjects participating in this study. The potential risks identified in association with S011806 are justified by the anticipated benefits that may be afforded by the future development of a new therapy in an area of unmet need.

In Part 3, as inhibition of IL-17A with secukinumab has demonstrated benefit in patients with psoriasis, S011806 may improve disease activity in patients with psoriasis. Part 3 is primarily designed to understand the safety, pharmacokinetics, and pharmacodynamics of multiple dose regimens (up to 28 days) of S011806 in psoriasis patients and to explore initial signs of therapeutic activity. Safety and tolerability will be evaluated from reported AEs, physical examinations, vital sign measurements, 12-lead ECGs, C-SSRS and clinical laboratory test results as per the SOA. Exclusion criteria in psoriasis patients have also been added to mitigate the known risks of inhibiting IL-17A signaling as described above.

Given the dynamic, ongoing global health crisis related to COVID-19, the risk to subjects and patients will be assessed and addressed on an ongoing basis. Risk mitigation elements included in this protocol include a COVID-19 questionnaire and clinical assessment at each visit prior to any contact with study staff or other subjects/patients. A reverse transcription polymerase chain reaction or lateral flow test for active SARSCoV-2 will be conducted prior to the first IMP administration. Lateral flow testing will also be performed on specific days in accordance with local site policies. The CRU staff will follow locally established risk mitigation processes to minimize exposure to COVID-19 for subjects and staff. Any subjects or patients testing positive for active COVID-19 during the study will receive the appropriate medical care, and precautions, according to local requirement, will be taken to ensure the safety of other subjects, patients and research staff. Any additional policies or information will be assessed on an ongoing basis and integrated into study conduct, as appropriate. Should the patient be unable to attend the unit due to COVID-19 restrictions, safety follow-up and collection of data will be done by phone or e-mail.

While there is an increased risk of upper respiratory infections and nasopharyngitis in patients who are treated with anti-IL-17A antibodies, there is limited experience with the effects of inhibiting the IL-17A pathway and COVID-19 infections. Evidence to date suggests no increased risk of COVID-19 infections and potential benefit of treatment in limited studies attributed to the contribution of IL-17A to the cytokine storm of COVID-19 ARDS. In two population-based cohort study of 680 patients and 4200 patients treated with IL-17 inhibitors, there was no increased risk of infection or complications of COVID-19 [19, 20]. In a retrospective case control study, the IL-17A inhibitor netakimab improved the inflammatory response and need for oxygenation but did not improve the need for mechanical ventilation and mortality [21].

3. Objectives and Endpoints

3.1 Primary Objectives (Safety)

3.1.1 Part 1

- To assess the safety and tolerability of a single ascending oral dose of S011806 in healthy subjects.

3.1.2 Part 2

- To assess the safety and tolerability of multiple ascending oral doses of S011806 in healthy subjects.

3.1.3 Part 3

- To assess the safety and tolerability of multiple oral doses of S011806 in patients with psoriasis.

3.2 Secondary objectives (Plasma PK of S011806)

3.2.1 Part 1

- To characterise systemic PK of S011806 after a single ascending oral dose in healthy subjects.
- To determine the effect of food on a single oral dose of S011806 in healthy subjects.

3.2.2 Part 2

- To characterise systemic PK of S011806 after multiple ascending oral doses in healthy subjects.

3.2.3 Part 3

- To evaluate systemic PK of S011806 after multiple oral doses in patients with psoriasis.

3.3 Exploratory Objectives

3.3.1 Part 1

- To potentially conduct preliminary metabolite profiling and characterisation of S011806 in healthy subjects with human plasma in selected cohorts.

3.3.2 Part 2

- To assess the renal elimination of S011806 in healthy subjects following oral administration of multiple ascending doses of S011806 in selected cohorts
- To conduct preliminary metabolite profiling and characterisation of S011806 in healthy subjects with human plasma and/or urine in selected cohorts.

3.3.3 Part 3

- To assess exploratory PD endpoints following multiple oral doses of S011806 in patients with psoriasis.
- To explore preliminary clinical activity of S011806 in psoriasis patients after 4-week treatment.
- To explore potential drug exposure-response relationship based on, but not limited to, the PASI score.

3.3.4 Estimands

As the primary objectives of this study are safety and tolerability, and the planned summaries are descriptive in nature, estimand statements per the estimand framework (ICH E9 (R1) addendum) will not be implemented.

3.4 Endpoints

3.4.1 Primary Endpoints (Safety)

Safety and tolerability will be assessed by monitoring of AEs, physical examinations, C-SSRS, changes in vital signs, clinical laboratory parameters, cardiac Holter monitoring and ECGs.

3.4.2 Secondary Endpoints (Plasma PK of S011806)

Quantification of S011806 in plasma will be performed using High-performance Liquid Chromatography with tandem mass spectrometry (LC-MS/MS) method followed by calculation of pharmacokinetic parameters, including but not limited to:

In Parts 1 and 2, calculation of pharmacokinetic parameters, including but not limited to; C_{max}, T_{max}, AUC and $t_{1/2}$.

In addition, in the Part 1 food effect cohort, relative bioavailability of S011806 following a high-fat breakfast versus fasted state based on AUC_{inf} and C_{max} will be assessed.

In Part 3, observed C_{max} on Days 1 and 22, trough concentrations on Days 2, 8, 15, 22 and 29 will be reported.

Dose and time dependency will be assessed for the pharmacokinetic parameters.

Further details regarding specific PK parameters to be calculated are included in Section 9.3.3

3.4.3 Exploratory Endpoints

Plasma and urine PK samples collected during Part 1 and Part 2 of the study may also be used for exploratory research, including but not limited to, metabolite profiling and identification. In Part 2, calculation of urine PK parameters including but not limited to Ae, Ae_{t1-t2}, Clr and Fe and metabolite analysis (identification) will be assessed as data permit. In Part 3, Exploratory PD biomarkers will include, but not be limited to, levels of inflammatory cytokines (e.g. IL-17A) and beta defensin-2 (BD-2) in serum. Exploratory PD biomarkers will also include but not be limited to changes in histology by hematoxylin and eosin staining and RNA analysis including but not limited to gene expression including IL-17A, F and beta-defensin 2 in skin biopsies. Exploratory clinical activity will

include but not be limited to change in PASI, PGA, BSA and LSS assessment. Clinical activity will also include evaluation of data from photography of lesion sites as data permit. Potential drug exposure-response relationship based on, but not limited to, the PASI score will also be assessed.

Further details are included in Section 8.14.

4. Study Design

4.1 Overall Study Design

This is an integrated Phase 1, 3-part, double-blinded, randomized, placebo-controlled SAD/SAD Food-effect (Part 1) and MAD (Part 2) study in healthy subjects and multiple dose study in patients with psoriasis (Part 3). Safety, tolerability, and PK will be assessed following either single ascending (SAD, Part 1) or multiple ascending (MAD, Part 2) oral dosing of S011806 in a tablet formulation. In Part 3, safety, tolerability, PK, exploratory PD and clinical activity will be assessed following once, twice or three times daily oral doses of S011806 in tablet formulation. Approximately 80 healthy subjects and up to 42 patients with psoriasis will be enrolled (48 in Part 1; 32 in Part 2 and up to 42 in Part 3).

Healthy subjects and patients with psoriasis will be screened for eligibility to participate in the study within 28 days before dosing (Day 1). In Parts 1 and 2, subjects will be admitted to the CRU on the evening of Day -1 and remain resident until Day 3 (Part 1), Day 9 (Part 2, if 7-day dosing), or Day 12 (Part 2, if 10-day dosing) and return for follow-up 7 (±2) days following final IMP dose. Patients in Part 3 will be resident overnight from Day 1 to Day 2 then for subsequent visits will attend as outpatients in the CRU as indicated in Section 1.2.8 from Day 3 to Day 43 (± 2 days).

Subjects or patients will participate in only one cohort and in only Part 1, 2 or 3. Further details of each part of the study are described in Sections 4.2, 4.3 and 4.4.

Figure 1 presents an overview of study design. A schedule of activities (SOA) for Part 1, Part 1 food-effect cohort, Part 2, and Part 3 are provided in SOA Table in Section 1.2.

Figure 1. Overall Study Design and Dose-escalation Scheme

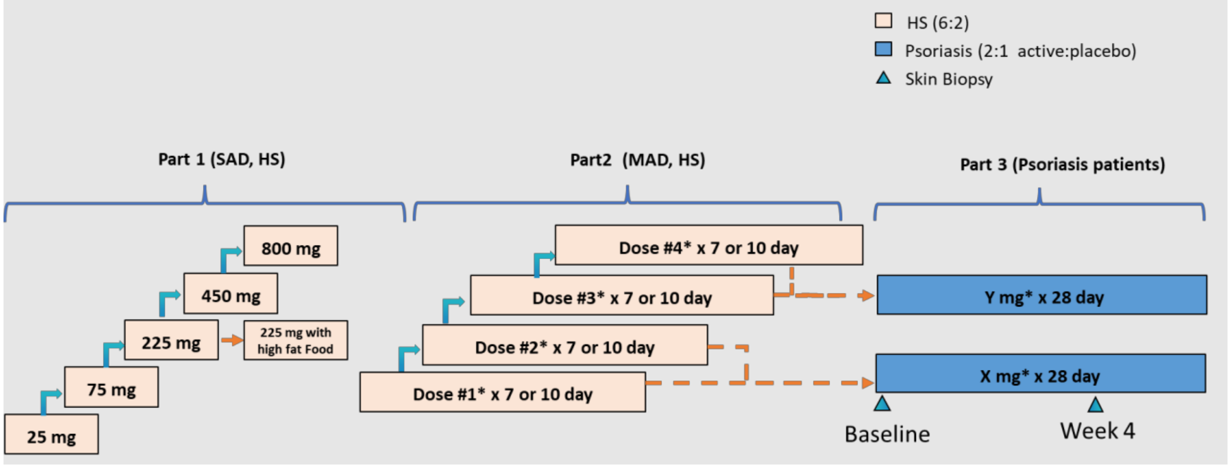

HS: Healthy subjects, SAD: single ascending dose, MAD: Multiple-ascending dose,
*Based on Part 1 and/or Part 2 PK data, if available, the dose frequency may be once daily (QD), twice daily (BID) or three times daily (TID), but will not exceed the highest tolerated dose in Part 1.

4.2 Part 1 (Healthy Subject SAD)

Part 1 is a single ascending dose (SAD) study to assess the safety, tolerability, PK and food-effect of single oral doses of S011806 in up to 5 cohorts of healthy male and female subjects. Each cohort will comprise of approximately 8 subjects (6:2 active: placebo) for a planned total of up to 40 subjects. An evaluable subject for Part 1 is defined as a subject who has received a dose of IMP and has sufficient data for dose escalation review and final analysis (i.e. evaluation of the safety and PK objectives) as outlined in Sections 6.6 and Section 3.4.1 and 3.4.2.

In each cohort, subjects will receive a single oral dose of S011806 or placebo on the morning of Day 1 with approximately 240ml water. Subjects will be required to fast (water permitted) for at least 10 hours prior to and 4 hours post dose. One cohort, the food-effect cohort, will return for a second treatment period as detailed in Section 4.2.1. Each cohort, including both treatment periods in the food-effect cohort, will include a sentinel group of 2 subjects (1:1, active:placebo) who will be dosed at least 24 hours prior to dosing the remaining 6 subjects (5:1, active:placebo). The remaining subjects will only be dosed if

the sentinel group shows no clinically significant safety or tolerability concerns at the discretion of the Investigator (e.g. review of AE's, vital signs and ECGs).
The planned starting dose of S011806 for Cohort 1 is 25mg administered orally. Subsequent doses will be determined from the review of safety and available PK data by the SAC from the preceding cohorts. The time between cohorts will be sufficient to allow this review. The data reviews will be conducted by the SAC, which will comprise the Principal Investigator (or delegate) and the Sponsor's physician (or delegate) as a minimum. Full details of the interim data reviews, including dose escalation criteria, are provided in Section 6.6. Planned doses are listed in Section 2.2.1.1 and may be modified based on available safety and PK data. Modified doses will not exceed a 3-fold escalation and a maximum of 800mg.

Subjects will undergo safety and tolerability assessments (e.g. physical examinations, vital signs, 12-lead ECG, cardiac Holter monitoring, clinical laboratory tests, and AE monitoring), and PK evaluations at specified time points during the study (see Section 1.2.1 and 1.2.3). Subjects will remain resident at the CRU until Day 3 after completion of safety assessments at 48 hours post-dose and providing there are no safety concerns, they will be discharged from the unit. Residence at the CRU post-dose may be adjusted based on emerging data (minimum of 5 half-lives post dose). They will return to the CRU on Day 8 (± 2 days) for safety evaluations to be performed as detailed in Section 1.2.1.

4.2.1 Part 1 (Healthy Subject SAD): Food-Effect Cohort

After the first cohort is completed in Part 1, one cohort will be dedicated to evaluating a potential food effect on the PK of S011806. Although planned to occur in Cohort 3, the dose of the food-effect cohort will be selected based on emerging safety and PK data. The predicted exposure in the fed state at the selected dose should not exceed a dose/exposure as defined in the PK stopping criteria (Table 3) and must be agreed as acceptable by the safety advisory committee (SAC). Following administration of S011806 in the fasted state, the same cohort of subjects will be re-dosed after IMP washout (i.e. at least 5 half-lives of S011806 or 7 days, whichever is longer), in the fed state after a high fat meal (See Section 6.2 and Study Reference Manual for further details). After the food-

effect cohort has been dosed and the effect of food on PK and safety has been characterized, subsequent cohorts in Parts 2, and 3 may be administered either with or without food. The food-effect cohort may be run in parallel with the other ascending cohorts. Subjects in the food-effect cohort will undergo the same assessments at the same time points in Treatment Period 2 as they did in Treatment Period 1 as detailed in Section 1.2.2. The cohort size may be adjusted to ensure at least 6 subjects complete the residential visit assessments.

4.3 Part 2 (Healthy Subject MAD)

Part 2 is a multiple ascending dose (MAD) study to assess the safety, tolerability, and PK of multiple oral doses of S011806 in up to 4 cohorts of healthy male and female subjects. Each cohort will comprise approximately 8 subjects (6:2, active:placebo) with planned total of approximately 32 subjects receiving active drug or placebo once, twice or three times daily for 7 or 10 days. If emerging PK data from Part 1 suggest that steady-state or 5 half-lives can be attained within 5 days of the dose, the dosing duration of Part 2 cohorts may be decreased to 7 days. An evaluable subject for Part 2 of the study is defined as a subject who has received all planned doses of IMP and has sufficient data for dose escalation review and final analysis (i.e. evaluation of the safety and PK objectives) as outlined in Sections 6.6 and Section 3.4.1 and 3.4.2.

Each cohort will include a sentinel group of 2 subjects (1:1, active:placebo) who will be dosed at least 3 days prior to the remaining 6 subjects (i.e., 24 hours after the morning dose on Day 3), or longer if needed based on PK data. The remaining subjects will only be dosed if the sentinel group shows no clinically significant safety or tolerability concerns at the discretion of the Investigator (e.g. review of AE's, vital signs, 12-lead ECGs, C-SSRS and available clinical laboratory tests).

The planned level, frequency, and duration of S011806 doses to be administered in Part 2 will be confirmed during the interim review of safety and available PK data from Part 1 cohorts. Part 2 cohorts may be initiated in parallel with the conduct of the Part 1 cohorts once it has been determined by the SAC that adequate safety and exposure data have

been generated after completion of dosing of a Part 1 cohort one dose level higher than the planned first dose in Part 2. No Part 2 dose will exceed the maximum dose studied in Part 1. There will be an interim data review before each dose escalation to the next dose level to determine safety and tolerability. The time between cohorts will be sufficient to allow this review. The data reviews will be conducted by the SAC, which will comprise the Principal Investigator (or delegate) and the Sponsor's physician (or delegate) as a minimum. Full details of the interim data reviews, including dose escalation criteria, are provided in Section 6.6.

Fasting requirements and dosing instructions are detailed in Section 6.2. Following the first dose of IMP (Day 1), subjects will remain resident in the CRU until completion of safety assessments at 48 hours post final dose (Day 9 for 7-day dosing or Day 12 for 10-day dosing). Residence at the CRU after dose may be adjusted based on emerging data (minimum of 5 half-lives after final dose). In the event of twice daily dosing, evening doses will be administered approximately 12 hours (±1 hour) after the morning dose. In the event of three times daily dosing, subjects will be dosed approximately 8 hours apart from the earlier dose. Subjects will undergo safety and tolerability (i.e., physical examinations, vital signs, 12-lead ECG, cardiac Holter monitoring, clinical laboratory tests, C-SSRS and AE monitoring), and PK evaluations at specified time points during this time (see Section 1.2.4, 1.2.5, 1.2.6 and 1.2.7). Providing there are no safety concerns, they will be discharged from the unit and return to the CRU for safety evaluations to be performed as detailed in Section 1.2.4 and 1.2.5 on Day 14 (± 2 days) or 17 (± 2 days), for 7- or 10-day dosing respectively.

4.4 Part 3 (Psoriasis Patients)

Part 3 will assess the safety, tolerability, PK, exploratory PD, and preliminary clinical activity of multiple oral doses of S011806 in 2 cohorts of male and female patients with psoriasis (planned total of up to 42 patients). Each cohort will comprise a minimum of approximately 15 patients up to 21 patients, randomised in a 2:1 ratio active:placebo, dosed once, twice or three times daily for 28 days.

The planned level and frequency of S011806 doses to be administered in Part 3 will be

confirmed during the interim review of safety and available PK data from completed Part 1 and 2 cohorts. Two dosing regimens are planned for Part 3, targeting pharmacologically active exposure levels based on exposure-response data from rat in vivo pharmacology study using a CIA model. Actual doses will be determined based on the PK and safety outcomes from both Part 1 and Part 2, and the higher dose will not exceed the highest exposure evaluated in Part 2. Part 3 cohorts may commence in parallel with the conduct of Part 2 cohorts once it has been determined by the SAC that adequate safety and exposure data have been generated from the Part 2 cohorts. As a minimum, Part 3 can commence once the same dose level in Part 2 has been completed and reviewed by the SAC. Part 3 cohort 2 may commence prior to the completion of Part 3 cohort 1 providing the same dose level in Part 2 has been completed and reviewed by the SAC. No single dose will exceed the maximum dose studied in Part 1 or 2.

Fasting requirements and dosing instruction are detailed in Section 6.2. Following the first dose of IMP (Day 1), patients will remain resident in the CRU overnight and until the completion of Day 2 pre-dose procedures and will receive the Day 2 morning dose prior to discharge. Patients will undergo safety and tolerability (i.e., physical examinations, vital signs, 12-lead ECG, C-SSRS, clinical laboratory tests and AE monitoring), PK and PD evaluations at specified time points during this time (see Section 1.2.9). Providing there are no safety concerns, patients will be discharged from the unit and return on Days 8, 15, 22, and 29 for safety, PK, PD and clinical evaluations and IMP administration as detailed in Section 1.2.8 and 1.2.9. On days when patients are not required to attend the CRU, IMP dosing will be completed by the patient at home at the time of the day consistent with the time of the dosing during Day 1. Patients will complete a daily dosing diary during periods of home dosing. Patients will return to the CRU on Day 43 (± 2 days) for safety and PD evaluations to be performed as detailed in Section1.2.8.

In patients treated with anti-IL-17A antibodies, rebound of disease activity did not occur immediately after drug washout. This extended duration (2 week post last dose) of follow-up is intended to gain an initial assessment of duration of pharmacological activity, potential rebound of psoriatic disease activity after stopping treatment, and any potential

delayed safety signal after drug washout to inform future safety monitoring in subsequent clinical studies.

4.5 End of Study

All subjects and patients who receive at least one dose of study drug, including those who terminate the study early, will return to the CRU for follow up procedures and to determine if any new AEs have occurred and/or whether any previously reported AEs have recovered since the last study visit. This will take place 7 (±2) days after the last study drug administration for Parts 1 and 2 and 15 (±2) days after last study drug administration for Part 3.

A subject or patient is considered to have completed the study if they have completed treatment to the end of their assigned cohort and attended their final follow up visit on Day 8 (±2) for Part 1, Day 14 or 17 (±2) for Part 2 (7- or 10-day dosing respectively), or Day 43 (±2) for Part 3.

The end of the study occurs after completion of all of the planned cohorts. The end of the study is defined as the date of the last visit with the last patient in the study. In addition, the Sponsor may decide to terminate the study at any time if appropriate (as described in Section 7).

5. Study Population

Prospective approval of protocol deviations to inclusion and exclusion criteria, also known as protocol waivers or exemptions, is not permitted.

5.1 Inclusion Criteria

5.1.1 Inclusion Criteria for Healthy Subjects (Parts 1 and 2)

Subjects must meet all of the following inclusion criteria to be eligible to participate in the study:

1. Subjects must have a written informed consent obtained prior to any study-related procedures.
2. Subjects must be able to understand and comply with the requirements of the study, as judged by the Investigator or designee.
3. Men and women subjects must be between 18-55 years inclusive, at the time of informed consent.
4. Female subjects must either be of non-childbearing potential or if of childbearing potential, must not be pregnant, breast feeding or lactating and use a highly effective birth control method during treatment and for 90 days following last administered dose (see Section 10.4). In addition, male partners of female subjects of childbearing potential must use highly effective contraception for 90 days following the last administered dose (see Section 10.4)
5. Male subjects who are sexually active with a partner of childbearing potential must use, with their partner, a condom plus an approved method of highly effective contraception from the time of informed consent until 90 days after their last dose of IMP (see Section 10.4).
6. Subjects must agree not to donate semen or ova/oocytes during the study and for 90 days after the last dose of IMP (see Section 10.4).
7. Subjects must have a body mass index (BMI) ≥ 18 and ≤ 35 kg/m^2.
8. Subjects must be in good health as determined by medical history, physical examination, vital signs, 12-lead ECG and clinical laboratory assessments at the time of screening, as judged by the Investigator or designee.

5.1.2 Inclusion Criteria for Patients with Psoriasis (Part 3)

Patients must meet all of the following inclusion criteria to be eligible to participate in the study:

1. Patients must have a written informed consent obtained prior to any study-related procedures.
2. Patients must be able to understand and comply with the requirements of the study, as judged by the Investigator or designee.
3. Men and women patients must be between 18-65 years inclusive, at the time of informed consent.
4. Patients must have a documented diagnosis of plaque psoriasis for ≥ 6 months prior to screening.
5. Physicians Global Assessment (PGA) of 2/3 i.e. mild or moderate plaque psoriasis at baseline.
6. Body Surface Area (BSA) ≥3% at baseline.
7. A minimum of 2 psoriatic lesions of at least 2 cm x 2 cm at baseline, with at least 1 plaque in a site suitable for biopsy.
8. Female patients must either be of non-childbearing potential or if of childbearing potential, must not be pregnant, breast feeding or lactating and use a highly effective birth control method during treatment and for 90 days following last administered dose (see Section 10.4). In addition, male partners of female subjects of childbearing potential must use highly effective contraception for 90 days following the last administered dose (see Section 10.4).
9. Male patients who are sexually active with a partner of childbearing potential must use, with their partner, a condom plus an approved method of highly effective contraception from the time of informed consent until 90 days after their last dose of IMP (see Section 10.4).
10. Patients must agree not to donate semen or ova/oocytes during the study and for 90 days after the last dose of IMP (see Section 10.4).
11. Patients must have a body mass index (BMI) ≥ 18 and ≤ 36 kg/m^2.
12. Patients must be in good health as determined by medical history, physical examination, vital signs, 12-lead ECG and clinical laboratory assessments at the

time of screening, as judged by the Investigator or designee.

5.2 Exclusion Criteria

5.2.1 Exclusion Criteria for Healthy Subjects (Parts 1 and 2)

Subjects are excluded from the study if any of the following apply:

1. History or presence of any clinically relevant acute or chronic medical or psychiatric condition that could interfere with the subject's safety during the clinical study or expose the subject to undue risk as judged by the Investigator or designee.
2. After 10 minutes supine rest at the time of screening or prior to dosing on Day 1, any vital signs values outside the following ranges:
   - Systolic blood pressure <90 or >150 mmHg, or
   - Diastolic blood pressure <50 or >95 mmHg, or
   - Pulse <40 or >90 bpm
3. Any clinically significant abnormalities in resting ECG at the time of screening or pre-dose Day 1 including prolonged QTcF (>450 ms for males; >470 ms for females using the mean of triplicate ECG's) and cardiac arrhythmias, as judged by the Investigator or designee.
4. Clinically significant abnormalities in renal function:
   - eGFR <60 mL/min
5. Clinically significant abnormalities in liver function:
   - Bilirubin >1.5 x ULN
   - Aminotransferases >1.5 x ULN
   - ALP >1.5 x ULN
6. Any clinically significant illness, medical/surgical procedure, or trauma within 4 weeks of the first administration of IMP (Day 1).
7. Malignancy within the past 5 years of screening with the exception of in situ removal of basal cell carcinoma, resected benign colonic polyps, or adequately treated cervix carcinoma in-situ.
8. Any planned major surgery within the duration of the study or in the 30 days following study completion.

9. History of latent TB, active tuberculosis, or a positive QuantiFERON® TB Gold result at screening. Patients with an indeterminate QuantiFERON® TB Gold result at screening will be allowed one retest; if not negative on retesting, the subject will be excluded.
10. Females who are pregnant, breast feeding or plan to be pregnant during the study period or 90 days after.
11. Female subjects with a positive serum or urine pregnancy test (minimum sensitivity 25 IU/L or equivalent units of human chorionic gonadotropin [hCG]) at screening or within 24 h prior to the first administration of IMP.
12. Positive serum hepatitis B surface antigen (HBsAg), hepatitis C virus antibodies (HCV Ab) or human immunodeficiency virus (HIV) 1 and/or 2 antibodies at screening.
13. A positive test for active COVID -19 tested within 7 days prior to administration of the IMP on Day 1.
14. History of any drug and/or alcohol abuse in the past 2 years prior to screening.
15. Regular alcohol consumption in males >21 units per week and females >14 units per week.
16. Positive urine drugs of abuse test and/or alcohol breath test at screening or on admission to the unit prior to administration of the IMP on Day -1 that cannot be accounted for by concomitant medication in the opinion of the Investigator or designee.
17. Current or previous use of tobacco, nicotine products, or e-cigarettes in the past 6 months.
18. Smoking history of > 5 pack years.
19. Positive urine cotinine test at screening or Day -1.
20. Receiving any of the prohibited concomitant medications as specified in Section 5.5.4.
21. Any clinically significant infection requiring antimicrobial treatment in the 2 weeks prior to Day 1.
22. Inoculated with a live vaccine in the 28 days prior to Day 1.
23. Has received oral antibiotics within 6 weeks prior to screening.
24. Has received with systemic steroids within 4 weeks prior to screening.
25. Plasma donation within one month of screening or blood donation (or corresponding

blood loss) ≥400ml during the three months prior to screening.

26. Unsuitable veins for multiple venepunctures/cannulation as assessed by the Investigator or designee at screening.
27. Any dietary restrictions incompatible with the diet that can be provided by the study site, in the opinion of the Investigator, or is unwilling to refrain from consuming restricted foods and beverages during the study as defined in Section 5.5.7.1.
28. Regular excessive caffeine consumption defined by a daily intake of >5 cups of caffeine containing beverages.
29. Known history of intolerance or hypersensitivity to S011806 or to any other component of the formulation.
30. Known history of intolerance to the placebo or excipients.
31. Clinically significant serious adverse reaction, allergy or serious hypersensitivity, including to any drug or food, as judged by the Investigator or designee.
32. Involvement in the planning and conduct of the study.
33. Participation in another clinical study with an experimental drug within 3 months or 5 half-lives, whichever is longer, before the administration of IMP.
34. Considered unsuitable for entry into the study in any other way at the discretion of the Principal Investigator or designee, e.g. Investigator considers the subject unlikely to comply with study procedures, restrictions, and requirements.
35. Part 2 Only: Presence of active suicidal ideation or positive suicide behaviour using the "Baseline/Screening" version of the Columbia Suicide Severity Rating Scale (C-SSRS) and with either of the following criteria:
    - History of a suicide attempt within the 5 years prior to the Screening Visit. Subjects with a history of a suicide attempt more than 5 years ago should be evaluated by a mental healthcare professional (eg, locally licensed psychiatrist, psychologist, or master’s level therapist) before enrolling into the study.
    - Suicidal ideation in the past month prior to the Screening Visit as indicated by a positive response ("Yes") to either Question 4 or Question 5 of the "Baseline/Screening" version of the C-SSRS.

5.2.2 Exclusion Criteria for Patients with Psoriasis (Part 3)

Patients are excluded from the study if any of the following apply:

1. History or presence of any clinically relevant acute or chronic medical or psychiatric condition other than psoriasis that could interfere with the patient's safety during the clinical study or expose the patient to undue risk as judged by the Investigator or designee.
2. A diagnosis of non-plaque psoriasis.
3. Plaque psoriasis restricted to the scalp, palms, soles and face.
4. Pustular, erythrodermic, inverse, and guttate psoriasis
5. Drug-induced psoriasis (i.e., new onset or current exacerbation from beta-blockers, calcium channel inhibitors or lithium)
6. Diagnosis of psoriatic arthritis, uveitis, inflammatory bowel disease, or other immune-mediated conditions that are commonly associated with psoriasis for which a subject requires current systemic (oral, SC, or IV) (including corticosteroids, immunosuppressants, biologics) immunosuppressant medical treatment
7. Presence of other skin conditions that could interfere with psoriasis evaluation or assessments as judged by the Investigator or designee.
8. History or presence of inflammatory bowel disease.
9. Presence of active suicidal ideation, or positive suicide behaviour using the "Baseline/Screening" version of the Columbia Suicide Severity Rating Scale (C-SSRS) and with either of the following criteria:
    - History of a suicide attempt within the 5 years prior to the Screening Visit. Subjects with a history of a suicide attempt more than 5 years ago should be evaluated by a mental healthcare professional (eg, locally licensed psychiatrist, psychologist, or master's level therapist) before enrolling into the study.
    - Suicidal ideation in the past month prior to the Screening Visit as indicated by a positive response ("Yes") to either Question 4 or Question 5 of the "Baseline/Screening" version of the C-SSRS.
10. After 10 minutes supine rest at the time of screening or prior to dosing on Day 1, any

vital signs values outside the following ranges:

- Systolic blood pressure <90 or >150 mmHg, or
- Diastolic blood pressure <50 or >95 mmHg, or
- Pulse <40 or >90 bpm

11. Any clinically significant abnormalities in resting ECG at the time of screening or pre-dose Day 1 including prolonged QTcF (>450 ms for males; >470 ms for females using the mean of triplicate ECG's) and cardiac arrhythmias, as judged by the Investigator or designee.
12. Clinically significant abnormalities in renal function:
    - eGFR <60 mL/min
13. Clinically significant abnormalities in liver function:
    - Bilirubin >1.5 x ULN
    - Aminotransferases >1.5 x ULN
    - ALP >1.5 x ULN
14. Any clinically significant illness, medical/surgical procedure or trauma within 4 weeks of the first administration of IMP (Day 1).
15. Significant history of invasive candidiasis or recurrent mucocutaneous candida infections.
16. Malignancy within the past 5 years of screening with the exception of in-situ removal of basal cell carcinoma, resected benign colonic polyps or adequately treated cervix carcinoma in-situ.
17. Any planned major surgery within the duration of the study or in the 30 days following study completion.
18. History of latent TB, active tuberculosis, or a positive QuantiFERON® TB Gold result at screening. Patients with an indeterminate QuantiFERON® TB Gold result at screening will be allowed one retest; if not negative on retesting, the patient will be excluded.
19. Females who are pregnant, breast feeding or plan to be pregnant during the study period or 90 days after.
20. Female patients with a positive serum or urine pregnancy test (minimum sensitivity 25 IU/L or equivalent units of human chorionic gonadotropin [hCG]) at screening or within

24 h prior to the first administration of IMP.

21. Positive serum hepatitis B surface antigen (HBsAg), hepatitis C virus antibodies (HCVAb) or human immunodeficiency virus (HIV) 1 and/or 2 antibodies at screening.
22. A positive test for active COVID -19 tested within 7 days prior to administration of the IMP on Day 1.
23. History of any drug and/or alcohol abuse in the past 2 years prior to screening.
24. Regular alcohol consumption in males >21 units per week and females >14 units per week.
25. Positive urine drugs of abuse test and/or alcohol breath test at screening or prior to administration of the IMP on Day 1 that cannot be accounted for by concomitant medication in the opinion of the Investigator or designee.
26. Treatment with biologics within 5 half-lives (if known) or 6 months prior to dosing on Day 1, whichever is longer
27. Has received any systemic immunosuppressants (e.g. Methotrexate, cyclosporine, hydroxyurea, and tacrolimus) or anakinra within 4 weeks of the first administration of study medication.
28. Has received phototherapy or any systemic medications/treatments that could affect psoriasis or PGA evaluation (including, but not limited to, oral or injectable corticosteroids, retinoids, psoralens, sulfasalazine, hydroxyurea, apremilast, or fumaric acid derivatives) within 4 weeks of the first administration of study medication
29. Has used topical medications/treatments that could affect psoriasis or PGA evaluation (including, but not limited to, corticosteroids, anthralin, calcipotriene, topical vitamin D derivatives, retinoids, tazarotene, picrolimus, and tacrolimus) within 2 weeks of the first administration of study medication.
30. Has received oral antibiotics within 6 weeks prior to screening.
31. Receiving any of the prohibited concomitant medications as specified in Section 5.5.5.
32. Any clinically significant infection requiring antimicrobial treatment in the 2 weeks prior to Day 1.
33. Inoculated with a live vaccine in the 28 days prior to Day 1.
34. Plasma donation within one month of screening or blood donation (or corresponding blood loss) ≥400ml during the three months prior to screening.

35. Unsuitable veins for multiple venepunctures/cannulation as assessed by the Investigator or designee at screening.
36. Any dietary restrictions incompatible with the diet that can be provided by the study site, in the opinion of the Investigator, or is unwilling to refrain from consuming restricted foods and beverages during the study as defined in Section 5.5.7.2.
37. Regular excessive caffeine consumption defined by a daily intake of >5 cups of caffeine containing beverages.
38. Sun bed use in the 4 weeks prior to screening or planned use prior to the final study visit (follow-up visit).
39. Known history of intolerance or hypersensitivity to S011806 or to any other component of the formulation.
40. Known history of intolerance to placebo or excipients.
41. Clinically significant serious adverse reaction allergy or serious hypersensitivity, including to any drug or food, as judged by the Investigator or designee.
42. Involvement in the planning and conduct of the study.
43. Participation in another clinical study with an experimental drug within 3 months or 5 half-lives, whichever is longer, before the administration of IMP.
44. Considered unsuitable for entry into the study in any other way at the discretion of the Principal Investigator or designee, e.g. Investigator considers the subject unlikely to comply with study procedures, restrictions and requirements.

5.3 Screen Failures

Screen failures are defined as subjects or patients who consent to participate in the clinical study but who do not subsequently receive IMP. There is no requirement to enter screen failed subjects and patients into the eCRF (Electronic Case Report Form).

5.4 Rescreening

Re-screening will be allowed for a subject or patient who has discontinued the study as eligible-but-not-required (i.e. reserves) or could not be dosed within the permitted screening window for any reason, or as a pre-treatment failure (i.e. subject or patient has not been treated); the reason for failure must be temporary and expected to resolve. If re-

screening, the subject or patient must be re-consented. Re-screened subjects and patients should be assigned a new screening number at the time of re-consent.

5.5 Study Restrictions

5.5.1 Prior and Concomitant Medication

Any medications (including prescription medication, OTC medication, vaccines, topical medications, herbal or homeopathic remedies, nutritional supplements) used by a subject or patient will be recorded in the source records. For all subjects, any prior medications taken within 28 days prior to screening and all concomitant medications will be recorded in the eCRF. In addition for psoriasis patients, any prior psoriasis medications or treatment taken within the past 5 years prior to screening and all concomitant medications will be recorded in the eCRF.

5.5.2 Permitted Concomitant Medication for Healthy Subjects (Parts 1 and 2)

The following concomitant medications are permitted:

- Paracetamol in doses up to 2000 mg/day as needed (and as deemed appropriate by the Investigator) for mild/moderate headache or other pain
- Medications listed as part of birth control methods will be allowed (refer to Section 10.4)
- Hormone replacement therapy (HRT)

5.5.3 Permitted Concomitant Medication for Patients with Psoriasis (Part 3)

The following concomitant medications are permitted:

- Paracetamol
- Medications listed as part of birth control methods will be allowed (refer to Section 10.4)
- Hormone replacement therapy (HRT)
- Bland emollients

In addition, other concomitant medications taken at a stable dose for at least 3 months prior to screening which are not listed in Section 5.5.5 may be permitted at the Investigators discretion following discussion with the Medical Monitor.

Other medications considered necessary for a subject or patient's safety and wellbeing may be given at the discretion of the Investigator during the residential or study period. Following consultation with the Sponsor, the Investigator will determine whether or not the subject/patient should continue in the study.

Emergency equipment and drugs will be available within the clinical unit as per current standard procedures. In the event that they are required, their use will be documented.

5.5.4 Prohibited Concomitant Medication for Healthy Subjects (Parts 1 and 2)

With the exception of those listed in Section 5.5.2, the use of any prescribed or non-prescribed medication is prohibited within 14 days of IMP administration until after the last study visit (follow-up visit). This includes:

- NSAIDs (including coxibs and aspirin)
- Topical steroids
- Herbal remedies, vitamin supplements and minerals
- Other over-the counter (OTC) medicines

In addition:

- Treatment with strong CYP3A inhibitors (e.g. itraconazole) or strong CYP3A inducers (e.g. rifampicin) as listed by the Food and Drug Administration [15] within 4 weeks or 5 drug half-lives, whichever is longer, prior to administration of study drug, throughout the study, until after the last study visit (follow-up visit).
- Live vaccines are not permitted within 28 days prior to IMP administration until 28 days after the last study visit (follow-up visit)
- Inactivated vaccines (e.g. for COVID-19 or influenza) are not permitted within 2 weeks prior to IMP administration or during the treatment period until 7 days after

the last study visit (follow-up visit). Where a vaccine comprises 2 doses, this refers to either dose.

- Oral antibiotics are not permitted within 6 weeks prior to screening until after the last study visit (follow-up visit).
- Systemic steroids are not permitted within 4 weeks prior to screening until after the last study visit (follow-up visit).

5.5.5 Prohibited Concomitant Medication for Patients with Psoriasis (Part 3)

Preliminary results on enzyme identification suggested that CYP3A isoforms are likely the primary enzymes responsible for the metabolism of S011806 (See S011806 Investigator's Brochure). Therefore, medications that are inhibitors or inducers of the CYP3A enzymes should be avoided. If the use of these medications is necessary, the risk and benefits should be discussed with the Medical Monitor prior to concomitant administration.

The following concomitant medications are prohibited in Part 3:

- Topical medications/treatments that could affect psoriasis or PGA evaluation (including, but not limited to, corticosteroids, anthralin, calcipotriene, topical vitamin D derivatives, retinoids, tazarotene, picrolimus, and tacrolimus) within 14 days of IMP administration until after the last study visit (follow up visit).
- NSAIDs (including coxibs and aspirin) and anticoagulants within 14 days of IMP administration until after the last study visit (follow up visit).
- Treatment with strong or moderate CYP3A inhibitors (e.g. itraconazole or erythromycin) or strong or moderate CYP3A inducers (e.g. rifampicin) as listed by the Food and Drug Administration [15] within 4 weeks or 5 drug half-lives, whichever is longer, prior to administration of study drug, throughout the study, until after the last study visit (follow-up visit, Day 43).
- Live vaccines are not permitted within 28 days prior to IMP administration until 28 days after the last study visit (follow-up visit).

- Inactivated vaccines (e.g. for COVID-19 or influenza) are not permitted within 2 weeks prior to IMP administration or during the treatment period until 7 days after the last study visit (follow-up visit). Where a vaccine comprises 2 doses, this refers to either dose.
- Oral antibiotics are not permitted within 6 weeks prior to screening until after the last study visit (follow-up visit).
- Systemic steroids are not permitted within 4 weeks prior to screening until after the last study visit (follow-up visit).
- Biologics are not permitted within 5 half-lives (if known) or 6 months prior to IMP administration, whichever is longer until after the last study visit (follow-up visit).
- Systemic immunosuppressants (eg, Methotrexate, cyclosporine, hydroxyurea, and tacrolimus) or anakinra are not permitted within 4 weeks prior to IMP administration until after the last study visit (follow-up visit).
- Phototherapy or any systemic medications/treatments that could affect psoriasis or PGA evaluation (including, but not limited to, oral or injectable corticosteroids, retinoids, psoralens, sulfasalazine, hydroxyurea, apremilast, or fumaric acid derivatives) are not permitted within 4 weeks prior to IMP administration until after the last study visit (follow-up visit).

The Medical Monitor should be contacted if there are any questions regarding concomitant or prior therapy.

5.5.6 Lifestyle Considerations

5.5.7 Meals and Dietary Restrictions

5.5.7.1 Meals and Dietary Restrictions Parts 1 and 2

Xanthine or taurine containing products/beverages: Tea, coffee, and/or energy drinks (e.g. Redbull) are not allowed during the study from 24 hours prior to screening and 24 hours prior to admission on Day -1 until the last study visit (follow-up visit) in all parts. Decaffeinated alternatives to tea and coffee are permitted.

Grapefruit and grapefruit containing products: Consumption of grapefruit and/or grapefruit containing products and Seville oranges is not allowed during the study from 14 days prior to IMP administration until after the last study visit (follow-up visit). Foods and beverages containing other types of oranges are not included in this restriction, nor is marmalade of any kind.

Fasting: Samples for serum chemistry will be obtained following a fast of at least 8 hours (water permitted). See Section 6.2 for further details on fasting prior to IMP administration.

5.5.7.2 Meals and Dietary Restrictions Parts 3

Xanthine or taurine containing products/beverages: Tea, coffee, and/or energy drinks (e.g. Redbull) are not allowed 24 hours prior to screening and 24 hours prior to all CRU visits. Decaffeinated alternatives to tea and coffee are permitted.

Grapefruit and grapefruit containing products: Consumption of grapefruit and/or grapefruit containing products and Seville oranges is not allowed during the study from 14 days prior to IMP administration until after the last study visit (follow-up visit). Foods and beverages containing other types of oranges are not included in this restriction, nor is marmalade of any kind.

Fasting: Instructions for feeding and fasting prior to IMP administration will be determined by Part 1 and 2 PK data as detailed in Section 6.2. Samples for serum chemistry will be obtained following a fast of at least 8 hours (water permitted).

5.5.8 Alcohol Restrictions

Alcohol: Consumption of alcohol is not allowed within 48 hours prior to the screening visit and 48 hours prior to and during all subsequent visits to the clinic including the end-of-study visit of each part. In addition, consumption of alcohol is disallowed during residential stays.

5.5.9 Activity Restrictions

Exercise: Subjects and patients will abstain from strenuous exercise for 72 hours before each blood collection for clinical laboratory tests.

5.5.10 Additional Restrictions

Nicotine: Smoking or use of nicotine-containing products is not allowed from 6 months prior to screening until the end-of-study visit in Parts 1 and 2.

In Part 3, smoking is not permitted for 2 hours pre-dose until 4-hours post first dose on Day 1 and Day 22.

Blood donation: Subjects and patients must not donate blood or plasma during the study until 3 months after the final medical examination at the end-of-study visit of each part.

Sun Exposure (Part 3 only): The use of sun beds is not permitted from 4 weeks prior to screening until the end of study visit in Part 3. In addition, patients must avoid significant sun exposure from screening until after the end of study visit.

Sun Exposure (Part 1 and Part 2 only):

Investigators will advise subjects to minimize exposure to sunlight and UV light for the duration of the treatment period and for at least 48 hours after the last dose (each individual dose for the Food Effect cohort). High protection factor sun cream may be provided.

6. Investigational Medicinal Products

6.1 S011806 and Placebo

The investigational medicinal product (IMP) for this study is S011806. S011806 and matching placebo will be supplied by the Sponsor in two strengths: 25mg and 200mg of active substance, in 125mg and 1g tablet format, respectively. Tablets are packaged in high-density polyethylene (HDPE) bottles and closed with child-resistant caps.

The tablet formulation consists of S011806 active drug substance (in 25mg or 200mg strength) compressed with inactive ingredients (refer to Investigator's Brochure). Corresponding placebo tablets will be provided, consisting of the same excipients and compressed to the same size, shape and colour, hence will be identical in appearance to the active tablets.

For more information on the formulation, packaging and handling of S011806 and matching placebo, see the Pharmacy Manual and the S011806 Investigator's Brochure.

Planned doses for Part 1 are listed in Section 2.2.1.1.

6.2 Administration of Investigational Medicinal Product

Unblinded study staff will prepare IMP on the basis of doses designated by the master randomization list. Subjects, patients, and blinded site personnel must be blinded to whether the subject /patient receives active IMP or placebo. The unblinded study staff will not perform study activities other than the preparation of IMP.

The IMP will be administered orally as tablet(s) with approximately 240ml of water with the subject or patient in a seated position. If needed to help swallow the tablets, additional water will be provided in 50-mL increments. Water volume may be adjusted based on the number of tablets administered.

In Part 1, subjects will be fasted (water permitted) for at least 10 hours prior to and 4

hours post morning dose administration. Water is not permitted in the Food Effect cohort (both treatment periods) for 1 hour prior to - and 1-hour post-dose with the exception of the water required to swallow the IMP. Subjects in the Part 1 food effect cohort will be given a high-fat breakfast (see Table 3) 30 minutes prior to administration of the IMP in treatment period 2, a drink is permitted with breakfast. Subjects should eat this meal in 30 minutes or less. The IMP should be administered 30 minutes after start of the meal. Further details will be provided in the Study Reference Manual.

Table 2. Food Effect Cohort High Fat Meal Guidance [16]

Approx. Protein	150 calories
Approx. Carbohydrate	250 calories
Approx. Fat	500-600 calories
Approx. total calories	800-1000 calories

In Part 2, QD dose or morning dose of a BID or TID cohort will be dosed fasted, the same as in Part 1. In the event of twice or three times daily dosing, subjects will also be required to fast (water permitted) for 2 hours prior to and 1 hour post mid-day or evening dose. Fasting windows may be adjusted depending on emerging PK data. Modifications to fasting windows are permitted to allow volunteers to eat 3 times per day. Evening doses will be administered approximately 12 hours (±1 hour) after the morning dose. Mid-day or evening doses for the TID dosing schedule (if PK data suggest so) will be dosed approximately 8 hours apart from the earlier dose.

In the event of little or no food effect being observed in the Part 1 food effect cohort, dose can be given with or without food in all subsequent Part 2 cohorts.

In Part 3, instructions for intake of IMP with food or with fasting will be defined by PK data from Part 1 and 2 and will be given to patients prior to initiation of treatment.

6.2.1 In-Clinic Administration of IMP

Blinded study staff will administer the IMP in Parts 1 and 2. The exact time of dosing will be based on logistics and will be documented in source data. The time will be taken from the administration of the first tablet where dosing requires multiple tablets.

Subjects in Part 1 will receive a single oral dose of S011806 on one occasion under the supervision of study staff at the CRU. Subjects in the food-effect cohort will receive a second oral dose of S011806, after IMP washout (at least 5 half-lives of S011806 or 7 days, whichever is longer), under the supervision of study staff at the CRU.

For 7-day treatment cohorts, subjects in Part 2 are expected to receive a total of 7 (QD), 13 (BID) or 19 (TID) oral doses of S011806; 1 dose per day for 7 days, 2 doses per day for 6 days and one dose on Day 7 or 3 doses per day for 6 days and one dose on day 7 under the supervision of study staff at the CRU.

For 10-day treatment cohorts, subjects in Part 2 are expected to receive a total of 10 (QD), 19 (BID) or 28 (TID) oral doses of S011806; 1 dose per day for 10 days, 2 doses per day for 9 days and one dose on Day 10 or 3 doses per day for 9 days and one dose on Day 10 under the supervision of study staff at the CRU.

6.2.2 At-home Administration of IMP (Part 3 only)

Patients in Part 3 are expected to receive a total of 28 (QD), 56 (BID) or 84 (TID) oral doses of S011806; one, two or three doses per day for 28 days. In Part 3, patients will take their first dose (Day 1) and any subsequent doses due whilst resident (including Day 2 morning dose) under the supervision of study staff at the CRU. All other doses can be taken at home by the patient or at the CRU. Tablets for at-home administration are packaged in high-density polyethylene (HDPE) bottles and closed with child-resistant caps. Patients will be supplied with enough extra tablets to allow for flexibility in visit scheduling, as described in the SOA (Section 1.2.8). Patients will be asked to take the IMP at approximately the same time each day as it was administered on Day 1 (±2 hours). In the case of once-daily dosing, patients will be advised to take their dose in the morning. In the case of a missed dose, patients will be instructed not to take a “catch-up dose” but

to resume dosing with the next regularly scheduled dose. Subjects/patients should continue with the remaining doses as normal. Patients will be provided with a daily dosing diary to complete during periods of home dosing.

Patients will be advised to store the IMP safely, out of reach of children, at room temperature. On CRU visits days, patients will be instructed to bring all medications back to the unit and not to dose at home on the morning of study visits. Patients will receive their morning dose in the unit on study visit days after study procedures detailed in Section 1.2.8.

Detailed instructions for dose administration and storage will be provided in a Pharmacy Manual.

6.3 Preparation, Handling, Storage and Accountability

- The Investigator or designee must confirm appropriate temperature conditions have been maintained during transit for all IMPs received and any discrepancies are reported and resolved before use of the IMP.
- Only subjects/patients who meet eligibility criteria for the study may receive study IMP and only authorised site staff may supply or administer IMP. All IMP stored at site must be stored in a secure, environmentally controlled, and monitored (manual or automated) area in accordance with the labelled storage conditions with access limited to the Investigator and authorised site staff, as described in the Pharmacy Manual
- The Investigator, institution, or the head of the medical institution (where applicable) is responsible for IMP accountability, reconciliation, and record maintenance (i.e., receipt, reconciliation, and final disposition records).
- The pharmacy will take measures to ensure that the study staff, subjects and patients remain blinded.
- Accurate records of all IMPs received at, dispensed from, returned to, and disposed of by the study site should be recorded on the IMP accountability log.

- Further guidance and information for the final disposition of used and unused study IMP are provided in the Pharmacy Manual.

6.4 Minimisation of Bias

6.4.1 Randomisation and Subject/Patient Numbering

This is a 3-part, double-blinded, randomised, placebo-controlled trial. Subjects and patients will be randomised with a randomisation schedule for each dosing cohort and study part. The randomisation will be 6:2, active: placebo in Parts 1 and 2 and a ratio of 2:1, active: placebo in Part 3. Subjects and patients will be randomly allocated on Day 1 prior to first dose.

Each subject/patient will be assigned a unique screening identification number (SCR 001, 002, 003 onwards) chronologically assigned upon consent.

Subjects and patients who complete the study screening assessments and meet all the eligibility criteria will be assigned a unique randomisation number, allocated sequentially by a central site, prior to the first dose. This will be different from the screening number and subjects/patients will receive the corresponding product according to a randomisation scheme. The randomisation number will follow the following convention, PCRNN, where P equals study part (1, 2, 3), C equals study cohort beginning with 1, R equals a sequential replacement number beginning with 0, 1 for the first replacement etc., and NN equals a sequential number beginning with 01.

6.4.2 Blinding

The randomisation scheme will only be available to CRU Pharmacy staff who are not involved in any other aspect of the study, including drug administration. It will not be available to the subjects, patients, PI or study staff responsible for any study procedures with the exception of circumstances detailed in Section 6.4.3.

For each dose strengths, S011806 and a matching placebo will both be of the same weight, shape, size and colour to ensure blinding is maintained.

6.4.3 Procedures for Breaking the Blind Prior to Study Completion

A subject's/patient's treatment assignment will only be unblinded when knowledge of the treatment is essential for the further clinical management of the subject/patient on this study or may potentially impact the safety of subjects/patients currently enrolled or subjects/patients in subsequent enrolment. If required to inform dose regimen or internal decision making, blinded Sponsor delegates involved in dose escalation may be unblinded on completion of any Part 1 or 2 cohort. Unblinding of anyone directly involved in the conduct of the study will not occur for the purpose of the interim analysis detailed in Section 9.4. Unblinding at the study site for any other reason will be considered a protocol deviation. In case of an emergency, the Investigator has the sole responsibility for determining if unblinding of a subject's/patient's treatment assignment is warranted. Subject/patient safety must always be the first consideration in making such a determination.

One set of sealed envelopes containing the randomisation code will be supplied to the Investigator or designee at the start of the study. Additional sets of sealed envelopes will be provided to Diamond Pharma Services and Alderley Analytical if required.

If breaking the blind is required because of a medical emergency, the treatment identity would be revealed by the Investigator or designee for that subject only. In the event that the emergency is one in which it appears that the other subjects may be at imminent risk, the blind may be broken for all subjects dosed at that dose level. The unblinding will be properly documented in the study file. The responsibility to break the blind resides solely with the Investigator (or designee), however, it is requested that the Investigator or designee will make every effort to contact the Medical Monitor or designee to notify him/her of the medical emergency and the breaking of the blind as soon as it is practicable, granting that these efforts should not stall or delay the unblinding of trial

subject/patient treatment in emergency situations. The Investigator or designee will ensure that the minimum number of personnel involved with the study will be aware of the treatment assignment after unblinding.

In all cases where the code is broken, the Investigator or designee should record the date and reason for code breaking.

At the end of the study, envelopes will be retained according to site procedures.

6.5 Investigational Product Compliance

In Parts 1 and 2, subjects will complete all study procedures, including dose administration, at the CRU under the supervision of study staff. To ensure the tablet has been swallowed, mouth and dosing vessel checks will be conducted following dose administration in all cases.

In Part 3, dose administration will be completed by the patient at home when not attending the CRU for safety, PK, or PD procedures (see Section 1.2.8). Site staff will complete study drug accountability and assess compliance at each weekly visit. If a dose is missed or the complete dose is not taken, this will be recorded as a deviation and the Sponsor will be informed. Any significant non-compliances will be discussed with the Sponsor.

6.6 Criteria for Dose Escalation (Parts 1 and 2)

A decision to proceed to the next higher dose administration in Parts 1 and 2 will be made by the SAC. The SAC will include the Sponsor’s physician (or delegate) and the Principal Investigator (or delegate) as a minimum. Other representatives may be considered on a case-by-case basis, if additional expertise is required. Dose levels for Part 1, dose duration for Part 2 and dose levels and frequency of dosing for Part 2 and 3 will be confirmed during the SAC review and may be modified when appropriate based on available safety and PK data. Dose modifications in Part 1 shall not exceed 3-fold

escalation and a maximum dose of 800mg. Dose level and frequency modifications in Part 2 and 3 will not exceed exposures tested in Part 1 and 2, respectively.
The objective of this study is not to determine a maximum tolerated dose; however, if an intolerable dose has been identified, the exposure at that dose will not be repeated or exceeded in subsequent cohorts. A dose level below this dose may be tested.

All pertinent safety and tolerability data (e.g., physical examinations, vital signs assessments, safety 12–lead ECGs, clinical laboratory tests, C-SSRS and AEs) and all available PK data at least 24 hours post dose (Part 1) and at least 48 hours post final dose (Part 2) will be reviewed to inform dose escalation decisions. In addition, available PK data from previous cohorts will be included in the review, for context.

At least 6 of the planned subjects for each dose level must complete dosing and data review for the next dose level to proceed in both Part 1 and 2.

Part 2 may commence prior to the completion of Part 1. Part 2 may commence after a Part 1 cohort at least one dose level higher than Part 2 starting dose has been reviewed by the SAC. Subsequent Part 2 cohorts may only commence after a Part 1 cohort at least one dose level higher or all Part 1 cohorts, **and** prior Part 2 cohorts have been reviewed by the SAC.

Individual safety reports will be generated for each subject based on review of source documents by two independent physicians at the study site, according to site SOP to comply with the MHRA-required QC step for clinical data related to dose escalation decisions. Following dose escalation review, and if none of the stopping rules (Section 6.7) are met, a decision will be made to continue the dose escalation as planned, to continue with the study by repeating the current dose, or to adjust to an intermediate dose lower than the planned next dose.

The maximum-targeted increase in exposure from cohort to cohort will be up to 3-fold. In general, with each subsequent SAD escalation, progressively smaller fold increases in

exposure will be targeted.

Single doses will not exceed 800 mg of S011806 based on the proposed maximum efficacious dose level and safety margins calculated following non-clinical studies (Section 2.2). Dose escalation to a level which yields a systemic exposure higher than pre-defined maximum exposure limits will not occur without a protocol amendment.

The study is planned to be conducted entirely by a single site, Medicines Evaluation Unit (MEU), Manchester, UK, however additional sites may be added if required. Additional sites will be submitted as a substantial amendment.

Additional details describing the logistical aspects of dose escalation will be included in a separate Dose Escalation Charter or Study Reference Manual.

6.7 Stopping Criteria

If a stopping criterion is determined to have been met at any point in the study, including during any SAC review, the party aware of the stopping criterion being met will notify the Investigator at the clinical site and the Sponsor via e-mail or phone call as soon as possible, but no more than 24 hours from the time of becoming aware of the criterion being met.

In the event of study termination, a written statement fully documenting the reasons for termination will be provided to the ethics committee (EC) and Medicines and Healthcare products Regulatory Agency (MHRA).

The Sponsor and the Investigator reserve the right to discontinue this study or the participation of any subject or patient at any time for any reason (see Section 7). Certain circumstances may require the premature termination of dosing in a cohort and subsequent cohorts at higher doses and/or the study, if the Investigator or the Sponsor feel that the type, number, relatedness, and/or severity of AEs justify discontinuation of

the dosing/study.

Study and cohort stopping rules are listed in Table 3.

Table 3. Study and Cohort Stopping Rules

**Adverse Event Stopping Criteria**	
AE Severity/Seriousness	Effect on Cohort Dose and Study
Severe<br>Non-Serious	If any AE possibly or probably related to S011806 occurs in ≥2 subjects/patients regardless of cohort:<br>- Suspend dosing in the cohort<br>- A lower dose level or an intermediate (but lower) dose level may be administered in the next cohort<br>- Approval by SAC, EC and regulatory authority, required to resume dosing at same or higher dose level
Serious<br>Non-life threatening/non-fatal	If any AE probably or possibly related to S011806 occurs in ≥1 subject/patient regardless of cohort:<br>- Suspend dosing in the cohort<br>- A lower dose level or an intermediate (but lower) dose level may be administered in the next cohort<br>- Approval by SAC, EC and regulatory authority, required to resume dosing at same or higher dose level
Serious<br>Life threatening/ fatal	If probably or possibly related to S011806:<br>- Stop all dosing on the study<br>- Approval by SAC, EC and regulatory authority, required to resume dosing at any dose level
**PK/Exposure Stopping Criteria**	
Systemic exposure	Effect on Cohort Dose and Study
Predicted to exceed the mean systemic exposure associated with the dog NOAEL Cmax and AUC exposures (20,050 ng/mL and 74,700 ng*h/mL, respectively)	If ≥ 1 subject/patient in the same cohort is predicted to exceed the mean systemic exposure indicated:<br>- Suspend dosing in the cohort<br>- A lower dose level or an intermediate (but lower) dose level may be administered in the next cohort<br>- Approval by SAC, EC and regulatory authority required to resume dosing at same or higher dose level

AE relatedness will be determined by the Investigator (see Section 10.3). If the study or cohort is halted, a temporary halt will be submitted to the MHRA and EC in the form of a substantial amendment. The study or cohort will not be resumed until a further substantial amendment to resume the study is submitted and approved by MHRA and EC.

7. Discontinuation of Investigational Medicinal Product and Subject/Patient Discontinuation or Withdrawal

7.1 Discontinuation of Investigational Medicinal Product

Subjects/patients will be withdrawn from IMP for the following reasons:

- Subjects/patients will be withdrawn from IMP if they exhibit a SAE and/or any AEs that in the opinion of the PI may jeopardise their safety.
- QTcF interval of >500 ms or increase in QTcF interval of >60 ms from baseline as confirmed by mean triplicate measurement.

  For the purpose of QTcF withdrawal criteria, baseline will be considered as the mean pre-dose Day 1 measurement for all parts. For treatment period 2 in the food effect cohort, baseline will be considered as the mean pre-dose of treatment period 2, Day 1 measurement. The withdrawal criterion will be assessed against the mean QTcF of the repeat triplicate ECGs. ECGs will be performed within a time window of 5 minutes, with minimum of 1 minute between subsequent ECGs.
- Increase of total bilirubin greater than or equal to 3x the upper level of normal.
- Increase of AST or ALT ≥ 3 x Upper Limit of Normal (ULN) <u>and</u> total bilirubin ≥ 2xULN (>35% direct bilirubin).
- Increase of AST or ALT ≥ 3xULN if associated with symptoms (new or worsening) believed to be related to hepatitis (such as fatigue, nausea, vomiting, right upper quadrant pain or tenderness or jaundice) or believed to be related to hypersensitivity (such as fever, rash or eosinophilia). Subjects/patients with AST or ALT ≥ 3xULN and < 5xULN and bilirubin < 2xULN, who do not exhibit hepatitis symptoms or rash, will be allowed to continue study treatment as long as they are monitored at every visit.
- Isolated ALT/AST ≥ 5xULN confirmed by repeat.
- Pregnancy of female subject/patient or female partner of male subject/patient.
- Upon the subject/patient's request (withdrawal of consent)
- Significant deviation from the protocol is considered, in the opinion of the Investigator or Sponsor, to jeopardise the subject/patient's safety.
- Requirement for prohibited medication

- At the discretion of the Investigator
- Termination of the study for any reason by the Sponsor

For a subject/patient who withdraws or is withdrawn, every effort will be made to ensure the subject/patient completes early termination procedures (if applicable) as soon as possible after discontinuation and as detailed in the study schedule of activities in Section 1.2. The scheduled safety follow-up visit will also be performed, if possible, if the early discontinuation visit has been performed prior to the scheduled follow up period.

7.2 Subject/Patient Replacement

Subjects or patients who withdraw or are withdrawn from the study may be replaced at the discretion of the Sponsor upon discussion with the Principal Investigator. At least 6 subjects per cohort for Part 1 and 2 and 10 patients per cohort for Part 3 should be available for dose escalation review and final analysis (i.e. received a dose of IMP and has sufficient data for evaluation of the safety and PK objectives). If fewer, subjects/patients may be replaced to achieve the intended number of subjects/patients.

7.3 Subject/Patient Discontinuation or Withdrawal from the Study

- A subject or patient may withdraw from the study at any time at his/her own request or may be withdrawn at any time at the discretion of the Investigator for safety, behavioural, compliance, or administrative reasons. This is expected to be uncommon.
- At the time of discontinuing from the study, if possible, early termination procedures (if applicable) as detailed in the study schedule of activities (Section 1.2. should be performed as soon as possible after discontinuation. A safety follow-up visit will also be performed as per schedule of activities (Section 1.2) at the specified time after the last dose of IMP administered.
- The subject/patient will be permanently discontinued both from the study medication and from the study at that time.

- If the subject/patient withdraws consent for disclosure of future information, the Sponsor may retain and continue to use any data collected before such a withdrawal of consent.
- If a subject/patient withdraws from the study, he/she may request destruction of any samples taken and not tested, and the Investigator must document this in the site study records.

7.4 Lost to Follow Up

A subject or patient will be considered lost to follow-up if he or she repeatedly fails to return for scheduled visits and is unable to be contacted by the study site.

The following actions must be taken if a subject/patient fails to return to the clinic for a required study visit:

- The site must attempt to contact the subject/patient and reschedule the missed visit as soon as possible and counsel the subject/patient on the importance of maintaining the assigned visit schedule and ascertain whether or not the subject/patient wishes to and/or should continue in the study.
- Before a subject or patient is deemed lost to follow up, the Investigator or designee must make every effort to regain contact with the subject/patient (where possible, three telephone calls and, if necessary, a certified letter to the subject/patient's last known mailing address or local equivalent methods). These contact attempts should be documented in the subject/patient's source data records.
- Should the subject or patient continue to be unreachable, he/she will be considered to have withdrawn from the study.

8. Study Assessments and Procedures

Study procedures are detailed in the schedule of activities Section 1.2 and will be performed in accordance with site SOP's unless otherwise stated in this protocol.

8.1 COVID-19 screening

All subjects and patients will be asked a series of COVID-19 screening questions on telephone screen and upon arrival at every visit to the clinical site. In accordance with local site procedures

8.2 Screening

Informed consent will be obtained during a separate visit prior to, or at screening before any study procedures. Subjects and patients will be screened for eligibility to participate in the study within the 28 days preceding first dose (Day 1). All subjects and patients will be required to attend the CRU to undergo screening procedures in accordance with the schedule of assessment (Section 1.2).

Screening procedures may be performed over more than one day. Repeats of study procedures are permitted on the day and/or another day during screening at the discretion of the Investigator.

8.3 Admission and Pre-dose Procedures

In Parts 1 and 2, subjects will be admitted to the CRU on the evening of Day -1. In Part 3, patients will attend the CRU on the morning of Day 1. The ongoing eligibility of subjects and patients will be reassessed on admission and pre-dose as applicable (See Section 1.2). Baseline procedures will be performed pre-dose on Day 1 with the exception of dermatology assessments which will be performed in the baseline window (see Section 1.2.8).

8.4 Timing of Procedures

Recording of AEs and concomitant medications will be made prior to all other study procedures. All safety assessments will be timed and performed relative to the start of dosing (for the food effect cohort, start of dosing will be relative to each treatment period). If a dose comprises multiple tablets, start of dosing will be relative to the first tablet.

Permitted windows for all relevant study procedures will be provided in the Study Reference Manual. Where the protocol requires more than one procedure to be completed at the same time point, all efforts will be made to obtain the PK samples at the exact nominal time relative to dosing. Any other procedures scheduled at the same time point will be performed either before or after the PK, as appropriate. ECGs should be taken prior to vital signs when both measurements are scheduled at the same time point.

8.5 Discharge from the Clinical Research Unit

Providing there are no safety concerns, subjects and patients will be discharged from the CRU as detailed below:

- In Part 1, subjects will be allowed to leave the CRU following completion of the 48-hour post dose procedures on Day 3 as detailed in the schedule of assessments (Section 1.2.1, 1.2.2 and 1.2.3) following IMP dose. This applies to both treatment periods individually for the food effect cohort.
- In Part 2, subjects will be allowed to leave the CRU following completion of the 48-hour post dose procedures on Day 9 (7 day dosing) or Day 12 (10 day dosing) as detailed in the schedule of assessments (Section 1.2.4, 1.2.5, 1.2.6 and 1.2.7) following final IMP dose.
- In Part 3, patients will be resident overnight from Day 1 to Day 2 then for subsequent visits will attend as outpatients attending the CRU as detailed in Section 1.2.8. Following the first dose of IMP (Day 1), patients will remain resident in the CRU overnight and until the completion of Day 2 pre-dose procedures as detailed in Section 1.2.9 and will receive the Day 2 morning dose prior to discharge as detailed

in Section 1.2.9.

8.6 Return Visits

In Part 1, subjects will be required to attend a follow up visit for safety assessments 7 (± 2) days after IMP administration.

In Part 2, subjects will be required to attend a follow up visit for safety assessments 7 (± 2) days after final IMP administration.

In Part 3, patients will be required to attend the CRU for safety, PK, PD and clinical evaluations and IMP administration on Days 8, 15, 22, and 29, as detailed in Section 1.2.8. In addition, patients will be required to attend a follow up visit for safety assessments 15(± 2) days after final IMP administration.

8.7 Early Discontinuation Procedures and Visits

If a subject or patient withdraws or is withdrawn prior to completion, safety procedures as detailed in Section 1.2 will be performed prior to discharge from the CRU.

At the discretion of the Investigator, procedures scheduled for early termination may not be repeated if already conducted on the same day. Subjects/patients will be asked to return to the CRU for an early termination visit as soon as possible after discontinuation if they are not resident at the time of withdrawal. In the event a subject/patient is not able to attend an early termination visit, the scheduled follow up visit is still required. The scheduled safety follow-up visit will also be performed, if possible, if the early termination visit has been performed prior to the scheduled follow up period.

8.8 Blood Volume

The volume of blood donated during the study will not exceed the maximum recommended by the Joint United Kingdom blood Transfusion and Tissue

Transplantation Services Professional Advisory Committee (JPAC) [17].

8.9 Medical Supervision

A physician will be responsible for the clinical aspects of the study and will be available at all times during the study. In accordance with the current Association of the British Pharmaceutical Industry guidelines [18], each subject will receive a card stating the telephone number of the Investigator.

8.10 Safety Assessments

8.10.1 Physical Examinations

Subjects and patients will undergo a physical examination at screening as detailed in Section 1.2. A full physical examination will involve assessment of the following: general appearance; ears, nose and throat; head, neck and thyroid; cardiovascular; respiratory; lymph nodes; abdomen; skin; musculoskeletal and nervous system. An abbreviated physical examination will involve assessment of the following: cardiovascular; respiratory; abdomen and, any other system if deemed necessary. A symptom-directed physical examination will involve any body system deemed necessary at the Investigator's discretion due to on-going AEs at the time of assessment. Any clinically significant changes will be recorded as AEs.

8.10.2 Vital Signs

Blood pressure and pulse rate will be measured after the subject/patient has been in a supine position for a minimum of 10 minutes according to the schedule of activities presented in Section 1.2. Repeat tests are permitted on the day and/or on another day (as appropriate) at the discretion of the Investigator. Blood pressure and pulse rate will be measured by automated equipment. Tympanic temperature will be measured according to the schedule of activities presented in Section 1.2.

8.10.3 Body Weight and Height

The subject/patient's body weight and height will be recorded as detailed in Section 1.2. Measurements will be taken in normal indoor clothing with shoes removed.

8.10.4 Electrocardiograms

At screening and baseline (pre-dose Day 1), 12-lead ECGs will be measured in triplicate after the subject/patient has been in the supine position for a minimum of 10 minutes according to the schedule of activities presented in Section 1.2. Repeats are permitted on the day and/or on another day (as appropriate) at the discretion of the Investigator. For assessment of time windows, the time of the first ECG of the triplicate will be used. ECGs should be taken prior to vital signs when both measurements are scheduled at the same time point.

Triplicate ECGs will be performed within a time window of 5 minutes, with minimum of 1 minute between subsequent ECGs. At all other scheduled time points, safety ECGs will be conducted as a single 12-lead ECG following a minimum of 10 minutes supine rest according to the schedule of activities presented in Section 1.2. Repeats are permitted on the day and/or on another day (as appropriate) at the discretion of the Investigator. See Section 8.3 for definition of baseline for each study part. Further details are provided in the Study Reference Manual.

8.10.5 Cardiac Holter Monitoring (Parts 1 and 2 only)

In Parts 1 and 2, Holter monitors will be used to collect continuous 12-lead ECG data for at least 1 hour pre-dose on Day 1 and for at least 24 hours post-dose. In Part 2, Holter monitors will also be used to collect continuous 12-lead ECG data for at least 1 hour pre-dose on Day 7 or 10 (depending on duration of dosing) and for at least 24 hours post-dose (Day 8 or 11). Further details are provided in the Study Reference Manual.

8.10.6 Additional Safety Procedures

Additional non-invasive procedures that are already specified in the protocol may be performed, if it is believed that an important effect of the IMP is occurring or may occur at a time when no measurements are scheduled, or if extra procedures are needed in the interests of safety. Additional blood samples for safety assessments may be taken if required by the Investigator at any point.

8.10.7 Clinical Safety Laboratory Assessments

Available blood and urine sample results will be reviewed by a physician before the subject/patient is dosed or receives their next dose, or is released from the study, as is appropriate.

A list of the laboratory parameters measured is presented in Section 10.2.

8.10.7.1 Haematology, Clinical Chemistry, Coagulation and Virology

Laboratory tests will be performed by the site's local laboratory according to the schedule of activities presented in Section 1.2. Repeats are permitted on the day and/or on another day at the discretion of the Investigator.

Site standard collection and processing procedures for blood samples will be adhered to throughout the study. Scheduled blood samples for clinical chemistry will be taken following at least an 8 hour fast (water is permitted).

8.10.7.2 SARS-CoV-2 (Nasal/Throat swab)

SARS-CoV-2 testing will be performed on site or by the site's local laboratory according to the schedule of activities presented in Section 1.2. Repeats and unscheduled tests, including PCR and lateral flow (rapid testing), are permitted on the day and/or on another day at the discretion of the Investigator.

Site standard collection and processing procedures for samples will be adhered to throughout the study.

8.10.7.3 Urinalysis

Urinalysis will be performed by the site's local laboratory according to site-specific SOPs according to the schedule of activities presented in Section 1.2. Repeats are permitted on the day and/or on another day at the discretion of the Investigator. Site standard collection and processing procedures for urine samples will be adhered to throughout the study.

8.10.7.4 Pregnancy Test

Pregnancy tests (serum test at screening, follow up and early termination visit [performed by site's local laboratory], on-site urine dipstick testing at all other time points) will be performed for all female subjects and patients of childbearing potential, as detailed in Section 1.2. Repeats are permitted on the day and/or on another day at the discretion of the Investigator. Site standard collection and processing procedures for samples will be adhered to throughout the study.

8.10.7.5 Follicle-Stimulating Hormone (FSH) Test

Serum FSH tests may be performed by the site's local laboratory for post-menopausal female subjects and patients at the discretion of the Investigator to confirm post-menopausal status at screening. Site standard collection and processing procedures for blood samples will be adhered to throughout the study.

8.10.7.6 Drug Screen and Urine Cotinine

A urine drug screen, including urine cotinine in Parts 1 and 2, will be performed on-site using a dipstick method according to the schedule of activities presented in Section 1.2. Repeats are permitted on the day and/or on another day at the discretion of the Investigator. Site standard collection and processing procedures for urine samples will be

adhered to throughout the study. Subjects/patients will be screened for the drugs of abuse listed in Section 10.2.

8.10.7.7 Alcohol Breath Test

An alcohol breath test will be performed according to the schedule of activities presented in Section 1.2. Repeats are permitted on the day and/or on another day at the discretion of the Investigator. A confirmed positive result will exclude the subject/patient from screening or dosing during admission.

8.10.7.8 Abnormal Laboratory Findings

In cases where laboratory findings are outside the normal range and the Investigator believes that the results may be of clinical significance, repeat sampling may be requested as clinically indicated. If the abnormal finding is clinically significant, appropriate actions will be taken e.g. the subject/patient will not be entered into the study or the subject/patient may be withdrawn from the study. The subject/patient will be referred to their GP or other appropriate provider for further care. The same will apply if the results of the HBsAg, HCV Ab or HIV test are positive and in addition the Investigator will ensure that adequate counselling is available, if requested.

Abnormal results at follow-up assessments will also require repeat testing if the Investigator believes the results may be of clinical significance. Any clinically significant abnormality, including changes from baseline (pre-dose Day 1 or last non-missing value pre-dose), must be reported as an AE.

Additional blood, urine and/or throat swab samples may be taken for safety tests. Furthermore, additional assays outside those specified in the protocol may be performed for safety reasons as requested by the Investigator.

8.10.7.9 Columbia Suicide Severity Rating Scale (C-SSRS)

The Columbia Suicide Severity Rating Scale (C-SSRS) is utilized to prospectively assess and directly classifies suicidal ideation and behavior into 11 categories. The C-SSRS assesses lifetime and current suicidal thoughts and behaviours across these categories based on an increasing severity of a 1- to 5-rating scale. At the screening visit questions will be in relation to lifetime experiences and at all evaluations the questions will be in relation to the last assessment. The C-SSRS is to be completed by the Investigator or by a qualified designee at every visit according to the schedule of activities presented in Section 1.2. C-SSRS 'Baseline/Screening Questionnaire' to be used at screening and C-SSRS 'Since Last Visit' Questionnaires' are to be used for all other timepoints and visits.

8.11 Adverse Events and Serious Adverse Events

The definition of an AE or SAE can be found in Section 10.3. AEs will be reported by the subject /patient (or, when appropriate, by a caregiver, surrogate, or the subject /patient's legally authorized representative). The Investigator and any qualified designees are responsible for detecting, documenting, and recording events that meet the definition of an AE or SAE and remain responsible for following up AEs that are serious, considered related to the study IMP or study procedures, or that caused the subject /patient to discontinue IMP (see Section 10.3.3)

8.11.1 Time Period and Frequency for Collecting AE and SAE Information

All AEs and SAEs will be collected from the signing of the informed consent form (ICF) until the last follow-up visit (Section 10.3.3)

AEs and SAEs will be reported in the CRF from signing of informed consent.

All SAEs will be recorded and reported to Diamond Pharma Services, the Sponsor and the Medical Monitor immediately and under no circumstance should this exceed 24 hours, as indicated in Section 10.3.4. The Investigator will submit any updated SAE data to the Sponsor within 24 hours of it being available.

Investigators are not obligated to actively seek AEs or SAEs after conclusion of the study participation. However, if the Investigator learns of any SAE, including a death, at any time after a subject/patient has been discharged from the study, and he/she considers the event to be reasonably related (probable related or possible related) to the study IMP or study participation, the Investigator must promptly notify the Sponsor.

8.11.2 Method of Detecting AEs and SAEs

The method of recording, evaluating, and assessing causality of AEs and SAEs and the procedures for completing and transmitting SAE reports are provided in Section 10.3.3. Care will be taken not to introduce bias when detecting AEs and/or SAEs. Open-ended and non-leading verbal questioning of the subject/patient is the preferred method to inquire about AE occurrences.

8.11.3 Follow-up of AEs and SAEs

After the initial AE/SAE report, the Investigator is required to proactively follow each subject or patient at subsequent visits/contacts. All SAEs and Adverse Events of Special Interest (AESIs, see Section 8.11.6) will be followed until resolution, stabilisation, the event is otherwise explained, or the subject/patient is lost to follow-up (as defined in Section 7.4). Further information on follow-up procedures is provided in Section 10.3.3.

8.11.4 Regulatory Reporting Requirements for SAEs

- Prompt notification of a SAE by the Investigator to the Sponsor (via Diamond

Pharma Services) is essential so that legal obligations and ethical responsibilities towards the safety of subjects/patients and the safety of the IMP under clinical investigation are met.

- The Sponsor has a legal responsibility to notify both the local regulatory authority and other regulatory agencies about the safety of the IMP under clinical Investigation. The Sponsor will comply with country-specific regulatory requirements relating to safety reporting to the regulatory authority, Independent Ethics Committees (IEC), and Investigators.
- Investigator safety reports will be prepared for suspected unexpected serious adverse reactions (SUSAR) according to local regulatory requirements and Sponsor policy and forwarded to Investigators as necessary.
- An Investigator who receives an Investigator safety report describing a SAE or other specific safety information (e.g. summary or listing of SUSARs) from the Sponsor will review and then file it along with the Investigator's Brochure and will notify the IEC, if appropriate, according to local requirements.

8.11.5 Pregnancy

- Details of all pregnancies in female subjects/patients and female partners of male subjects/patients will be collected after first dose of IMP and until 90 days after the last dose as outlined in Section 10.4.
- If a pregnancy in a female subject/patient or the partner of a male subject/patient is reported, the Investigator should inform the Sponsor within 24 hours of learning of the pregnancy and should follow the procedures outlined in Section 10.4.4.
- Abnormal pregnancy outcomes (e.g., spontaneous abortion, fetal death, stillbirth, congenital anomalies, ectopic pregnancy) are considered SAEs.

8.11.6 Adverse Events of Special Interest

The following Adverse Events, which have been reported with antibody inhibition of IL-17 signaling, will be documented as AESIs:

- New onset of inflammatory bowel disease including Ulcerative Colitis or Crohn's Disease
- Suicidal ideation

AESIs will be reported to PPD within 24 hours after awareness at site.

8.12 Treatment of Overdose

There is no antidote for S011806. In the advent of an adverse reaction due to an overdose (i.e., taking more than the prescribed number of tablets), supportive and symptomatic treatment is indicated relevant to the clinical scenario. In serious cases subjects and patients should be hospitalised for observation and/or further intervention as necessary.

8.13 Pharmacokinetic Assessments

8.13.1 Pharmacokinetic Blood Sampling

Venous blood samples will be withdrawn via an indwelling cannula or by venepuncture according to the schedule of activities presented in Section 1.2. Samples will be collected into appropriate tubes as specified by the bioanalytical laboratory. Site standard collection and processing procedures for blood samples will be adhered to throughout the study.

Plasma will be shipped to Alderley Analytical for the analysis of S011806.

Samples will be analysed according to a validated method. Analytical methods are validated according to internationally accepted standards. The quality and integrity of the analytical work generated in this study will be evaluated in accordance with the study plan, validated method and SOPs of PPD

8.13.2 Pharmacokinetic Urine Sampling (Selected cohorts in Part 2 only)

Urine samples for pharmacokinetics will be collected at the specified time points outlined in the schedule of activities (Section 1.2). In Part 2, urine PK will be collected in selected cohorts. This will be confirmed during interim data review with collection planned for cohorts 2 and 4.

Site standard collection and processing procedures for urine samples will be adhered to throughout the study. Details can be found in the Study Reference or Laboratory Manual. Urine will be shipped to PPD for the analysis of S011806 as well as preliminary metabolite assessment.

8.14 Pharmacodynamic Assessments - Part 3 only

8.14.1 Pharmacodynamic Blood Sampling

Venous blood samples will be withdrawn via an indwelling cannula or by venepuncture according to the schedule of activities presented in Section 1.2. Samples will be collected into appropriate tubes as specified by the bioanalytical laboratory. Site standard collection and processing procedures for blood samples will be adhered to throughout the study. Details can be found in the Study Reference/Laboratory Manual.

Serum will be analysed for exploratory PD biomarkers including but not limited to serum levels of inflammatory cytokines (e.g. IL-17A) and beta defensin-2 (BD-2) by ELISA.

Samples will be analysed according to the Analytical Laboratory's methods. The quality and integrity of the analytical work generated in this study will be evaluated in accordance with the study plan and SOPs of analytical Laboratory.

8.14.2 Skin Plaque Biopsy

Skin plaque biopsies (punch biopsies) will be collected according to the schedule of

activities presented in Section 1.2. Analysis will include histological analysis including but not limited to hematoxylin and eosin staining and RNA analysis including but not limited to gene expression of beta defensin-2 and IL-17 A, F. Details of skin plaque biopsy collection will be provided in the Study Reference Manual.

8.15 Clinical Activity - Part 3 only

8.15.1 Percent of Body Surface Area Involvement (BSA)

The percent of BSA involvement will be estimated for each patient according to the schedule of activities presented in Section 1.2. Details of the BSA assessment will be provided in the Study Reference Manual.

8.15.2 Psoriasis Area and Severity Index Score (PASI)

The PASI score will be assessed according to the schedule of activities presented in Section 1.2. Details of the PASI assessment will be provided in the Study Reference Manual.

8.15.3 Physician Global Assessment (PGA)

The PGA score will be assessed according to the schedule of activities presented in Section 1.2. Details of the PGA will be provided in the Study Reference Manual.

8.15.4 Lesion Severity Score (LSS)

The LSS will be assessed according to the schedule of activities presented in Section 1.2. Details of the LSS will be provided in the Study Reference Manual.

8.15.5 Photography of Lesion Site

Digital photographs of lesion sites will be taken according to the schedule of activities presented in Section 1.2.The same locations photographed at baseline should be

followed throughout the study for each patient. Details for digital photography will be provided in the Study Reference Manual.

9. Statistical Considerations

9.1 Sample Size Determination

The sample size for this study was selected on the basis of clinical considerations for Phase 1 studies, and due to the exploratory nature of this study, no formal power or hypothesis testing were considered to determine sample size. A sample size of 8 subjects (6 active and 2 placebo) per cohort in Parts 1 and 2 and up to 21 patients (up to 14 active and up to 7 placebo) per cohort in Part 3 has been determined adequate to meet the study objectives.

9.2 Population for Analyses

Full Analysis Set: All subjects or patients randomised and receiving at least one dose of IMP, analysed according to the treatment/dose allocated.

Safety Analysis Set will consist of all subjects/patients who receive at least one dose of IMP. Subjects/patients will be analysed according to the initial treatment and dose actually taken.

Pharmacokinetic (PK) Concentration Set: All randomised subjects or patients who received at least one dose of active drug who have at least one post-dose plasma PK concentration recorded, without significant deviation or AE that may affect PK concentrations. The PK concentration analysis set will be used for presenting PK concentration data. Subjects/patients will be analysed according to the initial treatment and dose actually taken.

PK Parameter Analysis Set: All randomised subjects or patients who received at least one dose of active drug, without significant deviation or AE that may affect PK parameters and have sufficient data for at least one PK parameter to be determined. The PK

parameter analysis set will be used for presenting PK parameter data. Subjects/patients will be analysed according to the initial treatment and dose actually taken.

PD Analysis Set: All subjects or patients who received at least one dose of the study drug (active or placebo) and had at least one pre-dose and one post-dose evaluable measurement of any of the evaluable PD assessments. Subjects/patients will be analysed according to the initial treatment and dose actually taken.

9.3 General Analyses

Full details of the statistical analyses will be provided in a separate statistical analysis plan (SAP). Statistical analysis will be descriptive and exploratory. Continuous data will be summarised using descriptive statistics (n, mean, SD, median, minimum, and maximum) and categorical data will be summarised using the number and percentage of subjects/patients in each category. Where confidence limits are appropriate, the confidence level will be 95% (two-sided), unless otherwise stated. Data will be presented separately for Parts 1, 2, and 3 and as appropriate by treatment group and dose. Data from placebo treated subjects/patients will be pooled across dosing cohorts within each study Part. Unless otherwise stated, for the purposes of summaries and analyses, baseline will be defined as the last non-missing assessment value prior to first dose. For the food effect cohort, baseline for each treatment period will be defined as the last non-missing assessment value prior to first dose for the respective treatment period. Further details regarding selection of baseline for specific endpoints will be given in the SAP.

9.3.1 Demographic Data and Baseline Characteristics

Demographics and baseline characteristics (including age, sex, race, ethnicity, height, weight, and BMI), medical history, and prior/concomitant medication will be summarised as appropriate. Concomitant medications will be coded using the most current WHO drug dictionary in place at the time of study start. Baseline characteristics refer to those collected at screening.

9.3.2 Safety Analysis

Safety data will be analysed on the safety analysis set. Adverse events (AEs) will be coded using the latest MedDRA dictionary version in place at the time of study start.

Incidence of treatment emergent adverse events (TEAEs) and incidence of TEAEs by system organ class (SOC) and preferred term (PT) will be presented as appropriate. Serious TEAEs and TEAEs leading to withdrawal will be similarly summarised. TEAEs will also be summarised by severity and relationship to IMP. Full detail will be provided in the SAP.

Clinical laboratory, vital signs, cardiac Holter monitoring and ECG parameters will be summarised as appropriate using summary statistics, with full detail provided in the SAP. Physical examination data will be listed.

9.3.3 PK Analysis

PK concentrations and PK parameters will be summarised for the PK concentration and PK parameter analysis sets respectively. Dose proportionality will also be assessed. Full detail will be provided in the SAP, including handling of missing or BLQ concentrations for concentration summaries and parameter derivation.

9.3.3.1 Plasma PK

PK parameters for plasma S011806 will be calculated using non-compartmental analysis as appropriate, including but not limited to:

For Part 1:

AUC_{last}:	The area under the plasma concentration time curve, from time 0 to the last measurable non-zero concentration.
AUC_{0-24}:	The area under the concentration time curve, from time 0 to the 24 hour time point. If the 24 hour plasma concentration is missing, below limit of quantification (BLQ), or not reportable, then this parameter

	cannot be calculated.
AUC_{0-inf}:	The area under the plasma concentration time curve from time 0 extrapolated to infinity. AUC_{0-inf} is calculated as the sum of AUC_{last} plus the ratio of the last measurable plasma concentration to the elimination rate constant (C_{last}/λ_z).
AUC%extrap:	Percent of AUC0-inf extrapolated, derived as (1 - AUC_{last}/AUC_{0-inf})*100.
CL/F:	Apparent total plasma clearance after extravascular administration, calculated as Dose/AUC_{0-inf}.
C_{max}:	Maximum observed concentration.
T_{max}:	Time to reach C_{max}. If the maximum value occurs at more than one time point, T_{max} is defined as the first time point with this value.
λ_z	Terminal rate constant.
$T_{1/2}$:	Apparent first order terminal elimination half-life will be calculated as 0.693/ λ_z.
Vz/F:	Apparent volume of distribution during the terminal elimination phase after extravascular administration, calculated as Dose/(AUC_{0-inf} x λ_z).

For the Food effect cohort, in addition to the above PK parameters:

Relative bioavailability following a high-fat meal versus fasted based on AUC_{0-inf} and C_{max}.

For Part 2, in addition to above PK parameters:

AUC_τ	AUC within a dose interval after the first and the last dose
$C_{trough,ss}$	Trough concentration at steady state
ARs	Accumulation ratios for C_{max}, and AUC_τ of dosing
C_{avg}	Average concentration at the steady state

For Parts 1 and 2:

As an exploratory assessment, preliminary metabolite profiling and characterisation of S011806 in healthy subjects with human plasma in selected cohorts will be conducted.

For Part 3:

Observed C_{max} on Days 1 and 22, trough concentrations on Days 2, 8, 15, 22 and 29 will

be reported.

9.3.3.2 Urine PK (Part 2 only)

As an exploratory assessment, PK parameters for urine S011806 will be calculated as appropriate, including but not limited to:

Ae_{t1-t2}:	Amount of unchanged drug excreted in the urine collection interval from t1 to t2.
Ae:	Total amount of drug excreted unchanged in the urine over the entire period of sample collection, obtained by adding the amounts excreted over each collection interval.
CLr:	Renal clearance calculated as Ae(t'-t")/AUC(t'-t") where t'-t" is the longest interval of time during which Ae and AUC are both obtained.
Fe:	Fraction (in percentage) of drug excreted unchanged in urine.

Selected metabolites will also be relatively quantified in the urine PK samples collected in selected cohorts.

9.3.4 PD Analysis (Part 3 Only)

For Part 3 only, serum and skin biopsy PD parameters for S011806 will be tabulated and listed for the PD analysis set, as appropriate, including serum concentrations of inflammatory cytokines (e.g. IL-17A), and beta defensin-2 (BD-2) over time and gene expression of multiple IL-17 pathway related markers in skin before and after S011806 treatment. PD measurements and their percent change from baseline will be summarised by treatment and time point of collection using appropriate summary statistics. All PD analyses will be fully outlined in an analysis plan, as appropriate.

9.3.5 Clinical Activity Analysis (Part 3 only)

BSA, PASI, PGA and LSS data will be summarised and listed for the full analysis set. Summaries will present observed and change from baseline values by treatment and time

point of collection. Data recorded from photography of lesion sites will be listed for the Full Analysis Set. Potential drug exposure-response relationship based on PASI score will also be explored, with full details given in the SAP.

9.4 Interim Analysis

At the Sponsor’s request, unblinded safety and efficacy tables, figures, and data listings may be presented to the Development Leadership Team for the purposes of planning future clinical studies prior to database lock. Unblinding of anyone directly involved in the conduct of the study will not occur for the purpose of this interim analysis. Full details will be given in the Interim Analysis SAP.

Details of interim safety data reviews to be performed between each cohort are described in Section 6.6.

9.5 Handling of Missing Data Points

Full details of procedures for handling missing data will be provided in the SAP. Given the descriptive and exploratory nature of the study, missing data for safety and PK endpoints will not be imputed. Missing or BLQ PK concentrations will be handled as described in section 9.3.3. Missing dermatology data will not be imputed and analysis of these data will include all subjects randomised and treated. Summaries of worst post baseline values may be included to allow for missing data at individual time points.

9.6 Data Monitoring Committee (DMC)

No independent DMC is planned for this study.

10. Supporting Documentation and Operational Considerations

10.1 Appendix 1: Regulatory, Ethical, and Study Oversight Considerations

10.1.1 Regulatory and Ethical Considerations

- This study will be conducted in accordance with the protocol and with the following:
  - Consensus ethical principles derived from international guidelines including the Declaration of Helsinki and Council for International Organizations of Medical Sciences (CIOMS) International Ethical Guidelines
  - Applicable ICH Good Clinical Practice (GCP) Guidelines
  - Applicable laws and regulations
  - 20 July 2017 EMEA/CHMP/SWP/28367/07 Rev. 1 Committee for Medicinal Products for Human Use (CHMP) Guideline on strategies to identify and mitigate risks for first-in-human and early clinical trials with investigational medicinal products
  - 21 July 2011 EMEA/CHMP/EWP/192217/2009 Committee for Medicinal Products for Human Use (CHMP) Guideline on bioanalytical method validation
  - London, 20 January 2010 Doc. Ref.: CPMP/QWP/EWP/1401/98 Rev. 1 Guideline on the investigation of bioequivalence"
  - https://www.gov.uk/guidance/managing-clinical-trials-during-coronavirus-covid-19 guidance.
- The protocol, protocol amendments, ICF, Investigator Brochure, and other relevant documents (e.g., advertisements) must be submitted to an Independent Ethics Committee (IEC) by the Investigator and reviewed and approved by the IEC before the study is initiated.
- Any substantial amendments to the protocol will require IEC approval before implementation of changes made to the study design, except for changes necessary to eliminate an immediate hazard to study subjects/patients.
- The Investigator will be responsible for the following:
  - Providing written summaries of the status of the study to the IEC annually or more

frequently in accordance with the requirements, policies, and procedures established by the IEC

- Notifying the IEC of SAEs or other significant safety findings as required by IEC procedures
- Providing oversight of the conduct of the study at the site and adherence to requirements of 21 CFR, ICH guidelines, the IEC, European regulation 536/2014 for clinical studies (if applicable), and all other applicable local regulations.

10.1.2 Financial Disclosure

Investigators and sub-investigators will provide the Sponsor with sufficient, accurate financial information as requested to allow the Sponsor to submit complete and accurate financial certification or disclosure statements to the appropriate regulatory authorities. Investigators are responsible for providing information on financial interests during the course of the study and for 1 year after completion of the study.

10.1.3 Informed Consent Process

- The Investigator or his/her designee will explain the nature of the study to the subject or patient and answer all questions regarding the study.
- Subjects/patients must be informed that their participation is voluntary. Subjects, patients or their legally authorised representative will be required to sign a statement of informed consent that meets the requirements of 21 CFR 50, local regulations, ICH guidelines, General Data Protection Regulation (GDPR), and the IEC or study centre.
- The medical record must include a statement that written informed consent was obtained before the subject/patient performed any study procedures and the date the written consent was obtained. The authorised person obtaining the informed consent must also sign the ICF.
- Subjects/patients must be re-consented to the most current version of the ICF(s) during their participation in the study.
- An original signed ICF(s) must be provided to the subject, patient or their legally authorised representative.

- Subjects and patients who are rescreened are required to sign a new ICF.

10.1.4 Data Protection

- Subjects and patients will be assigned a unique identifier by the site. Any subject/patient records or datasets that are transferred to the Sponsor will contain the identifier only; subject/patient names or any information which would make the subject/patient identifiable will not be transferred.
- The subject/patient must be informed that his/her personal study-related data will be used by the Sponsor in accordance with local data protection law. The level of disclosure must also be explained to the subject/patient who will be required to give consent for their data to be used as described in the informed consent.
- The subject/patient must be informed that his/her medical records may be examined by Clinical Quality Assurance auditors or other authorised personnel appointed by the Sponsor, by appropriate IEC members, and by inspectors from regulatory authorities.

10.1.5 Committees Structure

Data reviews during the study will be performed by the Safety Advisory Committee (SAC). See Section 6.6 for details of the committee structure.

10.1.6 Dissemination of Clinical Study Data

A clinical study summary report will be provided to the appropriate IEC and results uploaded to a clinical trials register within one year of the end of the clinical study.

10.1.7 Data Quality Assurance

- All subject and patient data relating to the study will be recorded on an electronic CRF unless transmitted to the Sponsor or designee electronically (e.g., laboratory data). The Investigator is responsible for verifying that data entries are accurate and correct by electronically signing the eCRF.
- The Investigator must maintain accurate documentation (source data) that supports the information entered in the eCRF.
- The Investigator must permit study-related monitoring, audits, IEC review, and

regulatory agency inspections and provide direct access to source data documents.

- Monitoring details describing strategy (e.g., risk-based initiatives in operations and quality such as Risk Management and Mitigation Strategies and Analytical Risk-Based Monitoring), methods, responsibilities and requirements, including handling of noncompliance issues, protocol deviations and monitoring techniques (central, remote, or on-site monitoring) are provided in the Monitoring Plan and/or other study-specific plans.
- The Sponsor or designee is responsible for the data management of this study including quality checking of the data.
- The Sponsor assumes accountability for actions delegated to other individuals (e.g., Contract Research Organizations).
- Study monitors will perform ongoing source data verification to confirm that data entered into the eCRF by authorised site personnel are accurate, complete, and verifiable from source documents; that the safety and rights of subjects and patients are being protected; and that the study is being conducted in accordance with the currently approved protocol and any other study agreements, ICH GCP, and all applicable regulatory requirements.
- Records and documents, including signed ICFs, pertaining to the conduct of this study must be retained by the Investigator for 15 years after study completion unless local regulations or institutional policies require a longer retention period. No records may be destroyed during the retention period without the written approval of the Sponsor. No records may be transferred to another location or party without written notification to the Sponsor.

10.1.8 Source Documents

- Source documents provide evidence for the existence of the subject/patient and substantiate the integrity of the data collected. Source documents are filed at the Investigator's site.
- Data reported on the CRF or entered in the eCRF that are transcribed from source documents must be consistent with the source documents or the

discrepancies must be explained. The Investigator may need to request previous medical records or transfer records, depending on the study. Also, current medical records must be available.

- Definition of what constitutes source data can be found in source data verification agreement (or equivalent).

10.1.9 Study and Site Closure

The Sponsor reserves the right to close the study site or terminate the study at any time for any reason at the sole discretion of the Sponsor. Study sites will be closed upon study completion. A study site is considered closed when all required documents and study supplies have been collected and a study-site closure visit has been performed.

The Investigator may initiate study-site closure at any time, provided there is reasonable cause and sufficient notice is given in advance of the intended termination.

Reasons for the early closure of a study site by the Sponsor or Investigator may include but are not limited to:

- Failure of the Investigator to comply with the protocol, the requirements of the IEC or local health authorities, the Sponsor's procedures, or GCP guidelines
- Inadequate recruitment of subjects and patients by the Investigator
- Discontinuation of further study IMP development by the Sponsor

If the study is prematurely terminated or suspended, the Sponsor shall promptly inform the Investigators, the IECs, the regulatory authorities, and any contract research organisation(s) involved in the study of the reason for termination or suspension, as specified by the applicable regulatory requirements. The Investigator shall promptly inform the subject/patient and should assure appropriate subject/patient therapy and/or follow-up.

10.2 Appendix 2: Clinical Laboratory Tests

<table>
<tr><th>Haematology</th><th>Clinical Chemistry</th><th>Virology</th><th>Urine Drugs of Abuse</th></tr>
<tr><td>Full blood count with differential:

Red Blood Cell (RBC) Count
Mean Cell Haemoglobin (MCH)
Mean Cell Haemoglobin Concentration (MCHC)
Mean Cell Volume (MCV)
Haematocrit (Packed Cell Volume- PCV)
Haemoglobin
Platelet Count

White Blood Cell (WBC) Count
Neutrophils
Eosinophils
Lymphocytes
Monocytes
Basophils

ESR</td><td rowspan="3">Alanine Aminotransferase (ALT)
Albumin
Alkaline Phosphatase
Aspartate Aminotransferase (AST)
Bicarbonate
Bilirubin (Total)
Bilirubin (Direct) (only if Total is elevated)
Calcium
Creatinine
Follicle Stimulating Hormone
(FSH; may be performed for post-menopausal female subjects to confirm post menopausal status at discretion of investigator)
hCG (all female subjects of child bearing potential at screen and follow up)
Gamma Glutamyl Transferase (GGT)
Glucose - Serum Glucose (Fasting)
Lactate dehydrogenase (LDH)
Potassium
Phosphate (Inorganic)
Protein (Total)
Sodium
Urea
eGFR
Serum lipids (fasting):
Total cholesterol
Triglycerides
HDL cholesterol
LDL cholesterol
CRP
Creatinine Kinase</td><td>Hepatitis B Surface Antigen
Hepatitis B Surface Antibody
Hepatitis B Core Antibody
Hepatitis C Virus Antibody
Hepatitis C Virus RNA (only if Hepatitis C Antibody positive)
HIV Antibody
Test for SARS-COV-2 (RT-PCR; throat swab)<hr>Quantiferon TB</td><td rowspan="3">Amphetamines
Barbiturates
Benzodiazepines
Cocaine
Marijuana/Cannabis
Methadone
Methamphetamine/ Ecstasy
Morphine/ Opiates
Phencyclidine
Tricyclic Antidepressants

Cotinine (Parts 1 and 2 only)</td></tr>
<tr><th>Coagulation</th><th>Urinalysis</th></tr>
<tr><td>Prothrombin time (PT)
Activated partial thromboplastin time (aPTT)
International normalised ration (INR)</td><td>Blood
Glucose
Leukocytes
Protein
Nitrites
If urinalysis is positive for protein, blood, nitrite and/or leukocytes, a microscopic examination (for red blood cells, white blood cells, bacteria, casts, and epithelial cells) will be performed.</td></tr>
</table>

10.3 Appendix 3: Adverse Events and Serious Adverse Events: Definitions and Procedures for Recording, Evaluating, Follow-up, and Reporting

10.3.1 Definition of AE

AE Definition
• An AE is any untoward medical occurrence in a patient or clinical study subject, temporally associated with the use of study IMP, whether or not considered related to the study IMP.<br>• NOTE: An AE can therefore be any unfavourable and unintended sign (including an abnormal laboratory finding), symptom, or disease (new or exacerbated) temporally associated with the use of study IMP.

Events Meeting the AE Definition
• Any abnormal laboratory test results (haematology, clinical chemistry, or urinalysis) or other safety assessments (e.g., ECG, radiological scans, vital signs measurements), including those that worsen from baseline, considered clinically significant in the medical and scientific judgment of the Investigator (i.e., not related to progression of underlying disease).<br>• Exacerbation of a chronic or intermittent pre-existing condition including either an increase in frequency and/or intensity of the condition.<br>• New conditions detected or diagnosed after study IMP administration even though it may have been present before the start of the study.<br>• Signs, symptoms, or the clinical sequelae of a suspected drug-drug interaction.<br>• Signs, symptoms, or the clinical sequelae of a suspected overdose of either study IMP or a concomitant medication. Overdose per se will not be reported as an AE/SAE unless it is an intentional overdose taken with possible suicidal/self-harming intent. Such overdoses should be reported regardless of sequelae.

Events NOT Meeting the AE Definition
• Any clinically significant abnormal laboratory findings or other abnormal safety assessments which are associated with the underlying disease, unless judged by the investigator to be more severe than expected for the subject/patient's condition.<br>• The disease/disorder being studied or expected progression, signs, or symptoms of the disease/disorder being studied, unless more severe than expected for the subject/patient's condition.<br>• Medical or surgical procedure (e.g., endoscopy, appendectomy): the

condition that leads to the procedure is the AE.<br>• Situations in which an untoward medical occurrence did not occur (social and/or convenience admission to a hospital).<br>• Anticipated day-to-day fluctuations of pre-existing disease(s) or condition(s) present or detected at the start of the study that do not worsen.

10.3.2 Definition of SAE

If an event is not an AE per definition above, then it cannot be an SAE even if serious conditions are met (e.g., hospitalisation for signs/symptoms of the disease under study, death due to progression of disease).

**A Serious Adverse Event is defined as any untoward medical occurrence that, at any dose:**
**a. Results in death**
**b. Is life-threatening**<br>The term 'life-threatening' in the definition of 'serious' refers to an event in which the subject/patient was at risk of death at the time of the event. It does not refer to an event, which hypothetically might have caused death, if it were more severe.
**c. Requires inpatient hospitalisation or prolongation of existing hospitalisation**<br>• In general, hospitalisation signifies that the subject/patient has been detained (usually involving at least an overnight stay) at the hospital or emergency ward for observation and/or treatment that would not have been appropriate in the physician's office or outpatient setting. Complications that occur during hospitalisation are AEs. If a complication prolongs hospitalisation or fulfils any other serious criteria, the event is serious. When in doubt as to whether "hospitalisation" occurred or was necessary, the AE should be considered serious.<br>• Hospitalisation for elective treatment of a pre-existing condition that did not worsen from baseline is not considered an AE.
**d. Results in persistent disability/incapacity**<br>• The term disability means a substantial disruption of a person's ability to conduct normal life functions.<br>• This definition is not intended to include experiences of relatively minor medical significance such as uncomplicated headache, nausea, vomiting, diarrhea, influenza, and accidental trauma (e.g., sprained ankle) which may interfere with or prevent everyday life functions but do not constitute a substantial disruption.

**e. Is a congenital anomaly/birth defect**
**f. Other situations:**<br>• Medical or scientific judgment should be exercised in deciding whether SAE reporting is appropriate in other situations such as important medical events that may not be immediately life-threatening or result in death or hospitalisation but may jeopardise the subject/patient or may require medical or surgical intervention to prevent one of the other outcomes listed in the above definition. These events should usually be considered serious.<br>• Examples of such events include invasive or malignant cancers, intensive treatment in an emergency room or at home for allergic bronchospasm, blood dyscrasias or convulsions that do not result in hospitalisation, or development of drug dependency or drug abuse.

10.3.3 Recording and Follow-Up of AE and/or SAE

**AE and SAE Recording**
• When an AE/SAE occurs, it is the responsibility of the Investigator to review all documentation (e.g., hospital progress notes, laboratory reports, and diagnostics reports) related to the event.<br>• The Investigator will then record all relevant AE/SAE information in the CRF.<br>• It is **not** acceptable for the Investigator to send photocopies of the subject/patient's medical records to the Sponsor in lieu of completion of the AE/SAE CRF page.<br>• There may be instances when copies of medical records for certain cases are requested by the Sponsor. In this case, all subject/patient identifiers, with the exception of the subject/patient number, will be redacted on the copies of the medical records before submission to the Sponsor.<br>• The Investigator will attempt to establish a diagnosis of the event based on signs, symptoms, and/or other clinical information. Whenever possible, the diagnosis (not the individual signs/symptoms) will be documented as the AE/SAE.
**Assessment of Intensity**
The Investigator will make an assessment of intensity for each AE and SAE reported during the study and assign it to 1 of the following categories:<br>• Mild: An event that is easily tolerated by the subject/patient, causing minimal discomfort and not interfering with everyday activities.<br>• Moderate: An event that causes sufficient discomfort and interferes with normal everyday activities.<br>• Severe: An event that prevents normal everyday activities. An AE that is assessed as severe should not be confused with a SAE. Severe is a category utilised for rating the intensity of an event; and both AEs and SAEs can be assessed as severe.

An event is defined as 'serious' when it meets at least 1 of the predefined outcomes as described in the definition of an SAE, NOT when it is rated as severe (i.e. severity should not be confused with seriousness).

Assessment of Causality

- The Investigator is obligated to assess the relationship between study IMP and each occurrence of each AE/SAE. The relationship should be classified as follows:
  - **Unlikely related**: a causal relationship between the study IMP and the AE is not a reasonable possibility
  - **Possibly related:** The event has a suggestive temporal relationship to the study IMP, and an alternative aetiology is equally or less likely.
  - **Probably related:** The event has a strong temporal relationship to the study IMP or recurs on re-challenge, and another aetiology is unlikely or significantly less likely.
- An AE is considered causally related to the use of the study IMP when the causality assessment is probably or possibly related.
- A "reasonable possibility" of a relationship conveys that there are facts, evidence, and/or arguments to suggest a causal relationship, rather than a relationship cannot be ruled out.
- The Investigator will use clinical judgment to determine the relationship.
- Alternative causes, such as underlying disease(s), concomitant therapy, and other risk factors, as well as the temporal relationship of the event to study IMP administration will be considered and investigated.
- The Investigator will also consult the Investigator's Brochure (IB) and/or Product Information, for marketed products, in his/her assessment.
- For each AE/SAE, the Investigator **must** document in the medical notes that he/she has reviewed the AE/SAE and has provided an assessment of causality.
- There may be situations in which an SAE has occurred and the Investigator has minimal information to include in the initial report to the Sponsor. However, it is very important that the Investigator always make an assessment of causality for every event before the initial transmission of the SAE data to the Sponsor.
- The Investigator may change his/her opinion of causality in light of follow-up information and send a SAE follow-up report with the updated causality assessment.
- The causality assessment is one of the criteria used when determining regulatory reporting requirements.

Follow-up of AEs and SAEs
• The Investigator is obligated to perform or arrange for the conduct of supplemental measurements and/or evaluations as medically indicated or as requested by the Sponsor to elucidate the nature and/or causality of the AE or SAE as fully as possible. This may include additional laboratory tests or investigations, histopathological examinations, or consultation with other health care professionals.<br>• If a subject or patient dies during participation in the study or during a recognised follow-up period, the Investigator will provide the Sponsor with a copy of any post-mortem findings including histopathology.<br>• New or updated information will be recorded in the originally completed CRF.<br>• The Investigator will submit any updated SAE data to the Sponsor within 24 hours of receipt of the information.

10.3.4 Reporting of SAEs/Pregnancies

SAE/Pregnancy Reporting to Sponsor via Paper Report Form
• e-mail transmission of the SAE paper form is the preferred method to transmit this information to Diamond Pharma Services PV, the Sponsor and the Medical Monitor with Facsimile as an alternative method if required.<br>• In rare circumstances and in the absence of working e-mail or facsimile equipment, notification by telephone is acceptable with a copy of the SAE data collection tool sent by overnight mail or courier service.<br>• Initial notification via telephone does not replace the need for the Investigator to complete and sign the SAE form within the designated reporting time frames.<br>• Contacts for SAE/Pregnancy reporting can be found on protocol page 4 (Diamond Pharma Services, the Sponsor and Medical Monitor contact details).

10.4 Appendix 4: Contraceptive Guidance, Exposure, Sperm Donation and Collection of Pregnancy Information

10.4.1 Contraception

Male subjects/patients who are sexually active with a partner of childbearing potential must use, with their partner, a condom plus an approved method of highly effective contraception from the time of informed consent until 90 days after their last dose of IMP.

The following methods are acceptable:

Highly Effective Methods of Contraception (to be used by male subjects/patients and their partners)

- Combined (estrogen and progestogen-containing) hormonal contraception associated with inhibition of ovulation:
  − oral
  − intravaginal
  − transdermal
- Progestogen-only hormonal contraception associated with inhibition of ovulation*:
  − oral
  − injectable
  − implantable
- Intrauterine hormone-releasing system (IUS)
- Implantable intrauterine device (IUD)
- Surgical sterilisation (for example, vasectomy** or bilateral tubal occlusion/ligation)

*The following are not considered highly effective, i.e. not associated with inhibition of ovulation: Micronor, Norgeston, Noriday.

**For vasectomy, the procedure should be documented in the subject/patients' medical history.

Female subjects/patients who are sexually active and of childbearing potential must use, with their partner, a condom plus an approved method of highly effective contraception from the time of informed consent until 90 days after their last dose of IMP.

<u>Highly Effective Methods of Contraception (to be used by female subjects of childbearing potential)</u>

- Combined (estrogen and progestogen-containing) hormonal contraception associated with inhibition of ovulation:
  − oral
  − intravaginal
  − transdermal
- Progestogen-only hormonal contraception associated with inhibition of ovulation*
  − oral
  − injectable
  − implantable
- IUS
- IUD
- Surgical sterilisation (for example, bilateral tubal occlusion/ligation)

*The following are not considered highly effective, i.e. not associated with inhibition of ovulation: Micronor, Norgeston, Noriday.

Alternatively, true abstinence is acceptable when it is in line with the subject/patient's preferred and usual lifestyle. If a subject/patient is usually not sexually active but becomes active, they, with their partner, must comply with the contraceptive requirements detailed above.

Female subjects/patients who are not of childbearing potential do not need to use any methods of contraception. A woman is considered of childbearing potential unless post-menopausal or permanently sterile. Permanent sterilisation methods include hysterectomy, bilateral salpingectomy, bilateral oophorectomy. A post-menopausal state is defined as no menses for 12 months without an alternative medical cause (without hormone replacement therapy [HRT]). If required by the Investigator, this may

be confirmed by a follicle stimulating hormone (FSH) result of ≥40 IU/L.

10.4.2 Exposure to Partners during the Study

There is a risk of drug exposure through the ejaculate (which also applies to vasectomised males) that might be harmful to the sexual partners (both male and female), including pregnant partners of male subjects/patients. Therefore, a condom should be used by all male subjects/patients throughout the study and for 90 days after their last dose of IMP.

10.4.3 Sperm and Ova/oocytes Donation

Male subjects/patients should not donate sperm for the duration of the study and for at least 90 days after their last dose of IMP.

Female subjects/patients should not donate ova/oocytes for the duration of the study and for at least 90 days after their last dose of IMP.

10.4.4 Collection of Pregnancy Information

Male subjects/patients with partners who become pregnant:

- The Investigator will attempt to collect pregnancy information on any male subject/patient's female partner who becomes pregnant while the male subject/patient is in this study. This applies only to male subjects/patients who receive IMP. The initial information will be recorded on the appropriate form and submitted to the Sponsor via PPD within 24 hours of learning of the pregnancy.
- After obtaining the necessary signed informed consent from the pregnant female partner directly, the Investigator will record pregnancy information on the appropriate form and submit it to the Sponsor. The female partner will also be followed to determine the outcome of the pregnancy. Information on the status of the mother and child will be forwarded to the Sponsor. Generally, the follow-up will be no longer than 6 to 8 weeks following the estimated delivery date. Any termination of the pregnancy will be reported regardless of foetal status (presence or absence of anomalies) or indication for the procedure.

Female subjects/patients who become pregnant:

- The Investigator will collect pregnancy information on any female subject/patient who becomes pregnant while participating in this study. The initial information will be recorded on the appropriate form and submitted to the Sponsor via PPD within 24 hours of learning of a subject/patient's pregnancy.
- The subject/patient will be followed to determine the outcome of the pregnancy after signature of a specific informed consent for pregnancy follow-up. The Investigator will collect follow-up information on the subject/patient and the neonate and the information will be forwarded to the Sponsor. Generally, follow-up will not be required for longer than 6 to 8 weeks beyond the estimated delivery date. Any termination of pregnancy will be reported, regardless of foetal status (presence or absence of anomalies) or indication for the procedure.
- While pregnancy itself is not considered to be an AE or SAE, any pregnancy complication or elective termination of a pregnancy for medical reasons will be reported as an AE or SAE.
- A spontaneous abortion (occurring at <22 weeks gestational age) or still birth (occurring at >22 weeks gestational age) is always considered to be an SAE and will be reported as such.
- Any post-study pregnancy-related SAE considered related to IMP by the Investigator will be reported to the Sponsor as described in Section 10.3.4. While the Investigator is not obligated to actively seek this information in former study subjects or patients, he or she may learn of an SAE through spontaneous reporting.
- Any female subject/patient who becomes pregnant while participating in the study will be withdrawn from IMP. A male subject/patient will be withdrawn from IMP in the event that his female partner becomes pregnant.

All pregnancies during the study will be reported to PPD, the Sponsor and the Medical Monitor (contact details on protocol page 4).

10.5 Appendix 5: Summary of Protocol Changes

**Document History**	
**Document**	**Date**
Amendment 04	11 February 2022
Amendment 03	14 December 2021
Amendment 02	28 October 2021
Amendment 01	03 September 2021
Original Protocol	24 May 2021

Amendment 04, 11 February 2022:

This amendment is considered to be substantial based on the criteria set forth in Article 10(a) of Directive 2001/20/EC of the European Parliament and the Council of the European Union.

Overall Rationale for the Amendment:

This protocol amendment is written to update the exclusion criteria and permitted/prohibited medications for psoriasis patients in Part 3 of the study. These changes are made to aid the recruitment of psoriasis patients with no impact on patient safety or scientific integrity. Clarifications are made to the 'Statistical Considerations' text. Minor typographical errors are also corrected.

**Section # and Name**	**Description of Change**	**Brief Rationale**
Section 1.1 Synopsis	Text removed from interim data review section	To align with text updates to Section 9 of the protocol.
Section 1.2.8 Part 3 Daily Schedule	Row for urine cotinine testing removed<br><br>Footnote p added to C-SSRS on Day 8 and 15.<br><br>X added to Day 2 IMP dispensing/accountability<br><br>X removed from Day 1 IMP dosing at home	In line with changes to smoking related exclusion criteria, the requirement for cotinine testing has been removed<br><br>Footnote p is added to indicate the C-SSRS should be pre-dose on Days 8 and 15.

		A residential period from Day 1 to Day 2 was previously added to the protocol. IMP dispensing/accountability is therefore required upon discharge on Day 2 and home dosing is not required on Day 1. Changes to the SoA have been made to reflect this.
Section 5.2.2 Exclusion Criteria for Patients with Psoriasis (Part 3)	Exclusion criteria 26, 27 and 28 removed:<br><br>Current or previous use of tobacco, nicotine products or e-cigarettes in the past 6 months.<br><br>Smoking history of >10 pack years.<br><br>Positive urine cotinine test at screening or Day 1.	These criteria have been removed to allow patients with psoriasis who smoke to be included to aid recruitment to the psoriasis population.
Section 5.5.3. Permitted Concomitant Medication for Patients with Psoriasis (Part 3)	Limitation of 2000mg per day has been removed from paracetamol.<br><br>The following text has been added:<br>In addition, other concomitant medications taken at a stable dose for at least 3 months prior to screening which are not listed in Section 5.5.5 may be permitted at the Investigators discretion following discussion with the Medical Monitor.	These changes to permitted medication for psoriasis patients in Part 3 have been made to aid recruitment to the psoriasis population.
Section 5.5.5. Prohibited Concomitant Medication for Patients with Psoriasis (Part 3)	The following text has been removed:<br>With the exception of those listed in Section	These changes to prohibited medication for psoriasis patients in Part 3 have been made to aid

	5.5.3, the use of any prescribed or non-prescribed medication is prohibited within 14 days of IMP administration until after the last study visit (follow-up visit). This includes:<br>• NSAIDs (including coxibs and aspirin)<br>• Herbal remedies, vitamin supplements and minerals<br>• Other over-the counter (OTC) medicines<br><br>The following text has been added in relation to topical medications/treatments: within 14 days of IMP administration until after the last study visit (follow up visit).<br><br>The following text has been added: NSAIDs (including coxibs and aspirin) and anticoagulants within 14 days of IMP administration until after the last study visit (follow up visit).<br><br>Reference to strong CYP3A inhibitors or inducers is changed to 'strong or moderate'.	recruitment to the psoriasis population.<br><br>Preliminary results on enzyme identification suggested that CYP3A isoforms are likely the primary enzymes responsible for the metabolism of S011806. Therefore, strong or moderate inducers of CYP3a will still be prohibited in Part 3.
Section 5.5.10 Additional restrictions, Section 8.10.7.6 Drug Screen and Urine Cotinine and Section 10.2 Clinical Laboratory Tests	'Part 1 and 2 only' added in reference to nicotine restrictions and cotinine testing.<br>In addition, the following has been added:	In line with the removal of smoking exclusion criteria for Part 3, restrictions and testing have been updated to reference Parts 1 and 2 only.

	'In Part 3, smoking is not permitted for 2 hours pre-dose until 4-hours post first dose on Day 1 and Day 22'.	
Section 9.2 Population for Analyses, Section 9.3.3.1 Plasma PK, Section 9.3.3.2 Urine PK (Part 2 only), Section 9.3.4 PD Analysis (Part 3 only) and Section 9.4 Interim Analysis	Minor text updates	Text updated to add clarity in line with current data analysis plans.

Amendment 03, 14 December 2021:

This amendment is considered to be substantial based on the criteria set forth in Article 10(a) of Directive 2001/20/EC of the European Parliament and the Council of the European Union.

Overall Rationale for the Amendment:

This protocol amendment is written to include the potential for interim analysis. In addition, the maximum number of subjects in Part 3 is increased. Potential exploratory metabolite profiling and analysis of plasma and urine PK samples is added. Clarification of skin biopsy requirements in the event of early termination is added and typographical errors from the previous amendment are corrected.

Section # and Name	Description of Change	Brief Rationale
Section 1.1 Synopsis	Text added to reflect changes in main text detailed below.	Updated in line with updates detailed below.
Section 1.2.8 Part 3 Daily Schedule	Serum biomarkers and skin biopsy added to FU/ET column and footnote r added to denote this is required in the event of ET prior to Day 29 only.<br><br>Reference to photography of lesion site removed from footnote q.	Serum biomarkers and skin biopsy are procedures from EOT which would be required in the event of ET, if it occurs prior to Day 29 (EOT). They are therefore added to the FU/ET column with a footnote to confirm when they are required.

		Updated as per protocol clarification letter number 03 dated 09 Dec 2021:<br><br>Photography of lesion site is on an individual row with different time points. Removed erroneous reference from footnote referencing dermatology assessments.
Section 2.2.1.2 Rationale for Dose Escalation and Maximum Dose in Part 1 (SAD)	NOAEL coverage values for C_{max} and AUC in relation to planned doses of 225mg and 450mg updated in Table 1.	Updated as per protocol clarification letter number 03 dated 09 Dec 2021:<br><br>225mg and 450mg in the planned dose column of table 1 was updated in the previous amendment. The corresponding NOAEL coverage values for C_{max} and AUC were not updated in error.
Section 3.3.1	The following exploratory objective has been added to Part 1:<br>'To potentially conduct preliminary metabolite profiling and characterisation of S011806 in healthy subjects with human plasma in selected cohorts'.	Existing exploratory objective for Part 2, reflected in Part 1.
Section 3.4.3 Exploratory Endpoints	Text added to state: 'Plasma and urine PK samples collected during Part 1 and Part 2 of the study may also be used for exploratory research, including but not limited to, metabolite profiling and identification'.	Existing text for Part 2 exploratory analysis. Text added to reflect this exploratory endpoint across part 1 and 2.
Section 4.1Overall Study Design, Section 4.4 Part 3 (Psoriasis Patients) and Section 9.1 Sample Size Determination	Text updated to increase the maximum number of subjects in Part 3 from 30 to 42. Text now indicates up to 42 (a minimum of	Maximum number of patients recruited to Part 3 has been increased to enable a better understanding of safety

	15 up to a maximum of 21 per cohort). Corresponding per cohort number and reference to the active: placebo ratio updated.	and clinical activity.
Section 6.4.3 Procedure for Breaking the Blind Prior to Study Completion	Text added to confirm Unblinding of anyone directly involved in the conduct of the study will not occur for the purpose of the interim analysis.	Text added to confirm blinding will be maintained in the event of Interim Analysis.
Section 9.3.3 PK Analysis	Text added to state additional exploratory analysis including but not limited to metabolite identification and profiling may occur on collected PK samples.	Text added to clarify that this exploratory analysis will utilise existing plasma samples collected for PK.
Section 9.4 Interim Analysis	Text updated to detail an optional interim analysis.	Interim analysis may be performed for the purpose of planning future studies
Section 10.2 Appendix 2: Clinical Laboratory Tests	Cotinine added to the 'Urine Drugs of Abuse' Column.	Cotinine was removed during the previous amendment in error. It was not listed in duplicate so is re-added in this amendment for clarity and in line with section 8.10.7.6 'Drug Screen and Urine Cotinine'.

Amendment 02, 28 October 2021:

This amendment is considered to be substantial based on the criteria set forth in Article 10(a) of Directive 2001/20/EC of the European Parliament and the Council of the European Union.

Overall Rationale for the Amendment:

This protocol amendment is written to update the planned doses for Part 1. Updates to the fasting requirements for Part 2 are also included and typographical errors corrected.

In addition, two protocol clarification letters; Protocol Clarification Letter number 01,

dated 13 September 2021 and Protocol Clarification Letter 02, dated 18 October 2021 have been issued since the previous protocol amendment and the changes have been incorporated into the protocol within Amendment 02 with the description of change and brief rational listed in the table below.

Section # and Name	Description of Change	Brief Rationale
Section 1.1 Synopsis and Section 6.2 Administration of Investigational Medicinal Product	Text added to expand on the FDA requirements for water restrictions in the food effect cohort. In both treatment periods, water is not permitted for 1 hour prior to, or post-dose with the exception of the water required to swallow tablets. In addition, a drink is permitted with breakfast in the fed cohort.	Updated as per protocol clarification letter number 02 dated 18 Oct 2021:<br><br>Clarified in line with the referenced FDA requirements on water restrictions for food effect cohorts.
Section 1.2.1 Part 1 Daily Schedule	Footnote m updated to clarify that serum pregnancy is required in the event of an early termination visit	Updated as per protocol clarification letter number 02 dated 18 Oct 2021:<br><br>Pregnancy testing at Early Termination should mirror Follow up.
Section 1.2.2 Part 1 Daily Schedule – Food Effect Cohort	Footnote m updated to clarify that serum pregnancy is required in the event of an early termination visit and on Day 8 of TP 1.<br><br>Footnote p has moved from the PK row to the IMP row and reference to Section 6.2 added.<br><br>Footnote r updated to remove reference to Day 7 and add Day 8.	Updated as per protocol clarification letter number 02 dated 18 Oct 2021:<br><br>Pregnancy testing at Early Termination and end of first treatment period should mirror Follow up.<br><br>Updated as per protocol clarification letter number 01 dated 13 Sept 2021 and 02 dated 18 Oct 2021:<br><br>Typographical errors updated.
Section 1.2.3 Part 1 Hourly Schedule	Reference to serum removed from pregnancy	Typographical error corrected. As per footnote

	testing row title.	and section 8.10.7.4, urine pregnancy testing will be performed prior to dose on Day 1.
1.2.4 Part 2 Daily Schedule - 7-day dosing and 1.2.5 Part 2 Daily Schedule – 10-day dosing.	Weight removed from Day -1 and added to Day 1 and Follow Up/Early Termination visit.<br><br>Footnote n updated to clarify that serum pregnancy is required in the event of an early termination visit.<br><br>Footnote p updated to clarify urine collection times in relation to Day 2 dose.	Updated as per protocol clarification letter number 01 dated 13 Sept 2021:<br><br>In line with other parts, weight is to be measured pre-dose on Day 1, not Day -1.<br><br>Updated as per protocol clarification letter number 02 dated 18 Oct 2021:<br><br>Weight should be measured in the event of early termination. It is therefore added to the Follow Up/Early Termination visit.<br><br>Pregnancy testing at Early Termination should mirror Follow up.<br><br>Updated as per protocol clarification letter number 01 dated 13 Sept 2021:<br><br>Urine collection should end prior to Day 2 dose and a new 12-hour collection begin. Per Section 8.13.2, further details of urine PK collection are included in the SRM.
1.2.6 Part 2 Hourly Schedule Days 1 - 2	Erroneous 0hr Pk removed<br><br>Pre-dose Day 1 weight added<br><br>Row title for Haematology and serum chemistry updated to Haematology, Serum Chemistry and	Updated as per protocol clarification letter number 01 dated 13 Sept 2021:<br><br>Typographical error corrected. 0hr PK is not required.<br><br>Pre-dose weight added in line with the changes to

	Coagulation.<br><br>Day 2 column headers updated from 24h, 36h, 48 hr to 0h (D1 24h), 8h and 12h respectively.<br><br>Reference to serum removed from the pregnancy testing row title.<br><br>Footnote k updated to clarify urine collection times in relation to Day 2 dose.	the Daily Schedule in Sections 1.2.4 and 1.2.5.<br><br>Row title updated in line with Daily Schedule in Sections 1.2.4 and 1.2.5.<br><br>Column headers updated to clarify procedure time points for Day 2 are in relation to Day 2 dose, not Day 1 dose.<br><br>Typographical error corrected. As per footnote and section 8.10.7.4, urine pregnancy testing will be performed prior to dose on Day 1.<br><br>Urine collection should end prior to Day 2 dose and a new 12-hour collection begin. Per Section 8.13.2, further details of urine PK collection are included in the SRM.
1.2.7 Part 2 Hourly Schedule Days 7 – 9 or 10 to 12.	ECG and Vital signed removed from 1.5h time point and added to 1h time point<br><br>Footnote d updated to remove reference to triplicate ECG.<br><br>Footnote f updated to removed reference to Days 1 – 2 and replace with Day 7 or 10 and Day 8 or 11.	Updated as per protocol clarification letter number 01 dated 13 Sept 2021:<br><br>Typographical error corrected. Vitals and ECG should be performed 1h post-dose not 1.5h.<br><br>Typographical error updated. In line with Daily Schedules in Section 1.2.4 and 1.2.5 and Section 8.10.4, triplicate ECG is only required pre-dose on Day 1.<br><br>Typographical error updated to reference the correct days of the corresponding schedule.
1.2.8 Part 3 Daily	Footnote p, denoting pre-	Updated as per protocol

Schedule	dose, added to weight on Day 1.<br><br>Footnote m updated to clarify that serum pregnancy is required in the event of an early termination visit.	clarification letter number 01 dated 13 Sept 2021:<br><br>Footnote updated for clarity of the timing of weight measurement on Day 1.<br><br>Updated as per protocol clarification letter number 02 dated 18 Oct 2021:<br><br>Pregnancy testing at Early Termination should mirror Follow up.
1.2.9 Part 3 Hourly Schedule Days 1 and 22	1.5h and 3h columns removed.<br><br>Footnote d updated to clarify pre-dose is Day 1.	Updated as per protocol clarification letter number 01 dated 13 Sept 2021:<br><br>Typographical error corrected. 1.5h and 3h columns isobsolete.<br><br>In line with the Daily Schedule in Section 1.2.8 and Section 8.10.4, triplicate ECG is only required pre-dose on Day 1.
Section 2.2.1.2 Rationale for Starting Doses in Part 1 [Single Ascending Dose (SAD)]	Table 1 is updated to increase planned dose 3 from 200mg to 225mg and dose 4 from 400mg to 450mg. The footnote of Table 1 is also updated to clarify that changes in dosing based on available human PK and safety data will not exceed 3-fold escalation and a maximum of 800mg rather than not exceeding the planned dose.	Planned doses 3 and 4 are increased within the maximum 3-fold escalation advised by the MHRA.<br><br>This increase is to enable more flexibility in Part 2 (MAD) starting doses i.e. Part 2 Cohort 1 may start at 200mg and Cohort 2 at 400mg.<br><br>Actual doses in all Parts remain the decision of the SAC based on the Safety and PK data from completed cohorts per Section 6.6.

		The footnote is updated in line with the MHRA recommendations of maximum fold escalation and maximum highest dose.
4.1 Overall Study Design	Figure 1 is updated to increase planned dose 3 from 200mg to 225mg and planned dose 4 from 400mg to 450mg.	Figure updated to align with Table 1 in Section 2.2.1.2.
4.2 Part 1 (Healthy Subject SAD) and Section 6.6 Criteria for Dose Escalation (Parts 1 and 2)	Reference to modification in SAD doses is changed from:<br>'shall not exceeed planned doses' to ' shall not exceed 3-fold escalation and a maximum dose of 800mg'	This text is updated in line with the MHRA recommendations of maximum fold escalation and maximum highest dose and to align with Table 1 in Section 2.2.1.2.
Section 5.2.1 Exclusion Criteria for Healthy Subjects (Part 1 and 2), Section 5.2.2 Exclusion Criteria for Patients with Psoriasis (Part 3) and Section 8.10.7.9 Columbia Suicide Severity Scale (C-SSRS)	Exclusion criteria 35 and 9 in Section 5.2.1 and 5.2.2 respectively and text in Section 8.10.7.9 have been updated to reference the 'Baseline/Screening' version of the Columbia Suicide Severity Rating Scale (C-SSRS) rather than the 'Screening' version.	Updated as per protocol clarification letter number 01 dated 13 Sept 2021:<br><br>Typographical error corrected.
Section 5.5.10 Additional Restrictions	Clarification added in reference to the 2-treatment period food effect cohort.	Updated as per protocol clarification letter number 02 dated 18 Oct 2021:<br><br>Updated to clarify 'last dose' refers to each individual dose in the food effect cohort.
Section 6.2 Administration of the Investigational Medicinal Product	The following text has been added: Fasting windows may be adjusted depending on emerging PK data. Modifications to fasting windows are permitted to allow volunteers to eat 3 times	In addition to existing text which permits flexibility to remove fasting requirements based on emerging PK, flexibility to modify the required fasting windows based on emerging PK has been

	per day.	added. Fasting may also be modified to ensure subjects are able to eat 3 meals per day.
Section 8.10.7.4 Pregnancy Test	Reference to serum pregnancy testing at the Early Termination visit added.	Updated as per protocol clarification letter number 02 dated 18 Oct 2021:<br><br>Pregnancy testing at Early Termination should mirror Follow up.
Section 10.2 Appendix 2: Clinical Laboratory Tests	Duplication of cotinine removed from 'Urine drugs of abuse' column' ESR moved from 'Clinical Chemistry' to 'Haematology' column.	Typographical errors corrected

Amendment 01, 03 September 2021:

This amendment is considered to be substantial based on the criteria set forth in Article 10(a) of Directive 2001/20/EC of the European Parliament and the Council of the European Union.

Overall Rationale for the Amendment:

This protocol amendment is written to address the Medicines and Healthcare products Regulatory Agency (MHRA) comments with regards to subjects' renal function, dose escalation, additional safety monitoring, and stopping criteria. The following changes were made to follow these recommendations:

Section # and Name	Description of Change	Brief Rationale
Section 1.1 Synopsis	Updated to align with changes to the main document	This section is updated to align the synopsis to the main document

Section 1.2.1 Part 1 Daily Schedule,<br>Section 1.2.3 Part 1 Hourly Schedule	Schedule updated to change the time of subject discharge from Day 4 (i.e., 72 hours post dose) to Day 3 (i.e.,48 hours post dose).<br><br>In view of the above the abbreviated physical examination and ECG have now been moved to Day 3 (i.e.,48 hours post dose).<br><br>Footnote 'd' updated accordingly in Section 1.2.1.	This change has been made based on the predicted half-life of the investigational product and additional administrative changes
Section 1.2.2 Part 1 Daily Schedule – Food Effect Cohort	Schedule updated to change the time of subject discharge from Day 4 (i.e., 72 hours post dose) to Day 3 (i.e.,48 hours post dose) in both Treatment Period 1 and Treatment Period 2.<br><br>In view of the above the abbreviated physical examination and ECG have now been moved to Day 3 (i.e.,48 hours post dose) in both Treatment Period 1 and Treatment Period 2.<br><br>Footnote 'd' updated accordingly.	Changes made to align with advice from MHRA Additional, safety checks added
Section 1.2.2 Part 1 Daily Schedule – Food Effect Cohort	The follow up visits (in both Treatment Periods) have been changed from Day 7 (+/- 2 days) Day 8 (+/- 2 days).	Update to be in line with all other sections of the protocol that require the follow up periods to be 7 days (+/- 2 days) post last dose.

Section 1.2.4 Part 2 Daily Schedule - 7-day dosing,<br>Section 1.2.5 Part 2 Daily Schedule – 10-day dosing,<br>Section1.2.6<br>Part 2 Hourly Schedule Days 1 – 2,<br>Section 1.2.7 Part 2 Hourly Schedule Days 7 – 9 or 10 – 12	All schedules updated to include Columbia-Suicide Severity Rating Scale (C-SSRS) questionnaires at indicated timepoints.	Safety check as per MHRA guidance
Section 1.2.8 Part 3 Daily Schedule	Schedule updated to indicate that the subject is to remain resident overnight on Day 1 until after the morning dose on Day 2<br><br>ECG, Blood pressure, heart rate, safety bloods and safety urine have been added pre dose on Day 2<br><br>Columbia-Suicide Severity Rating Scale (C-SSRS) questionnaires added to all visits.<br><br>Covid-19 Screening questioning removed from Day 2 as the patients are now resident overnight on Day 1	Additional, safety checks added to align with MHRA guidance
Section 1.2.9 Part 3 Hourly Schedule Days 1 and 22,	Schedule updated to include Columbia-Suicide Severity Rating Scale (C-SSRS) questionnaires at timepoints as indicated<br><br>Updated to include timepoints for dosing at 8 hours, 12 hours and 16 hours (actual dosing depending on dosing regimen in place at the	Additional, safety checks added to align with MHRA guidance

	time) as the patient is now resident overnight.<br><br>Vital signs have been added to the 8 hr and 12-hour timepoints.<br><br>An Additional PK has been added to the 12-hour timepoint.<br><br>Additional footnotes included in line with the above.	
Section 2.2.1.2 Rationale for Dose Escalation and Maximum Dose in Part 1 (SAD)<br>Table 4. Planned S011806 Doses in Part 1 (SAD) Cohorts	Table 1:<br>Updated to change the planned doses and to to remove doses above 800 mg	Updates to remove the higher doses > 800 mg in SAD to align with MHRA guidance
Section 2.2.1.3 Rationale for Dose Levels and Maximum Dose in Part 2 [Multiple Ascending Dose (MAD)]	Updated to remove reference to 'somewhat higher than'	Aligned with MHRA guidance
Section 2.3 Benefit-Risk Assessment	Updated with rationale for changing the post dose residential observation period at site from 72 hours post dose to 48 hours post dose.	Given the projected human elimination half life is 4.5 hours, subjects will only be discharged from the unit ~~72 and~~ 48 hours (minimum of 5 half-lives) after the last dose ~~respectively,~~ and the Investigator deems it safe to do so. Aligned with MHRA guidance.
Section 2.3 Benefit-Risk Assessment	Updated to include the Columbia-Suicide Severity Rating Scale (C-SSRS) questionnaires	Aligned with MHRA guidance

Section 2.3 Benefit-Risk Assessment	Updated to include a discussion of the potential impact of S011806 on susceptibility to and severity of COVID-19 infection based on mechanism of action in addition to the current COVID-19 risk and mitigation information	covid -19 susceptibility added as per MHRA request
Section 3.4.1 Primary Endpoints (Safety)	Updated to include C-SSRS as a primary safety endpoint	Aligned with MHRA guidance
Section 4.1 Overall Study Design<br>Figure 2. Overall Study Design and Dose-escalation Scheme	Updated to include overnight residency on Day 1 to Day 2 for Part 3<br><br>Updated Figure 1 in line with the changes to planned doses	Further observations as per MHRA guidance
Section 4.2 Part 1 (Healthy Subject SAD)	Updated to reduce the number of cohorts from 6 to 5 and the number of subjects from 48 to 40.<br><br>Update to reduce the dose/exposure increment being no more than 3-fold<br><br>Updated to change subject discharge to Day 3 (i.e., 48 hours post last dose) from Day 4 (i.e..72 hours) post last dose.<br><br>Updated to state that residence at the CRU post-dose may be adjusted based on emerging data (minimum of 5 half-lives post dose).	Aligned with removal of the higher doses, and reduce the dose/exposure increment being no more than 3-fold.<br>Residential stay aligned with predicted half-life of the IMP per MHRA guidance

Section 4.2.1 Part 1 (Healthy Subject SAD): Food-Effect Cohort	Updated to state: The predicted exposure in the fed state at the selected dose should not exceed a dose/exposure as defined in the PK stopping criteria (Table 3) and must be agreed as acceptable by the safety advisory committee (SAC).	Updated to align with MHRA guidance
Section 4.3 Part 2 (Healthy Subject MAD)	Updated to state: Residence at the CRU after dose may be adjusted based on emerging data (minimum of 5 half-lives after final dose).<br><br>Updated to include reference to C-SSRS	Updated to align with MHRA guidance and administrative
Section 4.4 Part 3 (Psoriasis Patients)	Updated to include reference to overnight stay on Day 1 to Day 2	Updated to align with MHRA guidance and administrative
Section 4.4 Part 3 (Psoriasis Patients)	Updated with rationale for the extended follow up period (i.e. 2 weeks post last dose).	Updated to align with MHRA guidance
Section 5.1.1. Inclusion Criteria for Healthy Subjects (Parts 1 and 2)	Inclusion Number 4 and Inclusion No. 5 (contraception) updated to be in line with Section 10.4	Updated to align with MHRA guidance
Section 5.1.2. Inclusion Criteria for Patients with Psoriasis (Part 3)	Inclusion Number 8 and Inclusion No. 9 (contraception) updated to be in line with Section 10.4	Updated to align with MHRA guidance

Section 5.2.1 Exclusion Criteria for Healthy Subjects (Parts 1 and 2)	The serum creatinine criterion has been removed from Exclusion No. 4<br><br>An additional Exclusion Criteria (Exclusion No. 35) has been included in relation the presence of active suicidal ideation or positive suicide behaviour.	Updated to align with MHRA guidance
Section 5.2.2. Exclusion Criteria for Patients with Psoriasis (Part 3)	An additional Exclusion Criteria (Exclusion No. 9) has been included in relation the presence of active suicidal ideation or positive suicide behaviour.<br><br>The serum creatinine criterion has been removed from Exclusion No. 12.	Section updated to align with MHRA guidance
Section 5.5.10. Additional Restrictions	Sun-Exposure:<br>Additional restrictions<br>have been added for<br>Parts 1 and 2	Section updated to align with MHRA guidance
Section 6.2.2. At-home Administration of IMP (Part 3 only)	Updated to state that all doses on Day 1 will be taken in the unit as the patients are resident overnight	Administrative changes to align with MHRA guidance
Section 6.6 Criteria for Dose Escalation (Parts 1 and 2)	Updated with changes to planned doses and dosing increments	Section updated to align with MHRA guidance

Section 6.7 Stopping Criteria<br>Table 3. Study and Cohort Stopping Rules	Table 5. Study and Cohort Stopping Rules Updated:<br><br>Removed ‘as applicable’ from all relevant sections<br><br>**Severe Non-Serious:** Removed ‘in the same target organ’<br><br>**Added:**<br>PK/Exposure Stopping Criteria	Section updated to align with MHRA guidance
Section 8.5 Discharge from the Clinical Research Unit	Updated to include overnight residency on Day 1 for Part 3	Administrative changes to align with MHRA guidance
Section 8.10.7.9 Columbia Suicide Severity Rating Scale (C-SSRS)	New section added	Safety check as per Update align with MHRA guidance

References

1. *World Health Organisation, Global Report on Psoriasis*. 2016.
2. Michalek, I.M., B. Loring, and S.M. John, *A systematic review of worldwide epidemiology of psoriasis.* J Eur Acad Dermatol Venereol, 2017. **31**(2): p. 205-212.
3. Armstrong, A.W. and C. Read, *Pathophysiology, Clinical Presentation, and Treatment of Psoriasis: A Review.* JAMA, 2020. **323**(19): p. 1945-1960.
4. Langley, R.G., et al., *Secukinumab in plaque psoriasis--results of two phase 3 trials.* N Engl J Med, 2014. **371**(4): p. 326-38.
5. Gordon, K.B., et al., *Phase 3 Trials of Ixekizumab in Moderate-to-Severe Plaque Psoriasis.* N Engl J Med, 2016. **375**(4): p. 345-56.
6. Lebwohl, M., et al., *Phase 3 Studies Comparing Brodalumab with Ustekinumab in Psoriasis.* N Engl J Med, 2015. **373**(14): p. 1318-28.
7. Prinz, I., I. Sandrock, and U. Mrowietz, *Interleukin-17 cytokines: Effectors and targets in psoriasis-A breakthrough in understanding and treatment.* J Exp Med, 2020. **217**(1).
8. Fletcher, J.M., et al., *IL-17 in inflammatory skin diseases psoriasis and hidradenitis suppurativa.* Clin Exp Immunol, 2020. **201**(2): p. 121-134.
9. Kolbinger, F., et al., *beta-Defensin 2 is a responsive biomarker of IL-17A-driven skin pathology in patients with psoriasis.* J Allergy Clin Immunol, 2017. **139**(3): p. 923-932 e8.
10. Matusiak, L., et al., *Increased interleukin (IL)-17 serum levels in patients with hidradenitis suppurativa: Implications for treatment with anti-IL-17 agents.* J Am Acad Dermatol, 2017. **76**(4): p. 670-675.
11. Huppertz, C.C.D., M.; Hennze, R.; and Kolbinger, F., *The Anti-IL-17A Monoclonal Antibody Secukinumab (AIN457) Inhibits Pro-Inflammatory Mediator Release From Human Primary Synoviocytes Costimulated With IL-17 and TNF; Abstract number 951*, in *ACR/ARHP Annual Meeting*. 2013.
12. Baronaite Hansen, R. and A. Kavanaugh, *Secukinumab for the treatment of psoriatic arthritis.* Expert Rev Clin Immunol, 2016. **12**(10): p. 1027-36.
13. *Food and Drug Administration (FDA)Estimating the Maximum Safe Starting Dose in Initial Clinical Trials for Therapeutics in Adult Healthy Volunteers*. July 2015; Update August 2018.
14. Nair, A.B. and S. Jacob, *A simple practice guide for dose conversion between animals and human.* J Basic Clin Pharm, 2016. **7**(2): p. 27-31.
15. *Food and Drug Administration (FDA) Drug Development and Drug Interactions: Table of Substrates, Inhibitors and Inducers.* https://www.fda.gov/drugs/drug-interactions-labeling/drug-development-and-drug-interactions-table-substrates-inhibitors-and-inducers#table3-2.
16. *Food and Drug Administration (FDA) Food-Effect Bioavailability and Fed Bioequivalence Studies*. 2002.
17. *Joint United Kingdom (UK) Blood Transfusion and Tissue Transplantation Services Professional Advisory Committee Guidelines for the Blood Transfusion Services in the UK*. 2013.
18. *Guidelines for Phase I Clinical Trials. Association of the British Pharmaceutical Industry Guidelines. London, UK; 2012 Edition (as amended 12 Nov 2014).*
19. Kridin K. et al., Risk of COVID-19 Infection, Hospitalization, and Mortality in

Patients with Psoriasis Treated by Interleukin-17 Inhibitors, Dermatolog. 2021 Treat DOI: 10.1080/09546634.2021.1905766 PMID: 33759683
20. Talamonti M et al., Characteristic of chronic plaque psoriasis patients treated with biologics in Italy during the COVID-19 Pandemic: Risk analysis from the PSO-BIO-COVID observational study. Expert Opin Biol Ther. 2021 Feb;21(2):271-277. doi: 10.1080/14712598.2021.1853698. Epub 2021 Jan 13.
21. Avdeev SN et al., 2021, Anti-IL-17 monoclonal antibodies in hospitalized patients with severe COVID-19: A pilot study, Cytokine. 2021 Oct;146:155627. doi: 10.1016/j.cyto.2021.155627. Epub 2021 Jul 3.

Abbreviations

AE	Adverse Event
ALP	Alkaline Phosphatase
ALT	Alanine Aminotransferase
aPTT	Activated prothrombin time
AST	Aspartate Aminotransferase
AUC	Area Under the Serum Concentration-Time Curve
BD-2	Beta definsin-2
BID	bis in die (twice a day)
BLQ	Below Limit of Quantification
BMI	Body Mass Index
BP	Blood pressure
bpm	Beats per minute
BSA	Body Surface Area
CE-marked	Conformité Européene (European Conformity)
CFR	Code of Federal Regulations
CHMP	Committee for Medicinal Products for Human Use
CIA	Collagen-induced Arthritis
CIOMS	Council for International Organizations of Medical Sciences
CK	Creatine Kinase
C_{max}	Maximum Observed Concentration
COVID-19	Corona Virus Disease 2019
CRF	Case Report Form
CNS	Central Nervous System
CRO	Contract Research Organisation
CRU	Clinical Research Unit
CS	Clinically significant
C-SSRS	Columbia-Suicide Severity Rating Scale
CYP	Cytochrome P
DMC	Data Monitoring Committee
DNA	Deoxyribonucleic acid
EC	Ethics Committee
ECG	Electrocardiogram
eCRF	Electronic Case Report Form
eGFR	Estimated Glomerular Filtration Rate
EPO	erythropoietin
ET	Early Termination
EudraCT	European Union Drug Regulating Authorities Clinical Trials
FDA	Food and Drug Administration
FSH	Follicle Stimulating Hormone
g	Grams
G-CSF	Granulocyte Colony Stimulating Factor
GCP	Good Clinical practice

GDPR	General Data Protection Regulation
GGT	Gamma Glutamyl Transferase
GLP	Good Laboratory Practice
GM-CSF	Granulocyte Macrophage Colony-Stimulating Factor
GMP	Good Manufacturing Practice
GP	General Practitioner
HB	Hepatitis B
HBsAg	Hepatitis B Surface Antigen
hCG	Human Chorionic Gonadotropin
HCV	Hepatitis C Virus
HCV Ab	Hepatitis C Virus Antibody
HDM	House Dust Mite
HED	Human Equivalent Dose
HIV	Human Immunodeficiency Virus
HR	Heart rate
HRT	Hormone Replacement Therapy
IB	Investigator’s Brochure
ICF	Informed Consent Form
ICH	International Council for Harmonisation
IEC	Independent Ethics Committees
IFN	Interferon
IL	Interleukin
IMP	Investigational Medicinal Product
INR	International normalized ratio
ISF	Investigator Site File
IUD	Intrauterine Device
IUS	Intrauterine Hormone-Releasing System
IV	Intravenous
JPAC	Joint United Kingdom blood Transfusion and Tissue Transplantation Services Professional Advisory Committee
kg	Kilogram
LDH	Lactate Dehydrogenase
L/min	Litres per minute
LSS	Lesion Severity Score
m^2	Meters squared
MAD	Multiple Ascending Dose
MCH	Mean Cell Haemoglobin
MCHC	Mean Cell Haemoglobin Concentration
MCV	Mean Cell Volume
MDI	Metered Dose Inhaler
MedDRA®	Medical Dictionary for Regulatory Activities®
MEU	Medicines Evaluation Unit
Mg	Milligrams
MHRA	Medicines and Healthcare Products Regulatory Agency

mmHg	Millimeter of mercury
MRSD	Maximum Recommended Starting Dose
msec	Millisecond
ng	Nanogram(s)
nM	Nanomolar
NOAEL	No-Observed-Adverse-Effect Level
NSAID	Nonsteroidal anti-inflammatory drugs
OTC	Over the Counter
PAD	Pharmacologically Active Dose
PASI	Psoriasis Area and Severity Index
PCR	Polymerase Chain Reaction
PD	Pharmacodynamic
PDE	Phosphodiesterase
PGA	Physician Global Assessment
PI	Principal Investigator
PIS	Participant Information Sheet
PK	Pharmacokinetic(s)
PT	Prothrombin time
PVC	Packed Cell Volume
QC	Quality control
QD	quaque die (once a day)
QP	Qualified Person
QTc	Corrected value of the interval between the Q and T waves on the electrocardiogram tracing
QTcF	QT Interval Corrected for Heart Rate Using Fridericia's Formula
RBC	Red Blood Cell
REC	Research Ethics Committee
RNA	Ribonucleic acid
SAC	Safety Advisory Committee
SAD	Single Ascending Dose
SAE	Serious Adverse Event
SAF	Safety Analysis Set
SAP	Statistical Analysis Plan
SARS-CoV-2	Severe Acute Respiratory Syndrome Coronavirus 2
SC	Subcutaneous
SOA	Schedule of Activities
SOC	System Organ Class
SOP	Standard Operating Procedure
SQN	Syne Qua Non
SRM	Study Reference Manual
SS	Steady State
STAT	Signal Transducer and Activator of Transcription
SUSAR	Suspected Unexpected Serious Adverse Reaction
$t_{1/2}$	Elimination Half Life

TB	Tuberculosis
TDL	The Doctor's Laboratory
TEAE	Treatment-emergent adverse event
TID	ter in die (three times a day)
t_{max}	Time to Reach Maximum Concentration
TMF	Trial master file
TPO	Thrombopoietin
UK	United Kingdom
ULN	Upper Limit of Normal
WBC	White Blood Cell
WHO	World Health Organisation
WOCBP	Woman of childbearing potential
µg	Micrograms
µM	Micromolar
µL	Microlitre
